# Supplementary material for: Cluster randomised controlled trial to investigate the effectiveness and cost-effectiveness of a Structured Health Intervention For Truckers (the SHIFT study): a study protocol
Source: BMJ Open. 2019 Nov 24;9(11):e030175. doi: 10.1136/bmjopen-2019-030175 (PMC6886973; doi:10.1136/bmjopen-2019-030175)
Supplement: Author's manuscript [file bmjopen-2019-030175.draft_revisions.pdf]

BMJ Open

BMJ Open is committed to open peer review. As part of this commitment we make the peer review history of every article we publish publicly available.

When an article is published we post the peer reviewers' comments and the authors' responses online. We also post the versions of the paper that were used during peer review. These are the versions that the peer review comments apply to.

The versions of the paper that follow are the versions that were submitted during the peer review process. They are not the versions of record or the final published versions. They should not be cited or distributed as the published version of this manuscript.

BMJ Open is an open access journal and the full, final, typeset and author-corrected version of record of the manuscript is available on our site with no access controls, subscription charges or pay-per-view fees (http://bmjopen.bmj.com).

If you have any questions on BMJ Open's open peer review process please email info.bmjopen@bmj.com

BMJ Open

A cluster randomised controlled trial to investigate the effectiveness and cost-effectiveness of a Structured Health Intervention For Truckers (The SHIFT Study): Study Protocol

Journal:	*BMJ Open*
Manuscript ID	bmjopen-2019-030175
Article Type:	Protocol
Date Submitted by the Author:	01-Mar-2019

Complete List of Authors:	Clemes, Stacy; Loughborough University, School of Sport, Exercise and Health Sciences<br>Varela Mato, Verónica; Loughborough University, School of Sport, Exercise and Health Sciences<br>Munir, Fehmidah; Loughborough University, School of Sport, Exercise and Health Sciences<br>Edwardson, Charlotte; University of Leicester, Diabetes Research Centre<br>Chen, Yu-Ling; Loughborough University, School of Sport, Exercise and Health Sciences<br>Hamer, Mark; Loughborough University, School of Sport, Exercise and Health Sciences<br>Gray, Laura; University of Leicester, Department of Health Sciences<br>Bhupendra Jaicim, Nishal; University of Leicester, Leicester Clinical Trials Unit<br>Richardson, Gerry; University of York, Centre for Health Economics<br>Johnson, Vicki; University Hospitals of Leicester NHS Trust, Leicester Diabetes Centre<br>Troughton, Jacqui; University Hospitals of Leicester NHS Trust, Leicester Diabetes Centre<br>Yates, Thomas; University of Leicester, Diabetes Research Centre<br>King, James; Loughborough University, School of Sport, Exercise and Health Sciences

Keywords:	PUBLIC HEALTH, PREVENTIVE MEDICINE, Physical activity, Sedentary behaviour, Health promotion, Occupational drivers

SCHOLARONE™ Manuscripts

A cluster randomised controlled trial to investigate the effectiveness and cost-effectiveness of a Structured Health Intervention For Truckers (The SHIFT Study): Study Protocol

Stacy A Clemes[1,2], Veronica Varela-Mato[1,2], Fehmidah Munir[1,2], Charlotte L Edwardson[2,3], Yu-Ling Chen[1,2], Mark Hamer[1,2], Laura J Gray[4], Nishal Bhupendra Jaicim[5], Gerry Richardson[6], Vicki Johnson[7], Jacqui Troughton[7], Thomas Yates[2,3], James King[1,2]

[1]National Centre for Sport and Exercise Medicine, School of Sport, Exercise and Health Sciences, Loughborough University, Loughborough, UK

[2]NIHR Leicester Biomedical Research Centre, UK

[3]Diabetes Research Centre, University of Leicester, Leicester, UK

[4]Department of Health Sciences, University of Leicester, Leicester, UK

[5]Leicester Clinical Trials Unit, University of Leicester, Leicester, UK

[6]Centre for Health Economics, University of York, York, UK

[7]Leicester Diabetes Centre, University Hospitals of Leicester NHS Trust, Leicester, UK

Corresponding author: Stacy Clemes

Postal address: National Centre for Sport and Exercise Medicine, School of Sport, Exercise and Health Sciences, Loughborough University, Loughborough, UK

Email: S.A.Clemes@lboro.ac.uk

Telephone number: +44 (0)1509 228170

For peer review only - http://bmjopen.bmj.com/site/about/guidelines.xhtml

ABSTRACT

Introduction: Heavy goods vehicle (HGV) drivers' exhibit higher than nationally representative rates of obesity, and obesity-related co-morbidities, in comparison to other occupational groups. Their working environments are not conducive to a healthy lifestyle, yet there has been limited attention to health promotion efforts. We have developed a Structured Health Intervention For Truckers (the SHIFT programme), a multicomponent, theory-driven, health-behaviour intervention targeting physical activity, diet, and sitting in HGV drivers. This paper describes the protocol of a cluster randomised controlled trial designed to evaluate the effectiveness and cost-effectiveness of the SHIFT programme.

Methods and analysis: HGV drivers will be recruited from a logistics company in the UK. Following baseline measurements, depots (clusters) will be randomised to either the SHIFT intervention or usual-care control arm (12 clusters in each, average cluster size 14 drivers). The 6-month SHIFT intervention includes a group-based interactive 6-hour education session, worksite champion support, and equipment provision (including a Fitbit® and resistance bands/balls to facilitate a 'cab workout'). Objectively measured total daily physical activity (steps/day) will be the primary outcome. Secondary outcomes include: objectively measured light-intensity physical activity and moderate-vigorous physical activity, sitting time, sleep quality, markers of adiposity, blood pressure, and capillary blood markers (HbA1c, LDL-HDL-cholesterol). Self-report questionnaires will examine fruit and vegetable intake, psychosocial and work outcomes, and mental health. Quality of life and resources used (e.g. GP visits) will also be assessed. Measures will be collected at baseline, 6 and 12-months and analysed according to a modified intention-to-treat principle. A full process evaluation and cost-effectiveness analysis will be conducted.

For peer review only - http://bmjopen.bmj.com/site/about/guidelines.xhtml

Ethics and dissemination: Ethical approval was obtained from the Loughborough University Ethics Approvals Sub-Committee (Reference: R17-P063). Study findings will be disseminated through publications in research and professional journals, through conference presentations, and to relevant regional and national stakeholders via online media and at dissemination events.

Trial Registration: ISRCTN10483894 (date registered: 1st March, 2017)

Strengths and limitations of this study

- To our knowledge, this is the first RCT to examine the impact of a multicomponent intervention targeting individual and environmental barriers faced by HGV drivers to lead a healthy lifestyle.
- The trial will involve a full process evaluation and an economic evaluation.
- The primary outcome, physical activity, will be objectively measured.
- Sustainability of the intervention will be examined at 6-month follow-up, following completion of the intervention.
- Due to the nature of the intervention, participants will not be blinded to their treatment arm.

For peer review only

INTRODUCTION

Long-distance heavy goods vehicle (HGV) drivers are exposed to a multitude of health-related risk factors associated with their occupation; as a result, lorry driving has been identified as one of the most hazardous working professions.[1 2] Drivers' working environment provides limited opportunities for a healthy lifestyle and unhealthy lifestyle behaviours, such as a lack of physical activity, prolonged periods of sedentary behaviour (sitting), poor diet, a high prevalence of smoking, high volumes of alcohol consumption, and irregular sleeping patterns are widespread among this occupational group.[2-4] Furthermore, long and variable working hours and tight delivery schedules within the Logistics and Transport industry contribute to psychological stress and sleep deprivation.[2 5]

Long-distance drivers exhibit higher than nationally representative rates of obesity, with observational data from a sample of HGV drivers from the UK demonstrating that 84% were overweight or obese, compared to 75% of males aged 45-54 years reported to be overweight/obese nationally.[6] Similar data have been reported from US HGV drivers.[2 7] The high rates of overweight and obesity in HGV drivers elevates their risk of numerous chronic diseases and conditions, including cardiovascular disease, type 2 diabetes, obstructive sleep apnoea and musculoskeletal disorders.[1 2 7-9]

To compound the high-risk health profile observed in long-distance drivers nationally and internationally,[1 2 7-9] the driver population in the UK (n = ~300,000) has been identified as an ageing workforce (mean age: 48 years).[10] A recent Parliamentary report has highlighted the 'demographic time bomb' the UK logistics industry is currently facing and the health impact of an ageing, at-risk, workforce 'driving a vehicle often referred to as 'a 40-tonne missile''.[11] The UK logistics sector is also experiencing a short-fall in HGV drivers, with

For peer review only - http://bmjopen.bmj.com/site/about/guidelines.xhtml

barriers to recruitment including the lack of roadside facilities, medical concerns and long hours of work.[12] Recommendations on how to address this shortfall and attract younger employees to the sector include increasing awareness within the industry of the need to address driver health risks and health behaviours.[11]

A systematic review[13] of health promotion interventions in truck drivers, including only 8 studies, observed that the interventions generally led to improvements in health and health behaviours. However, it was concluded that the strength of the evidence was limited due to poor study designs, with no control groups, small samples and no or limited follow-up periods.[13] Since the publication of the systematic review, recent studies have examined the impacts of a weight loss intervention in US HGV drivers[14] and a smartphone application on physical activity and diet in Australian HGV drivers.[15] Whilst positive findings were observed, the studies were limited by relatively small samples and no comparison groups.

We have developed a Structured Health Intervention For Truckers (the SHIFT programme), a multicomponent, theory driven, health behaviour intervention designed to promote positive lifestyle changes in relation to physical activity, diet, and sitting in HGV drivers. This intervention has been informed by extensive stakeholder engagement, including a qualitative study exploring the perceived barriers to healthy lifestyle behaviours in HGV drivers,[5] an observational study exploring lifestyle health-related behaviours in HGV drivers and markers of health,[3] and a pre-post pilot intervention[16] with full process evaluation.[17] Initial pre-post testing of the intervention revealed the SHIFT programme lead to favourable changes in physical activity and some markers of health.[16] This protocol paper describes a study which will build on our earlier work and generate new knowledge on the effectiveness and cost-effectiveness of the SHIFT programme, evaluated using a

For peer review only - http://bmjopen.bmj.com/site/about/guidelines.xhtml

cluster randomised controlled trial (RCT) design with immediate and extended follow-up. Specifically, we will examine the impact of the SHIFT programme on physical activity, sedentary behaviour, fruit and vegetable intake, adiposity, sleep duration and quality, risk factors for cardio-metabolic disease, psychosocial outcomes and mental health in a sample of HGV drivers.

STUDY AIM AND OBJECTIVES

The aim of this study is to evaluate the effectiveness and cost-effectiveness of the SHIFT programme using a cluster RCT.

Primary objective

To investigate the impact of the SHIFT programme, compared to usual care, on objectively measured physical activity (expressed as steps/day) at 12-months follow-up.

Secondary objectives

To investigate the impact of the SHIFT programme, compared to usual care, at 12-months follow-up on;

1. time spent in light and moderate-to-vigorous physical activity (MVPA)
2. sitting time
3. measures of adiposity (BMI, percent body fat, waist-hip ratio, neck circumference)
4. blood pressure
5. cardiometabolic risk markers (e.g. HBA1c, total cholesterol, HDL-C and LDL-C)
6. fruit and vegetable intake
7. sleep
8. cognitive function and psychophysiological reactivity

For peer review only - http://bmjopen.bmj.com/site/about/guidelines.xhtml

For peer review only

9. psychosocial variables and mental health (e.g. anxiety and depression, work engagement, job performance and satisfaction, presenteeism, sickness absence, health-related quality of life, and driving related safety behaviour)

We will also conduct a full process evaluation (secondary objective 10) and a full economic evaluation (secondary objective 11).

METHODS AND ANALYSIS

Design

The design of this study is based on guidance from the UK Medical Research Council for developing and evaluating complex interventions,[18] and this protocol paper has been prepared following the recommendations within the SPIRIT (Standard Protocol Items for Randomized Trials) statement.[19] This is a workplace two-armed 12-month cluster RCT, which will incorporate an internal pilot, and include both economic and process evaluations. Clusters (different worksites/depots within the same company) will be randomised, following the completion of baseline measurements, to receive either the 'SHIFT programme' or usual care condition. The impact of the intervention will be assessed at 6 and 12-months after randomisation. Figure 1 shows the overall trial design.

Insert Figure 1 about here

Setting

This research will take place within the worksite setting of a major international Logistics and Transport company. The Logistics and Posts Sector is worth approximately £55 billion to the UK economy and currently employs approximately 1.7 million people. Driving is a fundamental occupation within this industry, and drivers and warehouse workers make up the majority of the workforce within the industry.[11]

For peer review only - http://bmjopen.bmj.com/site/about/guidelines.xhtml

Depot recruitment and exclusion criteria

Depots will be included in the study if they contain at least 20 long-distance HGV drivers (see sample size). Depots containing HGV drivers who make many delivery stops, for example, drivers who deliver consumer goods to domestic customers throughout the day will be excluded. For logistical reasons, depots located within the Midlands region of the UK will be recruited. Our partner company has approximately 40 sites, containing approximately 1700 HGV drivers within this region. These sites are a similar size, and have a similar variation in size, to the company's national-level data. During recruitment, depots will be informed that they will have a 50% chance of being randomised to a current practice control condition.

Participant recruitment and exclusion criteria

All HGV drivers within participating depots will be eligible to participate, unless they meet the following exclusion criteria: suffering from clinically diagnosed cardiovascular disease, or mobility limitations that prevent them from increasing their daily activity levels, haemophilia, or have any blood-borne viruses. Posters advertising the study will be placed in participating depots for up to four weeks prior to the scheduling of baseline measurements. In addition, all drivers within participating depots will receive a letter and participant information sheet informing them of the study. Following the distribution of the study marketing material, researchers will visit participating depots for one to two days to enable interested drivers to ask any questions about the study before signing up. Upon completion of these visits the researchers will provide a list of drivers' names who have agreed to participate to their Transport Managers who will then schedule time for participating drivers to attend the baseline (and follow-up) measurements.

For peer review only - http://bmjopen.bmj.com/site/about/guidelines.xhtml

Sample size

Our earlier exploratory pre-post study revealed that on average HGV drivers achieve 8786 steps/day across both workdays and non-workdays with a standard deviation of 2919 steps.[16] We have powered this study to look for a difference in step counts (the primary outcome) of 1500 steps/day (equivalent to approximately 15 minutes of moderately paced walking) between the intervention group and control group. Evidence demonstrates a linear association between step counts and a range of morbidity and mortality outcomes, as well as with markers of health status including inflammation and adiposity, insulin sensitivity and HDL cholesterol in adults.[20-22] The linear association between step counts and health outcomes indicate that regardless of an individual's baseline value, even modest increases in daily step counts can yield clinically meaningful health benefits. For example, a difference in daily steps of 1500 steps/day has been associated with around a 5-10% lower risk of all-cause mortality and cardiovascular morbidity and mortality in the general population and in those with a high risk of type 2 diabetes respectively.[23 24] The proposed level of change has been chosen based on findings from our exploratory pre-post intervention,[16] whilst also being clinically meaningful.

Based on a cluster size of 10, a conservative ICC of 0.05 (as there is no previous data to inform this, we have been informed by recommendations of Campbell et al.[25]), an alpha of 0.05, power of 80% and a coefficient of variation to allow for variation in cluster size of 0.51 (based on partner company data) we will require 110 participants from 11 clusters per arm. From experience in conducting such studies, it is estimated that retention and compliance rates will be approximately 70% at 12-months follow-up; therefore, the sample size will be inflated by 30% to ensure we have adequate power in our final analysis. We will also inflate the number of clusters by 2 to allow for whole cluster drop out. We will therefore recruit 24 clusters with an average of 14 participants per cluster.

For peer review only - http://bmjopen.bmj.com/site/about/guidelines.xhtml

Intervention – The SHIFT Programme

The SHIFT programme is a multicomponent lifestyle-behaviour intervention designed to target behaviour changes in physical activity, diet and sitting in HGV drivers. This 6-month intervention, grounded within the Social Cognitive Theory for behaviour change[26] consists of a group-based (4-6 participants) 6-hour structured education session tailored for HGV drivers, delivered by two trained educators. It includes information about physical activity, diet and sitting and risk factors for type 2 diabetes and cardiovascular disease. The educational component is founded on the approach used in the award winning suite of DESMOND programmes, including the PREPARE[27] and Let's Prevent Diabetes programmes,[28] created by researchers at the Leicester Diabetes Centre and used throughout the NHS,[29] whilst being tailored to meet the needs of HGV drivers.[5] Within the education session participants will not be 'taught' in a formal way, but supported to work out knowledge through group discussions and to develop individual goals and plans, based on detailed individual feedback received during their health assessments (see Measurements) to achieve over the 6-month intervention period. The education session is supported by specially developed resources for HGV drivers and participant support materials. The session will include the discussion of feasible strategies for participants to increase their physical activity, improve their diet and reduce their sitting time (when not driving) during working and non-working hours. The content of the educational session is summarised in Table 1.

Insert Table 1 about here

During the education session, participants will be provided with a Fitbit® Charge 2 activity tracker and encouraged to use this to set goals (agreed at the session) to gradually

For peer review only - http://bmjopen.bmj.com/site/about/guidelines.xhtml

increase their physical activity predominately through walking-based activity. The Fitbit® activity tracker will provide participants with information on their daily step counts and will be used as a tool for self-monitoring and self-regulation. Physical activity tracking using step counters (traditionally pedometers) has been associated with significant reductions in BMI and blood pressure, with interventions incorporating goal setting being the most effective.[30]

The education session will adopt the promotion of the "small changes" philosophy using the Specific, Measurable, Attainable, Relevant, and Timely (SMART) principle[31] to encourage participants to gradually build-up their daily activity levels, within the confines of their occupation, to meet the current UK Physical Activity guidelines.[32] For example, participants will be encouraged to establish their own personalised action plan, which may also include making dietary improvements in addition to increases in physical activity, with SMART goals throughout the 6-month intervention. 'Step count challenges' (1-week competitions within intervention depots) will run every 6-weeks throughout the 6-month intervention which will be facilitated by local worksite champions. A "cab workout" will be introduced and practised at the education session and participants will be provided with resistance bands and balls, and grip strength dynamometers to take away. Participants will be encouraged to undertake the cab workout during breaks when not permitted to leave their vehicle. Participants will be able to keep the intervention tools beyond the 6-month intervention period, however the company will choose whether to sustain the worksite champion support and step count challenges beyond the 6-month intervention period. A Logic Model detailing the underlying theory behind the intervention components is shown in Figure 2.

For peer review only - http://bmjopen.bmj.com/site/about/guidelines.xhtml

The structured education session will be delivered by trained personnel from our partner company and by trained members of the research team. These individuals will be trained and mentored by trainers from the Leicester Diabetes Centre. The education sessions will take place within appropriate training rooms within the intervention depots. Personnel delivering the education sessions within each intervention depot will also be trained to act as a local champion, shown to enhance the effectiveness of worksite physical activity interventions.[33] They will provide ongoing health coach support to intervention participants (during the 6-month intervention period) and be responsible for facilitating the step count challenges.

Insert Figure 2 about here

Control arm

Depots assigned to the usual practice control arm will be asked to continue with their usual care conditions. Participants in the control depots will receive an educational leaflet at the outset detailing the importance of healthy lifestyle behaviours (i.e., undertaking regular physical activity, breaking up periods of prolonged sitting, and consuming a healthy diet) for the promotion of health and well-being. Control participants will be requested to complete the same study measurements as those in the intervention worksites, at the same time points. Upon completion of the study, control depots will be provided with all of the educational material provided to the intervention participants as part of the SHIFT programme. As the intervention will be delivered by trained personnel within our partner company, the company may choose to provide the full intervention (including the education session and health coach support) to control depots upon completion of the formal trial.

For peer review only - http://bmjopen.bmj.com/site/about/guidelines.xhtml

For peer review only

Allocation to treatment groups

Clusters (depots within the same company) will be randomised at the worksite level into the two study arms (intervention and control, using an allocation ratio of 1:1). Randomisation into the study arms will take place in two batches; initially the first 6 clusters (depots) involved in the internal pilot (see below) will be randomised, and in the second batch all of the remaining clusters will be randomised stratified by depot size. Depots will be classified as either small or large based on the median cluster size of all participating depots. In both batches randomisation will take place upon completion of baseline measurements and will be done by an independent statistician at the Leicester Clinical Trials Unit (CTU).

Measurements

The outcome measurements will be assessed at 3 time points. Baseline measures will occur prior to randomisation of the depots into the 2 study arms. A second set of identical measurements will take place 6-months post randomisation (i.e. just after the completion of the 6-month intervention), and a final set will be taken at 12-months post randomisation to assess the sustainability of the intervention (i.e. 6 months after completion of the formal intervention period, as recommended by the National Obesity Observatory[34]). At the baseline assessment, the study will be explained to the participant and written informed consent will be obtained. The measurements will be undertaken in suitable rooms within participating depots by trained researchers and will last between 1.5 and 2 hours per participant. Participants will complete a range of self-report questionnaires and have a series of physiological health assessments taken. All participants will receive detailed feedback on their physiological health assessment measures during each measurement session. In the event that a potential health issue is evident during the health assessments, such as undiagnosed hypertension or high cholesterol levels, participants

For peer review only - http://bmjopen.bmj.com/site/about/guidelines.xhtml

will be advised to visit their GP for further checks. We will provide participants with a letter to give to their GP which summarises the findings from our point-of-care (blood markers) and automated (blood pressure) measures. Participants will be requested to inform the researchers about the use of any prescribed medications that they commence throughout the study duration which may impact the proposed outcome measures. Participants will be issued with objective monitoring devices to assess their free-living physical activity, sedentary behaviour and sleep, which they will be instructed to wear for eight days following each measurement visit. After eight days, participants will be requested to return these monitors to their depot where they will be collected by a member of the research team.

Primary outcome

The primary outcome will be physical activity, expressed as steps/day, at 12 months post randomisation. Physical activity will be objectively measured using the activPAL micro accelerometer, worn continuously on the anterior aspect of the thigh, for 24 hours/day over eight days during each assessment period. The activPAL provides a valid measure of walking and posture (i.e. sitting and standing) in adults,[35-37] and provides a more accurate measure of physical activity and sitting in occupational drivers in comparison to waist-worn accelerometers.[38] As the physical activity component of the intervention predominantly includes the promotion of walking based-activity, and as participants will be provided with a Fitbit providing information on daily step counts to set goals to increase their physical activity, steps/day was chosen as the primary physical activity related outcome.

Secondary outcomes

A number of secondary outcomes will be assessed at all measurement time points. The secondary outcomes are described below:

For peer review only - http://bmjopen.bmj.com/site/about/guidelines.xhtml

Physical activity and sedentary behaviour

Light and moderate-to-vigorous physical activity (MVPA) will be assessed using the activPAL and the wrist-worn GENEActiv accelerometer, both worn continuously for eight days. The GENEActiv is a lightweight waterproof device, resembling a sports watch, which has been found to be a valid and reliable objective measure of physical activity.[39] Outcomes calculated from the GENEActiv include minutes spent in MVPA, proportion of participants meeting the MVPA guidelines of 150 minutes per/week, total volume of physical activity regardless of intensity, and sleep duration. The accelerometer provides time stamped data so activity at specific times of the day (e.g., during work, after work) will also be extracted.

Sedentary behaviour will also be measured for 8 consecutive days during each assessment period using the activPAL3 micro. The activPAL is regarded as the most accurate method of assessing sitting behaviour in free-living settings,[37 40 41] and is recommended for use in interventions when sitting is an outcome measure.[36] From the data provided, we will extract total daily sitting time, work-time and leisure-time sitting, sitting bout durations, and number of transitions between sitting and standing.

Sleep duration, subjective sleepiness and chronotype

Sleep duration and efficiency will be measured objectively using the GENEActiv which has been shown to be an accurate measure of sleep, in addition to physical activity.[42] Subjective sleepiness will be assessed using the Karolinska Sleepiness Scale, shown to be a valid measure of sleepiness when validated against electroencephalography (EEG)

For peer review only - http://bmjopen.bmj.com/site/about/guidelines.xhtml

and performance outcomes.[43 44] Participants' chronotype will be determined using the short version of the Morningness-Eveningness questionnaire.[45]

Anthropometry, adiposity and blood pressure

Stature (measured at baseline only) and body mass (both assessed without shoes), along with waist and hip circumferences, will be measured using standardised anthropometric techniques by trained research staff. BMI will be calculated as weight (kg)/height (m^2). Body composition (percentage body fat and fat mass) will be assessed via bio-impedance analysis, using Tanita DC-360S body composition scales. We will also measure neck circumference which is a novel marker which links strongly to obstructive sleep apnoea, insulin resistance and cardiovascular disease risk.[46] Blood pressure will be measured from the left arm after a twenty minute period of quiet sitting using an automated recorder (Omron HEM-907), in accordance with current recommendations.[47]

Biochemical assessments

Finger-prick blood samples will be collected from participants, with participants being requested to fast for ≥4 hours prior to attending each health assessment. The 'A1CNow®+ point-of-care analyser will be used to measure glycated haemoglobin which is a marker of long-term glucose regulation used in clinical care. Additionally, we will use the Cardiochek® point-of-care analyser to measure circulating cholesterol (total, HDL, LDL). Both of these systems are manufactured by PTS Diagnostics and possess analyte validation certificates from the International Federation of Clinical Chemistry and Laboratory Medicine.

For peer review only - http://bmjopen.bmj.com/site/about/guidelines.xhtml

Functional fitness

Grip strength will be assessed from both hands using the Takei Hand-Grip dynamometer (Takei Scientific Instruments Co., Ltd; Japan). Reduced muscular strength, as measured by grip strength, is associated with an increased risk of cardiovascular disease, and all-cause and cardiovascular mortality.[48]

Cognitive function and psychophysiological reactivity

The Stroop test will be administered over a five minute period using a validated software package to provide a measure of reaction time, sensitivity to interference and the ability to suppress an automated response - reading colour names in favour of naming the font colour.[49] To examine psychophysiological reactivity, acute stress will be induced using a five-minute mirror-tracing task (Campden Instruments Ltd.), during which measures of blood pressure and heart rate will be taken.[50]

Work-related psychosocial variables and mental health

A series of self-report measures will be employed to characterise work-related health and mental health: musculoskeletal symptoms will be assessed using the Standardised Nordic Questionnaire;[51] work engagement (characterized by vigour, dedication, and absorption) will be measured using the Utrecht Work Engagement Scale (UWES);[52] occupational fatigue will be measured using the Occupational Fatigue Exhaustion Recovery (OFER 15) scale;[53] job performance[54] and job satisfaction[55] will be measured using single-item 7-point Likert scales; sickness presenteeism will be assessed using a single-item questionnaire; participant's perceptions of work demand and support will be assessed using four subscales from the Health and Safety Executive Management Standards Indicator Tool

For peer review only - http://bmjopen.bmj.com/site/about/guidelines.xhtml

(HSE MSIT),[56] and driving-related safety behaviour will be assessed using a 6-item measure.[57] Anxiety and depression will be measured using the Hospital Anxiety and Depression Scale (HADS),[58] and Social Isolation will be assessed using the 8-item Social Isolation short form from the Patient-Reported Outcomes Measurement Information System.[59 60] Data on sickness absence will be collected via self-report and employer records and will include frequency and duration of self-certified and certified sickness. Data on sickness absence will be collected from organisational records for 12 months prior to the intervention and for the 6-month intervention and follow-up periods.

Health-related quality of life and health-related resource use

The self-reported EQ5D[61] will be completed by participants during each assessment period to inform the within-trial cost-effectiveness analysis (see cost-effectiveness). Participants will also complete a questionnaire, developed for this study, assessing health-related resource use at the same time points.

Demographics and additional lifestyle health-related behaviour measures

At baseline we will collect basic demographic information for each participant including their date of birth, sex, ethnicity, highest level of education, marital status, postcode (to determine Index of Multiple Deprivation as an indicator of neighbourhood socio-economic status), working hours, years worked as a HGV driver, and years worked at our partner company. At each follow-up assessment, participants will be asked if there have been any changes in these variables. During each assessment, information on smoking status and typical alcohol intake will be gathered by self-report measures. Dietary quality, including

For peer review only

For peer review only - http://bmjopen.bmj.com/site/about/guidelines.xhtml

fruit and vegetable intake, will be assessed using a short-form food frequency questionnaire.[62]

Internal pilot

We intend to conduct an internal pilot study using the first six clusters (depots). The internal pilot will examine issues surrounding worksite and participant recruitment, randomisation, compliance to the primary outcome, and retention rates at 6-months following randomisation. After this period we will continue to the full trial if the following progression criteria are met:

• All 24 depots required for the full sample size agree to take part in the study. Six depots will be selected to take part in the internal pilot (three will be randomised to the intervention arm and three to the control arm). This will demonstrate that depot recruitment and intervention delivery is on-track.

• According to our criteria, 84 drivers will need to agree to participate in the internal pilot, based on an average of 14 participants per cluster.

• An average of 75% of drivers opting into the study, randomised into the intervention arm, attend the education session across the 3 intervention depots. This figure is based on the intervention uptake rate seen in our exploratory pre-post intervention study (87%),[16] whilst also recognising that take-up rates tend to be lower when moving from an efficacy to a larger multi-centre effectiveness trial.

• No more than 20% of participants fail to provide valid data for the primary outcome measure (activPAL-determined step counts) at baseline and at 6 months post randomisation or withdraw/are lost to follow-up during the six-month intervention phase. This threshold is necessary as study power requires total withdrawal or loss to follow-up of no higher than 30% during the six-month intervention and six-month follow-up (12 months post randomisation).

For peer review only - http://bmjopen.bmj.com/site/about/guidelines.xhtml

If the final two progression criteria are not fully met, strategies to improve these metrics for the full trial will be discussed with the Trial Steering Committee and the study will progress based upon recommendations from this committee.

Process evaluation

The process evaluation will be used to help explain any discrepancies between expected and observed outcomes, to understand the influence of intervention components and context on the observed outcomes, and to provide insight for any further intervention development and implementation.[18] Throughout the intervention, within each intervention depot, we will monitor the fidelity of the intervention implementation using the Normalisation Process Theory framework,[63] in line with guidance from the National Institutes of Health Behaviour Change Consortium and the DESMOND collaborative. We will employ a variety of techniques (e.g., log books, questionnaires, interviews and focus groups) to inform our process evaluation. For example, Transport Managers (or their nominated facilitators) and educators/worksite champions from each site will report on a monthly basis if there were any organisational changes (e.g. job changes) or events which may affect participation. Self-report questionnaires provided to study participants will evaluate the various intervention components (e.g. education session, physical activity monitoring tool, cab workout). Interviews and focus groups with study participants will further examine engagement in the various components of the intervention, along with any perceived barriers or facilitators to participating in these components. Interviews and focus groups with worksite champions, HR staff, health and safety personnel and logistics timetabling and planning staff will further examine the intervention implementation. We will also document any environmental factors (e.g. movement of personnel between worksites/depots, potential contamination of the intervention through drivers in different groups meeting at service stations/customer distribution centres) that may have an

For peer review only - http://bmjopen.bmj.com/site/about/guidelines.xhtml

influence on intervention effectiveness. Details of the process evaluation components are included in Table 2.

Insert Table 2 about here

Patient and Public Involvement (PPI)

This trial is the result of an earlier 3-year partnership between the research team and a large transport and logistics company (different to our partner in the present study) in the East Midlands, UK. The preparatory work which informed this study[3 5 16 17] was instigated by the company who requested help in improving the lifestyle behaviours and health of their long-distance drivers who were proving difficult to engage. The SHIFT programme was developed in collaboration with long-distance HGV drivers and health and safety personnel working within the logistics sector. Following pilot testing, the intervention and outcome measures described within this protocol, have been refined following further input from drivers and associated stakeholders. A driver and manager working within the logistics sector will sit on our independent Trial Steering Committee and will provide invaluable insight into the design, set-up, conduct and dissemination of this research. Throughout the trial we will conduct regular PPI events with relevant stakeholders to gain feedback on the trial's progress. The research team will also continue to work with the Chartered Institute of Logistics & Transport to facilitate research dissemination (articles, conferences, workshops) across the logistics and transport sector nationally and internationally.

Data Analysis

Statistical analysis - Internal pilot

For peer review only - http://bmjopen.bmj.com/site/about/guidelines.xhtml

The average recruitment rate across depots, proportion of participants providing valid data, and attendance rate at the education sessions will be reported with 95% CI. The point estimates and 95% CIs will be compared to the progression criteria outlined above.

Statistical analysis - Main trial

Average daily steps at 12-months will be compared by group using generalised estimating equation models adjusted for baseline values and waking wear time with an exchangeable correlation structure, which adjusts for clustering. For the primary analysis missing data will not be replaced (complete case analysis) but participants will be included in the intervention group in which their depots were randomised irrespective of the intervention actually received (modified intention-to-treat analysis). We have inflated our sample size by 30% to account for potential loss to follow-up and non-compliance with the primary outcome measure. We will compare the baseline characteristics of those who have complete primary outcome data and those who do not. A sensitivity analysis using multiple imputation will be performed to assess the impact of missing outcome data on the results found and to account for uncertainty associated with imputing data (full intention to treat analysis). The imputation will be carried out using the command MI in Stata. MI replaces missing values with multiple sets of simulated values to complete the data, performs standard analysis on each completed dataset, and adjusts the obtained parameter estimates for missing-data uncertainty using Rubin’s rules to combine estimates. The effect size will also be assessed by attendance excluding those who did not attend the full intervention (per-protocol analysis). Secondary outcomes and 6-month data will be analysed using similar methodology.

Qualitative analyses

For peer review only - http://bmjopen.bmj.com/site/about/guidelines.xhtml

Audio-recordings of interviews and focus groups with drivers, worksite champions, HR staff, health and safety personnel and logistics timetabling and planning staff will be transcribed verbatim and analysed using framework analysis,[64 65] using the Normalisation Process Theory[63] as the overarching framework.

Cost-effectiveness

The economic analysis will consist of a cost-consequence analysis based on the observed results within the trial period and a cost-effectiveness analysis where differences between groups in the trial will be extrapolated to the longer term. For both analyses, costs in both arms will be estimated from a NHS and Personal Social Services (PSS) perspective (consistent with that used by NICE) as well as a wider public sector perspective. In each analysis, the cost of the SHIFT arm will include an estimate of the cost of the intervention (including the cost of training the educators), generated through a staff questionnaire completed at the end of each education session.

Within-trial analysis

Within the trial, resource use estimates will be collected from participant questionnaires and will include health related resource use as well as absence from employment. The health-related resource use will be based on a variant of the Client Service Receipt Inventory and will include services that this population are likely to utilise such as GPs and Practise nurse appointments, occupational health visitors and counsellors. Costs of resources will be calculated by applying published national unit cost estimates (e.g. NHS reference costs or PSSRU Unit costs of health and social care[66 67]), where available, to estimates of relevant resource use.

For peer review only - http://bmjopen.bmj.com/site/about/guidelines.xhtml

A range of outcomes will be assessed in the trial including health related quality of life, measured using the EQ5D.[61] The within trial analysis will present incremental results for the primary and secondary outcomes (including EQ5D) in both intervention and control arms and will be compared with the incremental costs measured above. We will also present the results in terms of the differences between the groups in time absent from work. Two analyses will be conducted, one including these productivity losses, the other excluding them. This will allow decision makers to assess the importance of inclusion of these costs in the adoption decision.

Longer-term analysis

It is acknowledged that although there may be short term health benefits from the intervention, the longer-term effects of, for example, increased physical activity on diabetic status and number of cardiovascular events may be more important. We will therefore conduct a brief literature review to identify existing models that link short term endpoints measured in the trial and longer-term quality of life. These models will be utilised to extrapolate costs and effects of the intervention beyond the trial period to a more appropriate time horizon. If appropriate an Incremental Cost-effectiveness Ratio for the extrapolated period will be reported using the Quality Adjusted Life Year (QALY). As with the within-trial analysis, we will conduct analyses where productivity losses are included/excluded to assess the impact on decision making. Costs and effects will be discounted at the prevailing recommended rate (currently 1.5% per annum on both costs and effects), but will be the subject of sensitivity analysis to reflect the ongoing uncertainty around appropriate discount rates for public health interventions. To reflect the levels of uncertainty in parameter inputs we will conduct probabilistic sensitivity analyses; this will allow a characterisation of the uncertainty around the adoption decision which we will depict using cost-effectiveness acceptability curves. Sensitivity analyses will be performed

For peer review only - http://bmjopen.bmj.com/site/about/guidelines.xhtml

to determine the robustness of the results to altering certain assumptions such as the discount rate or inclusion/exclusion of productivity losses.

DISCUSSION

HGV drivers' working environments are not conducive to a healthy lifestyle, despite this they are currently an underserved occupational group in terms of health promotion efforts and exhibit higher than nationally representative rates of obesity and related co-morbidities.[7 13] The health and wellbeing of professional drivers is of public concern given their health impacts the safety of all road users.[13] Of concern, obese HGV drivers are approximately 50% more likely to have an accident than normal weight drivers.[68] The present study will target health-related behaviours of this at-risk and underserved occupational group, with the goal of making a positive long-term impact on long-distance HGV drivers' health. Given the current absence of resources available to tackle health inequalities within the transport sector, it is anticipated that if effective, the SHIFT programme could be scalable as a Continued Professional Competence (CPC) resource for HGV drivers nationally and internationally. This resource will likely be modifiable for use across other driving-related occupations. This could have a long-term impact on professional drivers' health, lead to cost savings within the logistics and transport sector, and ultimately impact road safety for all road users.

To our knowledge, this is the first RCT to examine the impact of a multicomponent intervention targeting individual and environmental barriers faced by HGV drivers to lead a healthy lifestyle. Strengths of the study include the robust RCT design, with randomisation at the depot/cluster level to reduce contamination. The large fully powered sample, objectively-derived outcome measures, and six-month follow-up assessment after

For peer review only - http://bmjopen.bmj.com/site/about/guidelines.xhtml

completion of the SHIFT programme are further strengths, along with the extensive process and economic evaluations.

ETHICS AND DISSEMINATION

Ethical approval was obtained from the Loughborough University Ethics Approvals (Human Participants) Sub-Committee (Reference: R17-P063). Study findings will be disseminated through publications in research and professional journals, through conference presentations at scientific meetings, and to relevant regional and national stakeholders via online media and at dissemination events. Loughborough University are the trial sponsor.

AUTHORS' CONTRIBUTIONS

All authors have made substantial contributions to the concept and design of the study. The study was conceived by SC, VVM, JK, TY, CE, MH, FM, LG, JT, VJ, and GR. VVM, VJ and YLC are the qualified Research Associates on the project. LG and NBJ are the trial statisticians and developed the statistical analysis plan and GR developed the cost-effectiveness plan. The first draft of this manuscript was written by SC with input from all other authors. All authors have edited and critically reviewed the paper for intellectual content and approved the final version of the paper.

FUNDING STATEMENT

This study is funded by the NIHR Public Health Research Programme (reference: NIHR PHR 15/190/42) and supported by the NIHR Leicester Biomedical Research Centre – Lifestyle theme. The views expressed are those of the authors and not necessarily those of the NHS, the NIHR or the Department of Health and Social Care. Funding to cover

For peer review only - http://bmjopen.bmj.com/site/about/guidelines.xhtml

intervention costs (Fitbits, cab workout equipment) has been received from the Higher Education Innovation Fund, via the Loughborough University Enterprise Projects Group.

COMPETING INTERESTS STATEMENT

None declared.

WORD COUNT: 5773

REFERENCES

1. Apostolopoulos Y, Sonmez S, Shattell MM, Belzer M. Worksite-induced morbidities among truck drivers in the United States. *AAOHN J* 2010;**58**(7):285-96.

2. Sieber WK, Robinson CF, Birdsey J, Chen GX, Hitchcock EM, Lincoln JE, et al. Obesity and other risk factors: the national survey of U.S. long-haul truck driver health and injury. *Am J Ind Med.* 2014;**57**(6):615-26.

3. Varela-Mato V, O'Shea O, King JA, Yates T, Stensel DJ, Biddle SJ, et al. Cross-sectional surveillance study to phenotype lorry drivers' sedentary behaviours, physical activity and cardio-metabolic health. *BMJ Open* 2017;**7**(6):e013162.

4. Apostolopoulos Y, Sonmez S, Shattell M, Haldeman L, Strack R, Jones V. Barriers to Truck Drivers' Healthy Eating: Environmental Influences and Health Promotion Strategies. *Journal of Workplace Behavioral Health* 2011;**26**:122-43.

5. Caddick N, Varela-Mato V, Nimmo MA, Clemes S, Yates T, King JA. Understanding the health of lorry drivers in context: A critical discourse analysis. *Health* 2017;**21**(1):38-56.

6. Health and Social Care Information Centre. Health Survey for England 2014.

7. Thiese MS, Moffitt G, Hanowski RJ, Kales SN, Porter RJ, Hegmann KT. Commercial Driver Medical Examinations: Prevalence of Obesity, Comorbidities, and Certification Outcomes. *J Occup Environ Med* 2015;**57**(6):659-65.

For peer review only - http://bmjopen.bmj.com/site/about/guidelines.xhtml

For peer review only

8. Martin BC, Church TS, Bonnell R, Ben-Joseph R, Borgstadt T. The impact of overweight and obesity on the direct medical costs of truck drivers. *J Occup Environ Med* 2009;**51**(2):180-4.

9. Bigert C, Gustavsson P, Hallqvist J, Hogstedt C, Lewne M, Plato N, et al. Myocardial infarction among professional drivers. *Epidemiology* 2003;**14**(3):333-9.

10. The Freight Transport Association. Logistics Report 2018.

11. The All Party Parliamentary Group for Freight Transport. Barriers to Youth Employment in the Freight Transport Sector, 2015.

12. The Freight Transport Association. Logistics Report 2015.

13. Ng M, Yousuf B, Bigelow P, Van Eerd D. Effectiveness of health promotion programmes for truck drivers: A systematic review. *Health Education Journal* 2015;**74**(3):270-86.

14. Thiese MS, Effiong AC, Ott U, Passey DG, Arnold ZC, Ronna BB, et al. A Clinical Trial on Weight Loss among Truck Drivers. *Int J Occup Environ Med* 2015;**6**(2):104-12.

15. Gilson ND, Pavey TG, Vandelanotte C, Duncan MJ, Gomersall SR, Trost SG, et al. Chronic disease risks and use of a smartphone application during a physical activity and dietary intervention in Australian truck drivers. *Aust N Z J Public Health* 2016;**40**(1):91-3.

16. Varela Mato V, Caddick N, King JA, Johnson V, Edwardson C, Yates T, et al. The Impact of a Novel Structured Health Intervention for Truckers (SHIFT) on Physical Activity and Cardiometabolic Risk Factors. *J Occup Environ Med* 2018;**60**(4):368-76.

17. Varela Mato V, Caddick N, King JA, Yates T, Stensel DJ, Nimmo MA, et al. A Structured Health Intervention for Truckers (SHIFT): A Process Evaluation of a Pilot Health Intervention in a Transport Company. *J Occup Environ Med* 2018;**60**(4):377-85.

18. Craig P, Dieppe P, Macintyre S, Michie S, Nazareth I, Petticrew M. Developing and evaluating complex interventions: the new Medical Research Council guidance. *Int J Nurs Stud* 2013;**50**(5):587-92.

For peer review only - http://bmjopen.bmj.com/site/about/guidelines.xhtml

19. Chan AW, Tetzlaff JM, Altman DG, Laupacis A, Gotzsche PC, Krleza-Jeric K, et al. SPIRIT 2013 statement: defining standard protocol items for clinical trials. *Ann Intern Med* 2013;**158**(3):200-7.

20. Ewald B, Attia J, McElduff P. How many steps are enough? Dose-response curves for pedometer steps and multiple health markers in a community-based sample of older Australians. *J Phys Act Health* 2014;**11**(3):509-18.

21. Thomson JL, Landry AS, Zoellner JM, Tudor-Locke C, Webster M, Connell C, et al. Several steps/day indicators predict changes in anthropometric outcomes: HUB City Steps. *BMC Public Health* 2012;**12**:983.

22. Dwyer T, Ponsonby AL, Ukoumunne OC, Pezic A, Venn A, Dunstan D, et al. Association of change in daily step count over five years with insulin sensitivity and adiposity: population based cohort study. *BMJ* 2011;**342**:c7249.

23. Dwyer T, Pezic A, Sun C, Cochrane J, Venn A, Srikanth V, et al. Objectively Measured Daily Steps and Subsequent Long Term All-Cause Mortality: The Tasped Prospective Cohort Study. *PloS One* 2015;**10**(11):e0141274.

24. Yates T, Haffner SM, Schulte PJ, Thomas L, Huffman KM, Bales CW, et al. Association between change in daily ambulatory activity and cardiovascular events in people with impaired glucose tolerance (NAVIGATOR trial): a cohort analysis. *Lancet* 2014;**383**(9922):1059-66.

25. Campbell MK, Fayers PM, Grimshaw JM. Determinants of the intracluster correlation coefficient in cluster randomized trials: the case of implementation research. *Clin Trials* 2005;**2**(2):99-107.

26. Bandura A. Health promotion by social cognitive means. *Health Educ Behav* 2004;**31**(2):143-64.

For peer review only

27. Yates T, Davies M, Gorely T, Bull F, Khunti K. Effectiveness of a pragmatic education program designed to promote walking activity in individuals with impaired glucose tolerance: a randomized controlled trial. *Diabetes Care* 2009;**32**(8):1404-10.

28. Gray LJ, Yates T, Troughton J, Khunti K, Davies MJ, Let's Prevent Diabetes T. Engagement, Retention, and Progression to Type 2 Diabetes: A Retrospective Analysis of the Cluster-Randomised "Let's Prevent Diabetes" Trial. *PLoS Medicine* 2016;**13**(7):e1002078.

29. Davies MJ, Heller S, Skinner TC, Campbell MJ, Carey ME, Cradock S, et al. Effectiveness of the diabetes education and self management for ongoing and newly diagnosed (DESMOND) programme for people with newly diagnosed type 2 diabetes: cluster randomised controlled trial. *BMJ* 2008;**336**(7642):491-5.

30. Bravata DM, Smith-Spangler C, Sundaram V, Gienger AL, Lin N, Lewis R, et al. Using pedometers to increase physical activity and improve health: a systematic review. *JAMA* 2007;**298**(19):2296-304.

31. Doran GT. There's a S.M.A.R.T. Way to Write Management's Goals and Objectives. *Management Review* 1981;**70**(11):35-36.

32. Chief Medical Officers of England, Scotland, Wales, and Northern Ireland. Start active, stay active: a report on physical activity from the four home countries' Chief Medical Officers. Crown copyright, 2011.

33. Conn VS, Hafdahl AR, Cooper PS, Brown LM, Lusk SL. Meta-analysis of workplace physical activity interventions. *Am J Prev Med* 2009;**37**(4):330-9.

34. Cavill N, Roberts K, Rutter H. Standard Evaluation Framework for Physical Activity Interventions. Oxford: National Obesity Observatory, 2012.

35. Ryan CG, Grant PM, Tigbe WW, Granat MH. The validity and reliability of a novel activity monitor as a measure of walking. *Br J Sports Med* 2006;**40**(9):779-84.

For peer review only - http://bmjopen.bmj.com/site/about/guidelines.xhtml

36. Kozey-Keadle S, Libertine A, Lyden K, Staudenmayer J, Freedson PS. Validation of wearable monitors for assessing sedentary behavior. *Med Sci Sports Exerc* 2011;**43**(8):1561-7.

37. Atkin AJ, Gorely T, Clemes SA, Yates T, Edwardson C, Brage S, et al. Methods of Measurement in epidemiology: Sedentary Behaviour. *Int J Epidemiol* 2012;41(5):1460-71.

38. Varela Mato V, Yates T, Stensel D, Biddle S, Clemes SA. Concurrent Validity of Actigraph-Determined Sedentary Time Against the Activpal Under Free-Living Conditions in a Sample of Bus Drivers. *Measurement in Physical Education and Exercise Science* 2017;**21**(4):212-22.

39. Esliger DW, Rowlands AV, Hurst TL, Catt M, Murray P, Eston RG. Validation of the GENEA Accelerometer. *Med Sci Sports Exerc* 2011;**43**(6):1085-93.

40. Edwardson C, Winkler E, Bodicoat DH, Yates T, Davies M, Dunstan D, et al. Considerations when using the activPAL monitor in field-based research with adult populations. *J Sport Health Sci* 2017;**6**:162–78.

41. Edwardson CL, Rowlands AV, Bunnewell S, Sanders J, Esliger DW, Gorely T, et al. Accuracy of Posture Allocation Algorithms for Thigh- and Waist-Worn Accelerometers. *Med Sci Sports Exerc* 2016;**48**(6):1085-90.

42. Buman MP, Hu F, Newman E, Smeaton AF, Epstein DR. Behavioral Periodicity Detection from 24 h Wrist Accelerometry and Associations with Cardiometabolic Risk and Health-Related Quality of Life. *Biomed Res Int* 2016:4856506.

43. Akerstedt T, Gillberg M. Subjective and objective sleepiness in the active individual. *Int J Neurosci* 1990;**52**(1-2):29-37.

44. Kaida K, Takahashi M, Akerstedt T, Nakata A, Otsuka Y, Haratani T, et al. Validation of the Karolinska sleepiness scale against performance and EEG variables. *Clin Neurophysiol* 2006;**117**(7):1574-81.

For peer review only

For peer review only - http://bmjopen.bmj.com/site/about/guidelines.xhtml

45. Horne JA, Ostberg O. A self-assessment questionnaire to determine morningness-eveningness in human circadian rhythms. *Int J Chronobiol* 1976;**4**(2):97-110.

46. Preis SR, Massaro JM, Hoffmann U, D'Agostino RB, Sr., Levy D, Robins SJ, et al. Neck circumference as a novel measure of cardiometabolic risk: the Framingham Heart study. *J Clin Endocrinol Metab* 2010;**95**(8):3701-10.

47. O'Brien E, Asmar R, Beilin L, Imai Y, Mallion JM, Mancia G, et al. European Society of Hypertension recommendations for conventional, ambulatory and home blood pressure measurement. *J Hypertens* 2003;**21**(5):821-48.

48. Leong DP, Teo KK, Rangarajan S, Lopez-Jaramillo P, Avezum A, Jr., Orlandini A, et al. Prognostic value of grip strength: findings from the Prospective Urban Rural Epidemiology (PURE) study. *Lancet* 2015;**386**(9990):266-73.

49. MacLeod CM. Half a century of research on the Stroop effect: an integrative review. *Psychol Bull* 1991;**109**(2):163-203.

50. Wawrzyniak AJ, Hamer M, Steptoe A, Endrighi R. Decreased reaction time variability is associated with greater cardiovascular responses to acute stress. *Psychophysiology* 2016;**53**:739-48.

51. Kuorinka I, Jonsson B, Kilbom A, Vinterberg H, Biering-Sorensen F, Andersson G, et al. Standardised Nordic questionnaires for the analysis of musculoskeletal symptoms. *Appl Ergon* 1987;**18**(3):233-7.

52. Schaufeli W, Salanova M, González-Romá V, Bakker A. The measurement of Engagement and burnout: A confirmative analytic approach. *Journal of Happiness Studies* 2002;**3**:71-92.

53. Winwood PC, Lushington K, Winefield AH. Further development and validation of the Occupational Fatigue Exhaustion Recovery (OFER) scale. *J Occup Environ Med* 2006;**48**(4):381-9.

For peer review only

For peer review only - http://bmjopen.bmj.com/site/about/guidelines.xhtml

54. Bond FW, Bunce D. Job control mediates change in a work reorganization intervention for stress reduction. *J Occup Health Psychol* 2001;**6**(4):290-302.

55. Nagy M. Using a single-item approach to measure facet job satisfaction. *J Org Occup. Psychol.* 2002;**75**:77–86.

56. Health and Safety Executive. Health and Safety Executive Management Standards Indicator Tool, 2013.

57. Huang Y, Roetting M, McDevitt J, Melton D, Smith G. Feedback by technology: Attitudes and opinions of truck drivers. *Transportation Research Part F: Traffic Psychology and Behavior* 2005;**8**:277–97.

58. Zigmond AS, Snaith RP. The hospital anxiety and depression scale. *Acta Psychiatr Scand.* 1983;**67**(6):361-70.

59. Cella D, Gershon R, Lai JS, Choi S. The future of outcomes measurement: item banking, tailored short-forms, and computerized adaptive assessment. *Qual Life Res* 2007;**16** Suppl 1:133-41.

60. Johnston KL, Lawrence SM, Dodds NE, Yu L, Daley DC, Pilkonis PA. Evaluating PROMIS(R) instruments and methods for patient-centered outcomes research: Patient and provider voices in a substance use treatment setting. *Qual Life Res* 2016;**25**(3):615-24.

61. EuroQol G. EuroQol--a new facility for the measurement of health-related quality of life. *Health Policy* 1990;**16**(3):199-208.

62. Cleghorn CL, Harrison RA, Ransley JK, Wilkinson S, Thomas J, Cade JE. Can a dietary quality score derived from a short-form FFQ assess dietary quality in UK adult population surveys? *Public Health Nutr* 2016; **19**(16):2915-2923.

63. May C, Murray E, Finch T, Mair F, Treweek S, Ballini L, et al. Normalization Process Theory On-line Users' Manual and Toolkit. Available from http://www.normalizationprocess.org/, 2010.

For peer review only

For peer review only - http://bmjopen.bmj.com/site/about/guidelines.xhtml

64. Ritchie J, Spencer L. Qualitative data analysis for applied policy research. In: Bryman and Burgess, editor. *Analysing Qualitative Data*. London: Routledge, 1994:173-94.

65. Gale NK, Heath G, Cameron E, Rashid S, Redwood S. Using the framework method for the analysis of qualitative data in multi-disciplinary health research. *BMC Med Res Methodol* 2013;**13**:117.

66. Department of Health. NHS reference costs 2012-2013. London, 2013.

67. Personal Social Services Research Unit. Unit Costs of Health and Social Care 2015, University of Kent, 2015.

68. Anderson JE, Govada M, Steffen TK, Thorne CP, Varvarigou V, Kales SN, et al. Obesity is associated with the future risk of heavy truck crashes among newly recruited commercial drivers. *Accid Anal Prev* 2012;**49**:378-84.

69. Chaiken S. The heuristic model of persuation. In: Zanna M, Olson J, Herman C, editors. *Social influence: the Ontaio symposium.* Fifth Edition ed. NJ: Erlbaum, 1987:3-39.

70. Leventhal H, Meyer D, Nerenz D. The common-sense representation of illness danger. In: Rachman S, editor. *Contributions to Medical Psychology.* Second Edition. New York: Pergamon, 1980: 7–30.

71. Bandura A. Self-efficacy: toward a unifying theory of behavioral change. *Psychol Rev* 1977;**84**(2):191-215.

For peer review only

Table 1. Outline of the educational component of the SHIFT programme

Section name	Theoretical underpinning	Main aims and educator activities	Duration (minutes)
Welcome and Introduction		Participants introduced to the SHIFT programme and made aware of both content and style of the session.	10
Driver Story	Dual Process theory,[69] Common Sense Model[70]	Participants asked about their beliefs about how being a HGV driver can affect health, the causes of these health problems and controllability of these.	30
Risks and Health problems	Dual Process theory,[69] Common Sense Model,[70] Social learning theory[71]	Facilitator uses participant stories to support them to work out why they may be at risk of future health problems, and what to do to reduce/ manage risk	55
Physical Activity	Dual Process theory,[69] Social learning theory[71]	Facilitator supports participants to develop knowledge and skills to support confidence to increase personal activity levels, to set personal goals and self-monitor through the use of Fitbits. Introduction and practical demonstration of the 'cab-workout'.	80
Depression, Sleeping, Smoking	Dual Process theory,[69] Social learning theory[71]	Facilitator supports participants to develop strategies to manage depression, poor sleep and smoking	30
Food Choices	Dual Process theory,[69] Social learning theory[71]	Facilitator supports participants to develop knowledge and skills for food choices to reduce cardiovascular risk factors and improve overall health.	90
Self-management plan	Dual Process theory,[69] Social learning theory[71]	Participants supported in developing personal self-management plans.	15
Questions	Common Sense Model,[70] Social learning theory[71]	Facilitator checks all questions raised by participants throughout the programme have been answered and understood.	5
What happens next	Social learning theory[71]	Follow-up care outlined	5

For peer review only - http://bmjopen.bmj.com/site/about/guidelines.xhtml

Table 2. Process evaluation plan for the SHIFT intervention

**Areas to measure**	**General process questions**	**Data source and data collection method**	**Total numbers and sampling strategy/timescales**
Recruitment	Number of depots/worksites invited to participate, and number agreeing<br><br>Number of possible participants at each depot, number invited/recommended for participation, number opting in to the intervention<br><br>Number of participants opting-out, dropping out and non-compliance to the primary outcome measure	Project records, including the number of drivers within each depot approached<br><br>Depot logs of staff numbers, project records, attendance records at measurements<br><br>Participant attendance records, short questionnaires to explore reasons for non-participation, dropping out and non-compliance	On-going throughout the project
Acceptability of randomisation and measurement tools	How depots feel about being randomised to intervention / control arms<br><br>Did participants find outcome assessments acceptable<br><br>How did participants and logistics timetabling staff experience recruitment and timetabling of outcome assessments	Focus groups with participants<br><br>Interviews with local depot health and safety advisors/HR/timetabling staff	~8 focus groups, or until data saturation is reached, with participants ~1 month following completion of baseline measures<br><br>~8 interviews, or until data saturation is reached, with local depot health and safety advisors/HR/timetabling staff ~1 month after completion of baseline measures in their depots
Intervention acceptability and fidelity - implementation	Was the intervention implemented as planned<br><br>How did participants and logistics timetabling staff experience scheduling the education sessions	Interviews with personnel within our logistics partners who are trained as educators and implemented the education sessions<br><br>Interviews with local worksite champions and timetabling staff within intervention depots<br><br>Participant questionnaires	Interviews with educators, the number of which will depend on the number of educators trained, and timetabling staff immediately following delivery of the education sessions<br><br>Interviews with local champions 3 months into the intervention, immediately following the intervention (6 months), and at 9 and 12 months<br><br>Questionnaires administered after education sessions to participants
Intervention acceptability and fidelity - participation	What proportion of the target group participated in the intervention, and what components of the intervention were preferred, did this differ between males and females	Focus groups with intervention participants<br><br>Attendance logs at education sessions and measurement visits	~8 focus groups, or until data saturation is reached, with participants immediately following completion of the intervention (6 months)

For peer review only

Areas to measure	General process questions	Data source and data collection method	Total numbers and sampling strategy/timescales
	What strategies were put in place by intervention participants to facilitate behaviour change	Questionnaires and focus groups	Brief questionnaires administered to all intervention participants at 6 months during health assessments
Intervention sustainability	What proportion of the target group maintained any changes in their health behaviours following the 6 month intervention period<br><br>Were there any differences in sustainability between males and females<br><br>Are the company going to continue with the intervention in some way	Focus groups with intervention participants<br><br>Questionnaires<br><br>Interviews with health and safety personnel	~8 focus groups, or until data saturation is reached, with participants at 10 months follow-up (4 months after completion of the intervention.<br><br>Brief questionnaires administered to all intervention participants at 12 months during health assessments<br><br>Interviews at 12 months
Intervention contamination	Did movement of staff (e.g. participants, health and safety personnel) occur from intervention to control depots<br><br>Did intervention drivers interact with control drivers at customer warehouses/distribution centres etc.	Control depots to keep a log of any staff changes<br><br>Focus groups with intervention and control participants	Logs collected upon completion of the 12 month follow-up assessments<br><br>8 focus groups, or until data saturation is reached, with intervention and control participants immediately following completion of the intervention (6 months) and at 10 months follow-up
Unexpected events arising from the study	Did intervention and control participants modify their behaviours based on information provided at the baseline health assessments?<br><br>Did the health assessments prompt GP visits<br><br>Did increased self-awareness of health status and constraints within the job lead to cognitive dissonance<br><br>Did intervention participants change an existing activity-related behaviour for another as a result of participating in the study	Focus groups, interviews and questionnaires delivered to intervention and control participants	Questionnaires delivered to intervention and control participants 1 month after completion of the baseline health assessments<br><br>8 focus groups, or until data saturation is reached, with intervention and control participants immediately following completion of the intervention (6 months) and at 10 months follow-up<br><br>One-to one interviews based on questionnaire and focus group responses at 1 and 10 months

For peer review only - http://bmjopen.bmj.com/site/about/guidelines.xhtml

Figure 1. Trial design and participant flow through the study

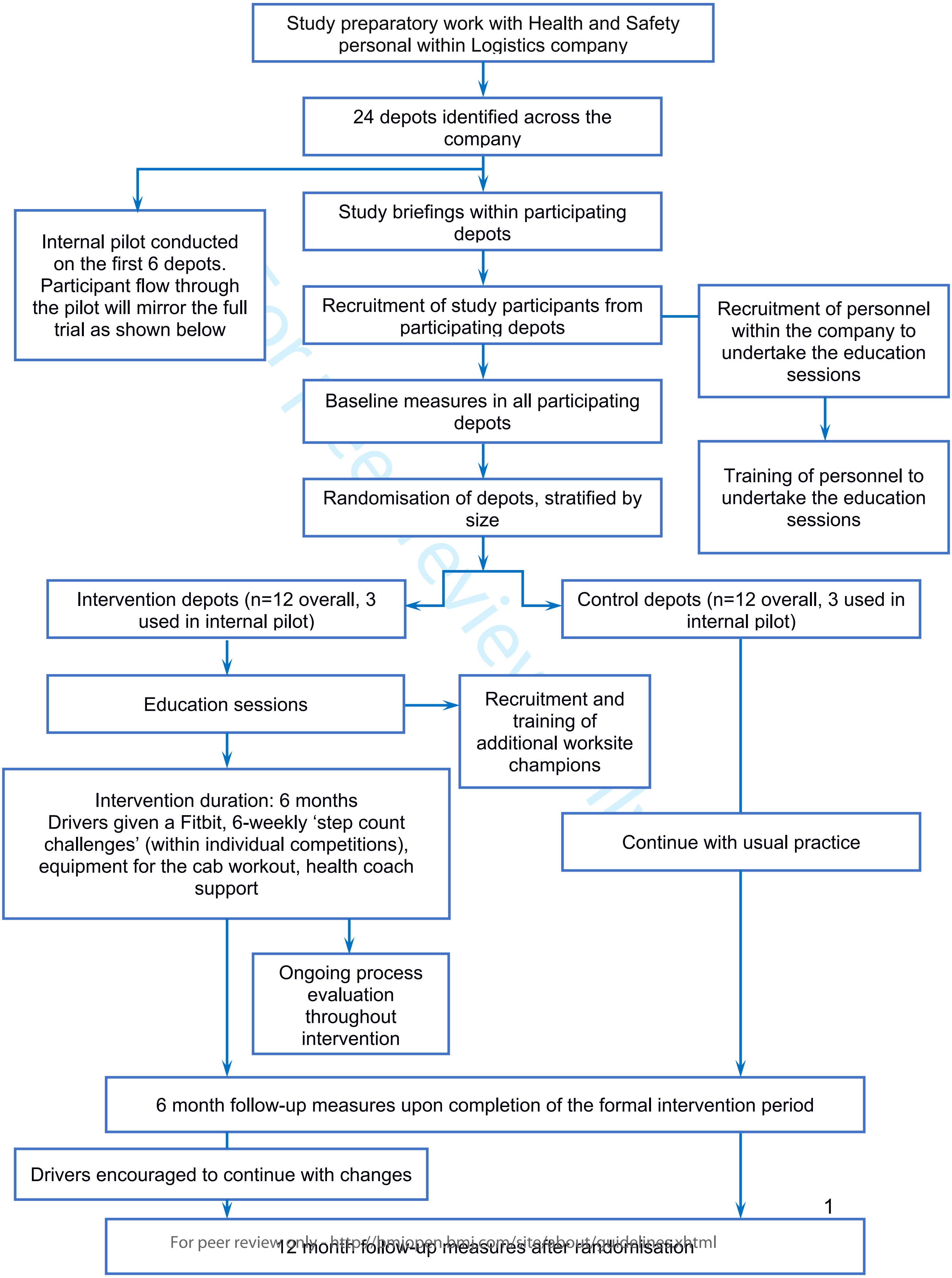

1

Figure 2. Logic model for the SHIFT intervention

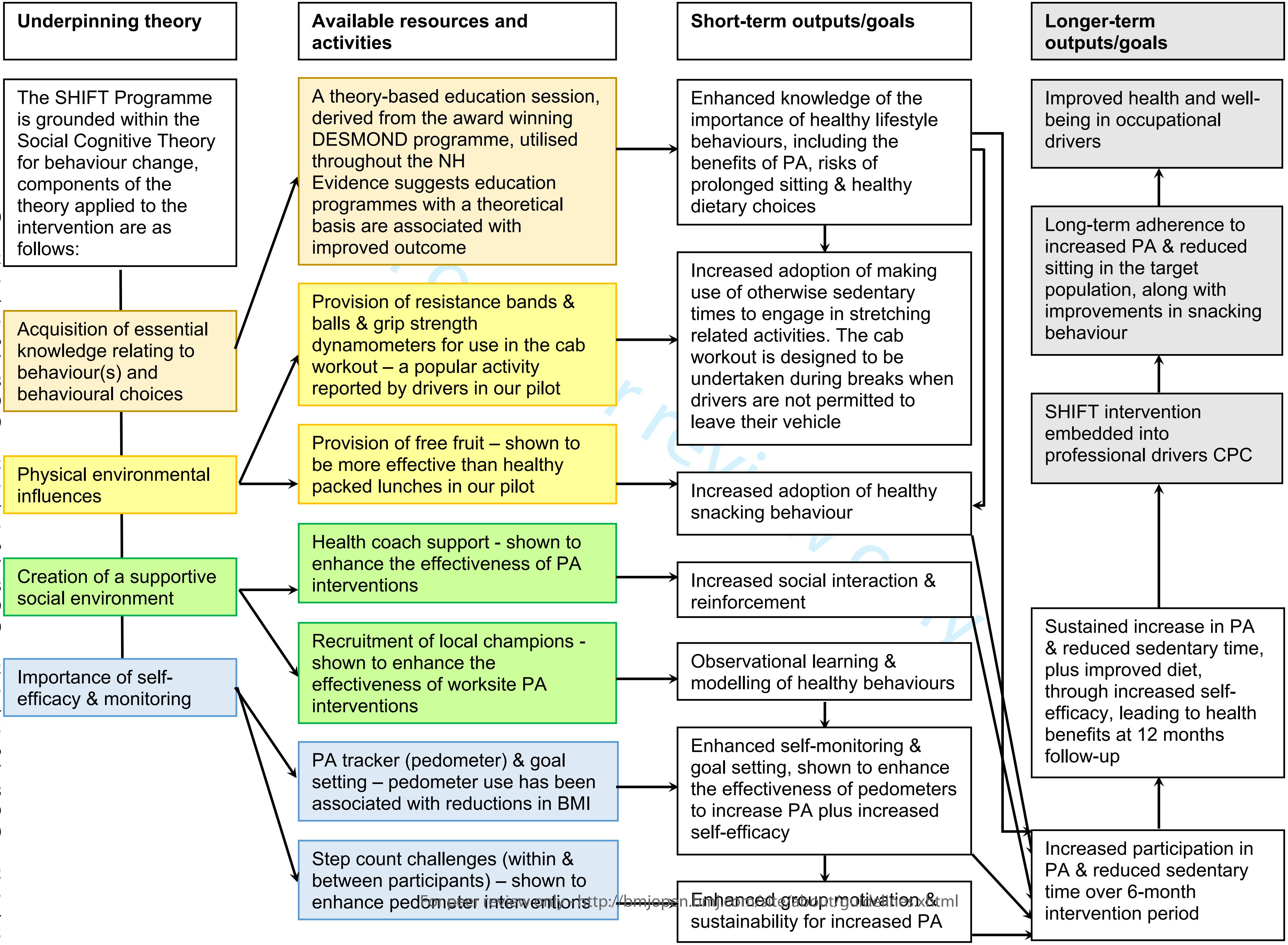

1

For peer review only - http://bmjopen.bmj.com/site/about/guidelines.xhtml

SPIRIT
STANDARD PROTOCOL ITEMS: RECOMMENDATIONS FOR INTERVENTIONAL TRIALS

SPIRIT 2013 Checklist: Recommended items to address in a clinical trial protocol and related documents*

Section/item	Item No	Description	Addressed on page number
**Administrative information**			
Title	1	Descriptive title identifying the study design, population, interventions, and, if applicable, trial acronym	1
Trial registration	2a	Trial identifier and registry name. If not yet registered, name of intended registry	3
	2b	All items from the World Health Organization Trial Registration Data Set	N/A
Protocol version	3	Date and version identifier	N/A
Funding	4	Sources and types of financial, material, and other support	26 - 27
Roles and responsibilities	5a	Names, affiliations, and roles of protocol contributors	1 & 26
	5b	Name and contact information for the trial sponsor	26
	5c	Role of study sponsor and funders, if any, in study design; collection, management, analysis, and interpretation of data; writing of the report; and the decision to submit the report for publication, including whether they will have ultimate authority over any of these activities	26
	5d	Composition, roles, and responsibilities of the coordinating centre, steering committee, endpoint adjudication committee, data management team, and other individuals or groups overseeing the trial, if applicable (see Item 21a for data monitoring committee)	21

For peer review only

**Introduction**			
Background and rationale	6a	Description of research question and justification for undertaking the trial, including summary of relevant studies (published and unpublished) examining benefits and harms for each intervention	4 - 6
	6b	Explanation for choice of comparators	12
Objectives	7	Specific objectives or hypotheses	6 - 7
Trial design	8	Description of trial design including type of trial (eg, parallel group, crossover, factorial, single group), allocation ratio, and framework (eg, superiority, equivalence, noninferiority, exploratory)	7
**Methods: Participants, interventions, and outcomes**			
Study setting	9	Description of study settings (eg, community clinic, academic hospital) and list of countries where data will be collected. Reference to where list of study sites can be obtained	7
Eligibility criteria	10	Inclusion and exclusion criteria for participants. If applicable, eligibility criteria for study centres and individuals who will perform the interventions (eg, surgeons, psychotherapists)	8
Interventions	11a	Interventions for each group with sufficient detail to allow replication, including how and when they will be administered	10 - 12
	11b	Criteria for discontinuing or modifying allocated interventions for a given trial participant (eg, drug dose change in response to harms, participant request, or improving/worsening disease)	N/A
	11c	Strategies to improve adherence to intervention protocols, and any procedures for monitoring adherence (eg, drug tablet return, laboratory tests)	20
	11d	Relevant concomitant care and interventions that are permitted or prohibited during the trial	N/A
Outcomes	12	Primary, secondary, and other outcomes, including the specific measurement variable (eg, systolic blood pressure), analysis metric (eg, change from baseline, final value, time to event), method of aggregation (eg, median, proportion), and time point for each outcome. Explanation of the clinical relevance of chosen efficacy and harm outcomes is strongly recommended	14 - 19
Participant timeline	13	Time schedule of enrolment, interventions (including any run-ins and washouts), assessments, and visits for participants. A schematic diagram is highly recommended (see Figure)	Figure 1

For peer review only - http://bmjopen.bmj.com/site/about/guidelines.xhtml

Sample size	14	Estimated number of participants needed to achieve study objectives and how it was determined, including clinical and statistical assumptions supporting any sample size calculations	9
Recruitment	15	Strategies for achieving adequate participant enrolment to reach target sample size	9

Methods: Assignment of interventions (for controlled trials)

Allocation:

Sequence generation	16a	Method of generating the allocation sequence (eg, computer-generated random numbers), and list of any factors for stratification. To reduce predictability of a random sequence, details of any planned restriction (eg, blocking) should be provided in a separate document that is unavailable to those who enrol participants or assign interventions	13
Allocation concealment mechanism	16b	Mechanism of implementing the allocation sequence (eg, central telephone; sequentially numbered, opaque, sealed envelopes), describing any steps to conceal the sequence until interventions are assigned	13
Implementation	16c	Who will generate the allocation sequence, who will enrol participants, and who will assign participants to interventions	13
Blinding (masking)	17a	Who will be blinded after assignment to interventions (eg, trial participants, care providers, outcome assessors, data analysts), and how	3
	17b	If blinded, circumstances under which unblinding is permissible, and procedure for revealing a participant's allocated intervention during the trial	N/A

Methods: Data collection, management, and analysis

Data collection methods	18a	Plans for assessment and collection of outcome, baseline, and other trial data, including any related processes to promote data quality (eg, duplicate measurements, training of assessors) and a description of study instruments (eg, questionnaires, laboratory tests) along with their reliability and validity, if known. Reference to where data collection forms can be found, if not in the protocol	14 - 19
	18b	Plans to promote participant retention and complete follow-up, including list of any outcome data to be collected for participants who discontinue or deviate from intervention protocols	14 – 19, 20

Data management	19	Plans for data entry, coding, security, and storage, including any related processes to promote data quality (eg, double data entry; range checks for data values). Reference to where details of data management procedures can be found, if not in the protocol	N/A
Statistical methods	20a	Statistical methods for analysing primary and secondary outcomes. Reference to where other details of the statistical analysis plan can be found, if not in the protocol	21 - 25
	20b	Methods for any additional analyses (eg, subgroup and adjusted analyses)	N/A
	20c	Definition of analysis population relating to protocol non-adherence (eg, as randomised analysis), and any statistical methods to handle missing data (eg, multiple imputation)	22
**Methods: Monitoring**			
Data monitoring	21a	Composition of data monitoring committee (DMC); summary of its role and reporting structure; statement of whether it is independent from the sponsor and competing interests; and reference to where further details about its charter can be found, if not in the protocol. Alternatively, an explanation of why a DMC is not needed	20 & 21
	21b	Description of any interim analyses and stopping guidelines, including who will have access to these interim results and make the final decision to terminate the trial	19 - 20
Harms	22	Plans for collecting, assessing, reporting, and managing solicited and spontaneously reported adverse events and other unintended effects of trial interventions or trial conduct	N/A
Auditing	23	Frequency and procedures for auditing trial conduct, if any, and whether the process will be independent from investigators and the sponsor	20
**Ethics and dissemination**			
Research ethics approval	24	Plans for seeking research ethics committee/institutional review board (REC/IRB) approval	3
Protocol amendments	25	Plans for communicating important protocol modifications (eg, changes to eligibility criteria, outcomes, analyses) to relevant parties (eg, investigators, REC/IRBs, trial participants, trial registries, journals, regulators)	N/A

Consent or assent	26a	Who will obtain informed consent or assent from potential trial participants or authorised surrogates, and how (see Item 32)	13
	26b	Additional consent provisions for collection and use of participant data and biological specimens in ancillary studies, if applicable	N/A
Confidentiality	27	How personal information about potential and enrolled participants will be collected, shared, and maintained in order to protect confidentiality before, during, and after the trial	13
Declaration of interests	28	Financial and other competing interests for principal investigators for the overall trial and each study site	26 - 27
Access to data	29	Statement of who will have access to the final trial dataset, and disclosure of contractual agreements that limit such access for investigators	N/A
Ancillary and post-trial care	30	Provisions, if any, for ancillary and post-trial care, and for compensation to those who suffer harm from trial participation	N/A
Dissemination policy	31a	Plans for investigators and sponsor to communicate trial results to participants, healthcare professionals, the public, and other relevant groups (eg, via publication, reporting in results databases, or other data sharing arrangements), including any publication restrictions	26
	31b	Authorship eligibility guidelines and any intended use of professional writers	N/A
	31c	Plans, if any, for granting public access to the full protocol, participant-level dataset, and statistical code	N/A
**Appendices**			
Informed consent materials	32	Model consent form and other related documentation given to participants and authorised surrogates	N/A
Biological specimens	33	Plans for collection, laboratory evaluation, and storage of biological specimens for genetic or molecular analysis in the current trial and for future use in ancillary studies, if applicable	N/A

For peer review only - http://bmjopen.bmj.com/site/about/guidelines.xhtml

BMJ Open

A cluster randomised controlled trial to investigate the effectiveness and cost-effectiveness of a Structured Health Intervention For Truckers (The SHIFT Study): Study Protocol

Journal:	*BMJ Open*
Manuscript ID	bmjopen-2019-030175.R1
Article Type:	Protocol
Date Submitted by the Author:	02-Jul-2019
Complete List of Authors:	Clemes, Stacy; Loughborough University, School of Sport, Exercise and Health Sciences<br>Varela Mato, Verónica; Loughborough University, School of Sport, Exercise and Health Sciences<br>Munir, Fehmidah; Loughborough University, School of Sport, Exercise and Health Sciences<br>Edwardson, Charlotte; University of Leicester, Diabetes Research Centre<br>Chen, Yu-Ling; Loughborough University, School of Sport, Exercise and Health Sciences<br>Hamer, Mark; Loughborough University, School of Sport, Exercise and Health Sciences<br>Gray, Laura; University of Leicester, Department of Health Sciences<br>Bhupendra Jaicim, Nishal; University of Leicester, Leicester Clinical Trials Unit<br>Richardson, Gerry; University of York, Centre for Health Economics<br>Johnson, Vicki; University Hospitals of Leicester NHS Trust, Leicester Diabetes Centre<br>Troughton, Jacqui; University Hospitals of Leicester NHS Trust, Leicester Diabetes Centre<br>Yates, Thomas; University of Leicester, Diabetes Research Centre<br>King, James; Loughborough University, School of Sport, Exercise and Health Sciences
**Primary Subject Heading**:	Sports and exercise medicine
Secondary Subject Heading:	Occupational and environmental medicine, Public health
Keywords:	PUBLIC HEALTH, PREVENTIVE MEDICINE, Physical activity, Sedentary behaviour, Health promotion, Occupational drivers

SCHOLARONE™
Manuscripts

A cluster randomised controlled trial to investigate the effectiveness and cost-effectiveness of a Structured Health Intervention For Truckers (The SHIFT Study): Study Protocol

Stacy A Clemes[1,2], Veronica Varela-Mato[1,2], Fehmidah Munir[1,2], Charlotte L Edwardson[2,3], Yu-Ling Chen[1,2], Mark Hamer[1,2], Laura J Gray[4], Nishal Bhupendra Jaicim[5], Gerry Richardson[6], Vicki Johnson[7], Jacqui Troughton[7], Thomas Yates[2,3], James King[1,2]

[1]National Centre for Sport and Exercise Medicine, School of Sport, Exercise and Health Sciences, Loughborough University, Loughborough, UK

[2]NIHR Leicester Biomedical Research Centre, UK

[3]Diabetes Research Centre, University of Leicester, Leicester, UK

[4]Department of Health Sciences, University of Leicester, Leicester, UK

[5]Leicester Clinical Trials Unit, University of Leicester, Leicester, UK

[6]Centre for Health Economics, University of York, York, UK

[7]Leicester Diabetes Centre, University Hospitals of Leicester NHS Trust, Leicester, UK

Corresponding author: Stacy Clemes

Postal address: National Centre for Sport and Exercise Medicine, School of Sport, Exercise and Health Sciences, Loughborough University, Loughborough, UK

Email: S.A.Clemes@lboro.ac.uk

Telephone number: +44 (0)1509 228170

For peer review only

ABSTRACT

Introduction: Heavy goods vehicle (HGV) drivers' exhibit higher than nationally representative rates of obesity, and obesity-related co-morbidities, in comparison to other occupational groups. Their working environments are not conducive to a healthy lifestyle, yet there has been limited attention to health promotion efforts. We have developed a Structured Health Intervention For Truckers (the SHIFT programme), a multicomponent, theory-driven, health-behaviour intervention targeting physical activity, diet, and sitting in HGV drivers. This paper describes the protocol of a cluster randomised controlled trial designed to evaluate the effectiveness and cost-effectiveness of the SHIFT programme.

Methods and analysis: HGV drivers will be recruited from a logistics company in the UK. Following baseline measurements, depots (clusters) will be randomised to either the SHIFT intervention or usual-care control arm (12 clusters in each, average cluster size 14 drivers). The 6-month SHIFT intervention includes a group-based interactive 6-hour education session, worksite champion support, and equipment provision (including a Fitbit® and resistance bands/balls to facilitate a 'cab workout'). Objectively measured total daily physical activity (steps/day) will be the primary outcome. Secondary outcomes include: objectively measured light-intensity physical activity and moderate-vigorous physical activity, sitting time, sleep quality, markers of adiposity, blood pressure, and capillary blood markers (HbA1c, LDL-HDL-cholesterol). Self-report questionnaires will examine fruit and vegetable intake, psychosocial and work outcomes, and mental health. Quality of life and resources used (e.g. GP visits) will also be assessed. Measures will be collected at baseline, 6 and 12-months and analysed according to a modified intention-to-treat principle. A full process evaluation and cost-effectiveness analysis will be conducted.

For peer review only

Ethics and dissemination: Ethical approval was obtained from the Loughborough University Ethics Approvals Sub-Committee (Reference: R17-P063). Study findings will be disseminated through publications in research and professional journals, through conference presentations, and to relevant regional and national stakeholders via online media and at dissemination events.

Trial Registration: ISRCTN10483894 (date registered: 1st March, 2017)

Strengths and limitations of this study

- To our knowledge, this is the first RCT to examine the impact of a multicomponent intervention targeting individual and environmental barriers faced by HGV drivers to lead a healthy lifestyle.
- The trial will involve a full process evaluation and an economic evaluation.
- The primary outcome, physical activity, will be objectively measured.
- Sustainability of the intervention will be examined at 6-months follow-up, following completion of the intervention.
- Due to the nature of the intervention, participants will not be blinded to their treatment arm, and there is a risk that the secondary outcome self-report measures may be susceptible to reporting bias.

For peer review only

INTRODUCTION

Long-distance heavy goods vehicle (HGV) drivers are exposed to a multitude of health-related risk factors associated with their occupation; as a result, lorry driving has been identified as one of the most hazardous working professions.[1 2] Drivers' working environment provides limited opportunities for a healthy lifestyle and unhealthy lifestyle behaviours, such as a lack of physical activity, prolonged periods of sedentary behaviour (sitting), poor diet, a high prevalence of smoking, high volumes of alcohol consumption, and irregular sleeping patterns are widespread among this occupational group.[2-5] Furthermore, long and variable working hours, including shift work, and tight delivery schedules within the Logistics and Transport industry contribute to psychological stress and sleep deprivation, which can lead to metabolic disturbances and further promote the uptake of unhealthy behavioural choices.[2 5-8]

Long-distance drivers exhibit higher than nationally representative rates of obesity, with observational data from a sample of HGV drivers from the UK demonstrating that 84% were overweight or obese, compared to 75% of males aged 45-54 years reported to be overweight/obese nationally.[9] Similar data have been reported from US HGV drivers.[2 10] The high rates of overweight and obesity in HGV drivers elevates their risk of numerous chronic diseases and conditions, including cardiovascular disease, type 2 diabetes, obstructive sleep apnoea and musculoskeletal disorders.[1 2 10-13]

To compound the high-risk health profile observed in long-distance drivers nationally and internationally,[1 2 10-12] the driver population in the UK (n = ~300,000) has been identified as an ageing workforce (mean age: 48 years).[14] A recent Parliamentary report has highlighted the 'demographic time bomb' the UK logistics industry is currently facing and the health impact of an ageing, at-risk, workforce 'driving a vehicle often referred to as 'a 40-tonne

For peer review only - http://bmjopen.bmj.com/site/about/guidelines.xhtml

missile''.[15] The UK logistics sector is also experiencing a short-fall in HGV drivers, with barriers to recruitment including the lack of roadside facilities, medical concerns and long hours of work.[16] Recommendations on how to address this shortfall and attract younger employees to the sector include increasing awareness within the industry of the need to address driver health risks and health behaviours.[15]

A systematic review[17] of health promotion interventions in truck drivers, including only 8 studies, observed that the interventions generally led to improvements in health and health behaviours. However, it was concluded that the strength of the evidence was limited due to poor study designs, with no control groups, small samples and no or limited follow-up periods.[17] Since the publication of the systematic review, recent studies have examined the impacts of a weight loss intervention in US HGV drivers[18] and a smartphone application on physical activity and diet in Australian HGV drivers.[19] Whilst positive findings were observed, the studies were limited by relatively small samples and no comparison groups.

We have developed a Structured Health Intervention For Truckers (the SHIFT programme), a multicomponent, theory driven, health behaviour intervention designed to promote positive lifestyle changes in relation to physical activity, diet, and sitting in HGV drivers. This intervention has been informed by extensive stakeholder engagement, including a qualitative study exploring the perceived barriers to healthy lifestyle behaviours in HGV drivers,[6] an observational study exploring lifestyle health-related behaviours in HGV drivers and markers of health,[3] and a pre-post pilot intervention[20] with full process evaluation.[21] Initial pre-post testing of the intervention revealed the SHIFT programme lead to favourable changes in physical activity and some markers of health.[20] This protocol paper describes a study which will build on our earlier work and generate new knowledge

For peer review only - http://bmjopen.bmj.com/site/about/guidelines.xhtml

For peer review only

on the effectiveness and cost-effectiveness of the SHIFT programme, evaluated using a cluster randomised controlled trial (RCT) design with immediate and extended follow-up. Specifically, we will examine the impact of the SHIFT programme on physical activity, sedentary behaviour, fruit and vegetable intake, adiposity, sleep duration and quality, risk factors for cardio-metabolic disease, psychosocial outcomes and mental health in a sample of HGV drivers.

STUDY AIM AND OBJECTIVES

The aim of this study is to evaluate the effectiveness and cost-effectiveness of the SHIFT programme using a cluster RCT.

Primary objective

To investigate the impact of the SHIFT programme, compared to usual care, on objectively measured physical activity (expressed as steps/day) at 12-months follow-up.

Secondary objectives

To investigate the impact of the SHIFT programme, compared to usual care, at 12-months follow-up on;

1. time spent in light and moderate-to-vigorous physical activity (MVPA)
2. sitting time
3. measures of adiposity (BMI, percent body fat, waist-hip ratio, neck circumference)
4. blood pressure
5. cardiometabolic risk markers (e.g. HBA1c, total cholesterol, HDL-C and LDL-C)
6. fruit and vegetable intake
7. sleep
8. cognitive function and psychophysiological reactivity

For peer review only

9. psychosocial variables and mental health (e.g. anxiety and depression, work engagement, job performance and satisfaction, presenteeism, sickness absence, health-related quality of life, and driving related safety behaviour)

We will also conduct a full process evaluation (secondary objective 10) and a full economic evaluation (secondary objective 11).

METHODS AND ANALYSIS

Design

The design of this study is based on guidance from the UK Medical Research Council for developing and evaluating complex interventions,[22] and this protocol paper has been prepared following the recommendations within the SPIRIT (Standard Protocol Items for Randomized Trials) statement.[23] This is a workplace two-armed 12-month cluster RCT, which will incorporate an internal pilot, and include both economic and process evaluations. Clusters (different worksites/depots within the same company) will be randomised, following the completion of baseline measurements, to receive either the 'SHIFT programme' or usual care condition. The impact of the intervention will be assessed at 6 and 12-months after randomisation. Figure 1 shows the overall trial design.

Insert Figure 1 about here

Setting

This research will take place within the worksite setting of a major international Logistics and Transport company. The Logistics and Posts Sector is worth approximately £55 billion to the UK economy and currently employs approximately 1.7 million people. Driving is a fundamental occupation within this industry, and drivers and warehouse workers make up the majority of the workforce within the industry.[15]

For peer review only - http://bmjopen.bmj.com/site/about/guidelines.xhtml

For peer review only

Depot recruitment and exclusion criteria

Depots will be included in the study if they contain at least 20 long-distance HGV drivers (see sample size). Depots containing HGV drivers who make many delivery stops, for example, drivers who deliver consumer goods to domestic customers throughout the day will be excluded. For logistical reasons, depots located within the Midlands region of the UK will be recruited. Our partner company has approximately 40 sites, containing approximately 1700 HGV drivers within this region. These sites are a similar size, and have a similar variation in size, to the company's national-level data. During recruitment, depots will be informed that they will have a 50% chance of being randomised to a current practice control condition.

Participant recruitment and exclusion criteria

All HGV drivers within participating depots will be eligible to participate, unless they meet the following exclusion criteria: suffering from clinically diagnosed cardiovascular disease, or mobility limitations that prevent them from increasing their daily activity levels, haemophilia, or have any blood-borne viruses. Posters advertising the study will be placed in participating depots for up to four weeks prior to the scheduling of baseline measurements. In addition, all drivers within participating depots will receive a letter and participant information sheet informing them of the study. Following the distribution of the study marketing material, researchers will visit participating depots for one to two days to enable interested drivers to ask any questions about the study before signing up. Upon completion of these visits the researchers will provide a list of drivers' names who have agreed to participate to their Transport Managers who will then schedule time for participating drivers to attend the baseline (and follow-up) measurements.

For peer review only - http://bmjopen.bmj.com/site/about/guidelines.xhtml

Within the UK logistics industry, 1% of HGV drivers are women,[15] and the proportion of female HGV drivers employed by our partner company reflects this national average. Whilst females will be included in the study, due to the small proportion of the workforce they represent, the included sample of females may not enable statistically meaningful comparisons to examine any influences of sex on the intervention. However, the sample recruited, will likely reflect the gender disparities seen in the Logistics and Transport industry nationally and internationally.

For peer review only

Sample size

Our earlier exploratory pre-post study revealed that on average HGV drivers achieve 8786 steps/day across both workdays and non-workdays with a standard deviation of 2919 steps.[20] We have powered this study to look for a difference in step counts (the primary outcome) of 1500 steps/day (equivalent to approximately 15 minutes of moderately paced walking) between the intervention group and control group. Evidence demonstrates a linear association between step counts and a range of morbidity and mortality outcomes, as well as with markers of health status including inflammation and adiposity, insulin sensitivity and HDL cholesterol in adults.[24-26] The linear association between step counts and health outcomes indicate that regardless of an individual's baseline value, even modest increases in daily step counts can yield clinically meaningful health benefits. For example, a difference in daily steps of 1500 steps/day has been associated with around a 5-10% lower risk of all-cause mortality and cardiovascular morbidity and mortality in the general population and in those with a high risk of type 2 diabetes respectively.[27 28] The proposed level of change has been chosen based on findings from our exploratory pre-post intervention,[20] whilst also being clinically meaningful.

For peer review only - http://bmjopen.bmj.com/site/about/guidelines.xhtml

Based on a cluster size of 10, a conservative ICC of 0.05 (as there is no previous data to inform this, we have been informed by recommendations of Campbell et al.[29]), an alpha of 0.05, power of 80% and a coefficient of variation to allow for variation in cluster size of 0.51 (based on partner company data) we will require 110 participants from 11 clusters per arm. From experience in conducting such studies, it is estimated that retention and compliance rates will be approximately 70% at 12-months follow-up; therefore, the sample size will be inflated by 30% to ensure we have adequate power in our final analysis. We will also inflate the number of clusters by 2 to allow for whole cluster drop out. We will therefore recruit 24 clusters with an average of 14 participants per cluster.

Intervention – The SHIFT Programme

The SHIFT programme is a multicomponent lifestyle-behaviour intervention designed to target behaviour changes in physical activity, diet and sitting in HGV drivers. This 6-month intervention, grounded within the Social Cognitive Theory for behaviour change[30] consists of a group-based (4-6 participants) 6-hour structured education session tailored for HGV drivers, delivered by two trained educators. It includes information about physical activity, diet and sitting and risk factors for type 2 diabetes and cardiovascular disease. The educational component is founded on the approach used in the award winning suite of DESMOND programmes, including the PREPARE[31] and Let's Prevent Diabetes programmes,[32] created by researchers at the Leicester Diabetes Centre and used throughout the NHS,[33] whilst being tailored to meet the needs of HGV drivers.[6] Within the education session participants will not be 'taught' in a formal way, but supported to work out knowledge through group discussions and to develop individual goals and plans, based on detailed individual feedback received during their health assessments (see Measurements) to achieve over the 6-month intervention period. The education session is supported by specially developed resources for HGV drivers and participant support

For peer review only - http://bmjopen.bmj.com/site/about/guidelines.xhtml

materials. The session will include the discussion of feasible strategies for participants to increase their physical activity, improve their diet and reduce their sitting time (when not driving) during working and non-working hours. The content of the educational session is summarised in Table 1.

Insert Table 1 about here

During the education session, participants will be provided with a Fitbit® Charge 2 activity tracker and encouraged to use this to set goals (agreed at the session) to gradually increase their physical activity predominately through walking-based activity. The Fitbit® activity tracker will provide participants with information on their daily step counts and will be used as a tool for self-monitoring and self-regulation. Physical activity tracking using step counters (traditionally pedometers) has been associated with significant reductions in BMI and blood pressure, with interventions incorporating goal setting being the most effective.[34]

The education session will adopt the promotion of the "small changes" philosophy using the Specific, Measurable, Attainable, Relevant, and Timely (SMART) principle[35] to encourage participants to gradually build-up their daily activity levels, within the confines of their occupation, to meet the current UK Physical Activity guidelines.[36] For example, participants will be encouraged to establish their own personalised action plan, which may also include making dietary improvements in addition to increases in physical activity, with SMART goals throughout the 6-month intervention. 'Step count challenges' (1-week competitions within intervention depots) will run every 6-weeks throughout the 6-month intervention which will be facilitated by local worksite champions. A "cab workout" will be introduced and practised at the education session and participants will be provided with

For peer review only - http://bmjopen.bmj.com/site/about/guidelines.xhtml

resistance bands and balls, and grip strength dynamometers to take away. Participants will be encouraged to undertake the cab workout during breaks when not permitted to leave their vehicle. Participants will be able to keep the intervention tools beyond the 6-month intervention period, however the company will choose whether to sustain the worksite champion support and step count challenges beyond the 6-month intervention period. A Logic Model detailing the underlying theory behind the intervention components is shown in Figure 2.

The structured education session will be delivered by trained personnel from our partner company and by trained members of the research team. These individuals will be trained and mentored by trainers from the Leicester Diabetes Centre. The education sessions will take place within appropriate training rooms within the intervention depots. Personnel delivering the education sessions within each intervention depot will also be trained to act as a local champion, shown to enhance the effectiveness of worksite physical activity interventions.[37] They will provide ongoing health coach support to intervention participants (during the 6-month intervention period) and be responsible for facilitating the step count challenges.

Insert Figure 2 about here

Control arm

Depots assigned to the usual practice control arm will be asked to continue with their usual care conditions. Participants in the control depots will receive an educational leaflet at the outset detailing the importance of healthy lifestyle behaviours (i.e., undertaking regular physical activity, breaking up periods of prolonged sitting, and consuming a healthy diet) for the promotion of health and well-being. Control participants will be requested to

For peer review only - http://bmjopen.bmj.com/site/about/guidelines.xhtml

complete the same study measurements as those in the intervention worksites, at the same time points. Upon completion of the study, control depots will be provided with all of the educational material provided to the intervention participants as part of the SHIFT programme. As the intervention will be delivered by trained personnel within our partner company, the company may choose to provide the full intervention (including the education session and health coach support) to control depots upon completion of the formal trial.

Allocation to treatment groups

Clusters (depots within the same company) will be randomised at the worksite level into the two study arms (intervention and control, using an allocation ratio of 1:1). Randomisation into the study arms will take place in two batches; initially the first 6 clusters (depots) involved in the internal pilot (see below) will be randomised, and in the second batch all of the remaining clusters will be randomised stratified by depot size. Depots will be classified as either small or large based on the median cluster size of all participating depots. In both batches randomisation will take place upon completion of baseline measurements and will be done by an independent statistician at the Leicester Clinical Trials Unit (CTU).

Measurements

The outcome measurements will be assessed at 3 time points. Baseline measures will occur prior to randomisation of the depots into the 2 study arms. A second set of identical measurements will take place 6-months post randomisation (i.e. just after the completion of the 6-month intervention), and a final set will be taken at 12-months post randomisation to assess the sustainability of the intervention (i.e. 6 months after completion of the formal intervention period, as recommended by the National Obesity Observatory[38]). At the

For peer review only - http://bmjopen.bmj.com/site/about/guidelines.xhtml

baseline assessment, the study will be explained to the participant and written informed consent will be obtained. The measurements will be undertaken in suitable rooms within participating depots by trained researchers and will last between 1.5 and 2 hours per participant. Participants will complete a range of self-report questionnaires and have a series of physiological health assessments taken. All participants will receive detailed feedback on their physiological health assessment measures during each measurement session. In the event that a potential health issue is evident during the health assessments, such as undiagnosed hypertension or high cholesterol levels, participants will be advised to visit their GP for further checks. We will provide participants with a letter to give to their GP which summarises the findings from our point-of-care (blood markers) and automated (blood pressure) measures. Participants will be requested to inform the researchers about the use of any prescribed medications that they commence throughout the study duration which may impact the proposed outcome measures. Participants will be issued with objective monitoring devices to assess their free-living physical activity, sedentary behaviour and sleep, which they will be instructed to wear for eight days following each measurement visit. After eight days, participants will be requested to return these monitors to their depot where they will be collected by a member of the research team.

Primary outcome

The primary outcome will be physical activity, expressed as steps/day, at 12 months post randomisation. Physical activity will be objectively measured using the activPAL micro accelerometer, worn continuously on the anterior aspect of the thigh, for 24 hours/day over eight days during each assessment period. The activPAL provides a valid measure of walking and posture (i.e. sitting and standing) in adults,[39-41] and provides a more accurate measure of physical activity and sitting in occupational drivers in comparison to waist-worn

For peer review only

accelerometers.[42] As the physical activity component of the intervention predominantly includes the promotion of walking based-activity, and as participants will be provided with a Fitbit providing information on daily step counts to set goals to increase their physical activity, steps/day was chosen as the primary physical activity related outcome.

Secondary outcomes

A number of secondary outcomes will be assessed at all measurement time points. The secondary outcomes are described below:

Physical activity and sedentary behaviour

Light and moderate-to-vigorous physical activity (MVPA) will be assessed using the activPAL and the wrist-worn GENEActiv accelerometer, both worn continuously for eight days. The GENEActiv is a lightweight waterproof device, resembling a sports watch, which has been found to be a valid and reliable objective measure of physical activity.[43] Outcomes calculated from the GENEActiv include minutes spent in MVPA, proportion of participants meeting the MVPA guidelines of 150 minutes per/week, total volume of physical activity regardless of intensity, and sleep duration. The accelerometer provides time stamped data so activity at specific times of the day (e.g., during work, after work) will also be extracted.

Sedentary behaviour will also be measured for 8 consecutive days during each assessment period using the activPAL3 micro. The activPAL is regarded as the most accurate method of assessing sitting behaviour in free-living settings,[41 44 45] and is recommended for use in interventions when sitting is an outcome measure.[40] From the

For peer review only - http://bmjopen.bmj.com/site/about/guidelines.xhtml
For peer review only

data provided, we will extract total daily sitting time, work-time and leisure-time sitting, sitting bout durations, and number of transitions between sitting and standing.

Sleep duration, subjective sleepiness and chronotype

Sleep duration and efficiency will be measured objectively using the GENEActiv which has been shown to be an accurate measure of sleep, in addition to physical activity.[46] Subjective sleepiness will be assessed using the Karolinska Sleepiness Scale, shown to be a valid measure of sleepiness when validated against electroencephalography (EEG) and performance outcomes.[47 48] Participants' chronotype will be determined using the short version of the Morningness-Eveningness questionnaire.[49]

Anthropometry, adiposity and blood pressure

Stature (measured at baseline only) and body mass (both assessed without shoes), along with waist and hip circumferences, will be measured using standardised anthropometric techniques by trained research staff. BMI will be calculated as weight (kg)/height (m^2). Body composition (percentage body fat and fat mass) will be assessed via bio-impedance analysis, using Tanita DC-360S body composition scales. We will also measure neck circumference which is a novel marker which links strongly to obstructive sleep apnoea, insulin resistance and cardiovascular disease risk.[50] Blood pressure will be measured from the left arm after a twenty minute period of quiet sitting using an automated recorder (Omron HEM-907), in accordance with current recommendations.[51]

For peer review only - http://bmjopen.bmj.com/site/about/guidelines.xhtml

Biochemical assessments

Finger-prick blood samples will be collected from participants, with participants being requested to fast for ≥4 hours prior to attending each health assessment. The 'A1CNow®+ point-of-care analyser will be used to measure glycated haemoglobin which is a marker of long-term glucose regulation used in clinical care. Additionally, we will use the Cardiochek® point-of-care analyser to measure circulating cholesterol (total, HDL, LDL). Both of these systems are manufactured by PTS Diagnostics and possess analyte validation certificates from the International Federation of Clinical Chemistry and Laboratory Medicine.

Functional fitness

Grip strength will be assessed from both hands using the Takei Hand-Grip dynamometer (Takei Scientific Instruments Co., Ltd; Japan). Reduced muscular strength, as measured by grip strength, is associated with an increased risk of cardiovascular disease, and all-cause and cardiovascular mortality.[52]

Cognitive function and psychophysiological reactivity

The Stroop test will be administered over a five minute period using a validated software package to provide a measure of reaction time, sensitivity to interference and the ability to suppress an automated response - reading colour names in favour of naming the font colour.[53] To examine psychophysiological reactivity, acute stress will be induced using a five-minute mirror-tracing task (Campden Instruments Ltd.), during which measures of blood pressure and heart rate will be taken.[54]

For peer review only - http://bmjopen.bmj.com/site/about/guidelines.xhtml

Work-related psychosocial variables and mental health

A series of self-report measures will be employed to characterise work-related health and mental health: musculoskeletal symptoms will be assessed using the Standardised Nordic Questionnaire;[55] work engagement (characterized by vigour, dedication, and absorption) will be measured using the Utrecht Work Engagement Scale (UWES);[56] occupational fatigue will be measured using the Occupational Fatigue Exhaustion Recovery (OFER 15) scale;[57] job performance[58] and job satisfaction[59] will be measured using single-item 7-point Likert scales; sickness presenteeism will be assessed using a single-item questionnaire; participant's perceptions of work demand and support will be assessed using four subscales from the Health and Safety Executive Management Standards Indicator Tool (HSE MSIT),[60] and driving-related safety behaviour will be assessed using a 6-item measure.[61] Anxiety and depression will be measured using the Hospital Anxiety and Depression Scale (HADS),[62] and Social Isolation will be assessed using the 8-item Social Isolation short form from the Patient-Reported Outcomes Measurement Information System.[63 64] Data on sickness absence will be collected via self-report and employer records and will include frequency and duration of self-certified and certified sickness. Data on sickness absence will be collected from organisational records for 12 months prior to the intervention and for the 6-month intervention and follow-up periods.

Health-related quality of life and health-related resource use

The self-reported EQ5D[65] will be completed by participants during each assessment period to inform the within-trial cost-effectiveness analysis (see cost-effectiveness). Participants will also complete a questionnaire, developed for this study, assessing health-related resource use at the same time points.

For peer review only - http://bmjopen.bmj.com/site/about/guidelines.xhtml

Demographics and additional lifestyle health-related behaviour measures

At baseline we will collect basic demographic information for each participant including their date of birth, sex, ethnicity, highest level of education, marital status, postcode (to determine Index of Multiple Deprivation as an indicator of neighbourhood socio-economic status), working hours, years worked as a HGV driver, and years worked at our partner company. At each follow-up assessment, participants will be asked if there have been any changes in these variables. During each assessment, information on smoking status and typical alcohol intake will be gathered by self-report measures. Dietary quality, including fruit and vegetable intake, will be assessed using a short-form food frequency questionnaire.[66]

Internal pilot

We intend to conduct an internal pilot study using the first six clusters (depots). The internal pilot will examine issues surrounding worksite and participant recruitment, randomisation, compliance to the primary outcome, and retention rates at 6-months following randomisation. After this period we will continue to the full trial if the following progression criteria are met:

- All 24 depots required for the full sample size agree to take part in the study. Six depots will be selected to take part in the internal pilot (three will be randomised to the intervention arm and three to the control arm). This will demonstrate that depot recruitment and intervention delivery is on-track.
- According to our criteria, 84 drivers will need to agree to participate in the internal pilot, based on an average of 14 participants per cluster.
- An average of 75% of drivers opting into the study, randomised into the intervention arm, attend the education session across the 3 intervention depots. This figure is based on

For peer review only

the intervention uptake rate seen in our exploratory pre-post intervention study (87%),[20] whilst also recognising that take-up rates tend to be lower when moving from an efficacy to a larger multi-centre effectiveness trial.

- No more than 20% of participants fail to provide valid data for the primary outcome measure (activPAL-determined step counts) at baseline and at 6 months post randomisation or withdraw/are lost to follow-up during the six-month intervention phase. This threshold is necessary as study power requires total withdrawal or loss to follow-up of no higher than 30% during the six-month intervention and six-month follow-up (12 months post randomisation).

If the final two progression criteria are not fully met, strategies to improve these metrics for the full trial will be discussed with the Trial Steering Committee and the study will progress based upon recommendations from this committee.

Process evaluation

The process evaluation will be used to help explain any discrepancies between expected and observed outcomes, to understand the influence of intervention components and context on the observed outcomes, and to provide insight for any further intervention development and implementation.[22] Throughout the intervention, we will monitor the reach, efficacy, adoption, implementation, and maintenance of the intervention using the RE-AIM framework.[67] We will employ a variety of techniques (e.g., logbooks, questionnaires, interviews and focus groups) to inform our process evaluation. For example, Transport Managers (or their nominated facilitators) and educators/worksite champions from each site will report on a monthly basis if there were any organisational changes (e.g. job changes) or events which may affect participation. Self-report questionnaires provided to study participants will evaluate the various intervention components (e.g. education session, physical activity monitoring tool, cab workout). Interviews and focus groups with

For peer review only - http://bmjopen.bmj.com/site/about/guidelines.xhtml

study participants will further examine engagement in the various components of the intervention, along with any perceived barriers or facilitators to participating in these components. Interviews and focus groups with worksite champions, HR staff, health and safety personnel and logistics timetabling and planning staff will further examine the intervention implementation. We will also document any environmental factors (e.g. movement of personnel between worksites/depots, potential contamination of the intervention through drivers in different groups meeting at service stations/customer distribution centres) that may have an influence on intervention effectiveness. Details of the process evaluation components are included in Table 2.

Insert Table 2 about here

Patient and Public Involvement (PPI)

This trial is the result of an earlier 3-year partnership between the research team and a large transport and logistics company (different to our partner in the present study) in the East Midlands, UK. The preparatory work which informed this study[3 6 16 17] was instigated by the company who requested help in improving the lifestyle behaviours and health of their long-distance drivers who were proving difficult to engage. The SHIFT programme was developed in collaboration with long-distance HGV drivers and health and safety personnel working within the logistics sector. Following pilot testing, the intervention and outcome measures described within this protocol, have been refined following further input from drivers and associated stakeholders. A driver and manager working within the logistics sector will sit on our independent Trial Steering Committee and will provide invaluable insight into the design, set-up, conduct and dissemination of this research. Throughout the trial we will conduct regular PPI events with relevant stakeholders to gain feedback on the trial’s progress. The research team will also continue to work with the

For peer review only - http://bmjopen.bmj.com/site/about/guidelines.xhtml

Chartered Institute of Logistics and Transport to facilitate research dissemination (articles, conferences, workshops) across the logistics and transport sector nationally and internationally.

Data Analysis

Statistical analysis - Internal pilot

The average recruitment rate across depots, proportion of participants providing valid data, and attendance rate at the education sessions will be reported with 95% CI. The point estimates and 95% CIs will be compared to the progression criteria outlined above.

Statistical analysis - Main trial

Average daily steps at 12-months will be compared by group using generalised estimating equation models adjusted for baseline values and waking wear time with an exchangeable correlation structure, which adjusts for clustering. For the primary analysis missing data will not be replaced (complete case analysis) but participants will be included in the intervention group in which their depots were randomised irrespective of the intervention actually received (modified intention-to-treat analysis). We have inflated our sample size by 30% to account for potential loss to follow-up and non-compliance with the primary outcome measure. We will compare the baseline characteristics of those who have complete primary outcome data and those who do not. A sensitivity analysis using multiple imputation will be performed to assess the impact of missing outcome data on the results found and to account for uncertainty associated with imputing data (full intention to treat analysis). The imputation will be carried out using the command MI in Stata. MI replaces missing values with multiple sets of simulated values to complete the data, performs standard analysis on each completed dataset, and adjusts the obtained parameter estimates for missing-data uncertainty using Rubin's rules to combine estimates. The

For peer review only - http://bmjopen.bmj.com/site/about/guidelines.xhtml

effect size will also be assessed by attendance excluding those who did not attend the full intervention (per-protocol analysis). Secondary outcomes and 6-month data will be analysed using similar methodology.

Qualitative analyses

Audio-recordings of interviews and focus groups with drivers, worksite champions, HR staff, health and safety personnel and logistics timetabling and planning staff will be transcribed verbatim and analysed using framework analysis,[68 69] using the RE-AIM framework[67] as the overarching framework.

Cost-effectiveness

The economic analysis will consist of a cost-consequence analysis based on the observed results within the trial period and a cost-effectiveness analysis where differences between groups in the trial will be extrapolated to the longer term. For both analyses, costs in both arms will be estimated from a NHS and Personal Social Services (PSS) perspective (consistent with that used by NICE) as well as a wider public sector perspective. In each analysis, the cost of the SHIFT arm will include an estimate of the cost of the intervention (including the cost of training the educators), generated through a staff questionnaire completed at the end of each education session.

Within-trial analysis

Within the trial, resource use estimates will be collected from participant questionnaires and will include health related resource use as well as absence from employment. The health-related resource use will be based on a variant of the Client Service Receipt Inventory and will include services that this population are likely to utilise such as GPs and Practise nurse appointments, occupational health visitors and counsellors. Costs of

For peer review only - http://bmjopen.bmj.com/site/about/guidelines.xhtml

For peer review only

resources will be calculated by applying published national unit cost estimates (e.g. NHS reference costs or PSSRU Unit costs of health and social care[70 71]), where available, to estimates of relevant resource use.

A range of outcomes will be assessed in the trial including health related quality of life, measured using the EQ5D.[65] The within trial analysis will present incremental results for the primary and secondary outcomes (including EQ5D) in both intervention and control arms and will be compared with the incremental costs measured above. We will also present the results in terms of the differences between the groups in time absent from work. Two analyses will be conducted, one including these productivity losses, the other excluding them. This will allow decision makers to assess the importance of inclusion of these costs in the adoption decision.

Longer-term analysis

It is acknowledged that although there may be short term health benefits from the intervention, the longer-term effects of, for example, increased physical activity on diabetic status and number of cardiovascular events may be more important. We will therefore conduct a brief literature review to identify existing models that link short term endpoints (including physical activity) measured in the trial and longer-term quality of life. We have identified and utilised existing models[72] linking physical activity to Quality Adjusted Life Years (QALYs) previously. These models will be utilised to extrapolate costs and effects of the intervention beyond the trial period to a more appropriate time horizon. If appropriate an Incremental Cost-effectiveness Ratio for the extrapolated period will be reported using the QALY. As with the within-trial analysis, we will conduct analyses where productivity losses are included/excluded to assess the impact on decision making. Costs and effects will be discounted at the prevailing recommended rate (currently 1.5% per annum on both

For peer review only - http://bmjopen.bmj.com/site/about/guidelines.xhtml

costs and effects), but will be the subject of sensitivity analysis to reflect the ongoing uncertainty around appropriate discount rates for public health interventions. To reflect the levels of uncertainty in parameter inputs we will conduct probabilistic sensitivity analyses; this will allow a characterisation of the uncertainty around the adoption decision which we will depict using cost-effectiveness acceptability curves. Sensitivity analyses will be performed to determine the robustness of the results to altering certain assumptions such as the discount rate or inclusion/exclusion of productivity losses.

DISCUSSION

HGV drivers' working environments are not conducive to a healthy lifestyle, despite this they are currently an underserved occupational group in terms of health promotion efforts and exhibit higher than nationally representative rates of obesity and related co-morbidities.[10 17] The health and wellbeing of professional drivers is of public concern given their health impacts the safety of all road users.[5 17] Of concern, obese HGV drivers are approximately 50% more likely to have an accident than normal weight drivers,[73] with accident rates increasing further in HGV drivers suffering from obstructive sleep apnoea, a prevalent condition in this occupational group.[7] The present study will target health-related behaviours of this at-risk and underserved occupational group, with the goal of making a positive long-term impact on long-distance HGV drivers' health. Given the current absence of resources available to tackle health inequalities within the transport sector, it is anticipated that if effective, the SHIFT programme could be scalable as a Continued Professional Competence (CPC) resource for HGV drivers nationally and internationally. This resource will likely be modifiable for use across other driving-related occupations. This could have a long-term impact on professional drivers' health, lead to cost savings within the logistics and transport sector, and ultimately impact road safety for all road users.

For peer review only

To our knowledge, this is the first RCT to examine the impact of a multicomponent intervention targeting individual and environmental barriers faced by HGV drivers to lead a healthy lifestyle. Strengths of the study include the robust RCT design, with randomisation at the depot/cluster level to reduce contamination. The large fully powered sample, objectively derived outcome measures, and six-month follow-up assessment after completion of the SHIFT programme are further strengths, along with the extensive process and economic evaluations.

ETHICS AND DISSEMINATION

Ethical approval was obtained from the Loughborough University Ethics Approvals (Human Participants) Sub-Committee (Reference: R17-P063). Study findings will be disseminated through publications in research and professional journals, through conference presentations at scientific meetings, and to relevant regional and national stakeholders via online media and at dissemination events. Loughborough University are the trial sponsor.

AUTHORS' CONTRIBUTIONS

All authors have made substantial contributions to the concept and design of the study. The study was conceived by SC, VVM, JK, TY, CE, MH, FM, LG, JT, VJ, and GR. VVM, VJ and YLC are the qualified Research Associates on the project. LG and NBJ are the trial statisticians and developed the statistical analysis plan and GR developed the cost-effectiveness plan. The first draft of this manuscript was written by SC with input from all other authors. All authors have edited and critically reviewed the paper for intellectual content and approved the final version of the paper.

For peer review only - http://bmjopen.bmj.com/site/about/guidelines.xhtml

FUNDING STATEMENT

This study is funded by the NIHR Public Health Research Programme (reference: NIHR PHR 15/190/42) and supported by the NIHR Leicester Biomedical Research Centre – Lifestyle theme. The views expressed are those of the authors and not necessarily those of the NHS, the NIHR or the Department of Health and Social Care. Funding to cover intervention costs (Fitbits, cab workout equipment) has been received from the Higher Education Innovation Fund, via the Loughborough University Enterprise Projects Group.

COMPETING INTERESTS STATEMENT

None declared.

WORD COUNT: 5893

REFERENCES

1. Apostolopoulos Y, Sonmez S, Shattell MM, Belzer M. Worksite-induced morbidities among truck drivers in the United States. *AAOHN J* 2010;**58**(7):285-96.

2. Sieber WK, Robinson CF, Birdsey J, Chen GX, Hitchcock EM, Lincoln JE, et al. Obesity and other risk factors: the national survey of U.S. long-haul truck driver health and injury. *Am J Ind Med.* 2014;**57**(6):615-26.

3. Varela-Mato V, O'Shea O, King JA, Yates T, Stensel DJ, Biddle SJ, et al. Cross-sectional surveillance study to phenotype lorry drivers' sedentary behaviours, physical activity and cardio-metabolic health. *BMJ Open* 2017;**7**(6):e013162.

4. Apostolopoulos Y, Sonmez S, Shattell M, Haldeman L, Strack R, Jones V. Barriers to Truck Drivers' Healthy Eating: Environmental Influences and Health Promotion Strategies. *Journal of Workplace Behavioral Health* 2011;**26**:122-43.

For peer review only - http://bmjopen.bmj.com/site/about/guidelines.xhtml

For peer review only

5. Garbarino S, Guglielmi O, Sannita WG, Magnavita N, Lanteri P. Sleep and Mental Health in Truck Drivers: Descriptive Review of the Current Evidence and Proposal of Strategies for Primary Prevention. *Int J Environ Res Public Health*. 2018;**15**(9).

6. Caddick N, Varela-Mato V, Nimmo MA, Clemes S, Yates T, King JA. Understanding the health of lorry drivers in context: A critical discourse analysis. *Health* 2017;**21**(1):38-56.

7. Garbarino S, Durando P, Guglielmi O, Dini G, Bersi F, Fornarino S, et al. Sleep Apnea, Sleep Debt and Daytime Sleepiness Are Independently Associated with Road Accidents. A Cross-Sectional Study on Truck Drivers. *PloS One* 2016;**11**(11):e0166262.

8. Hege A, Lemke MK, Apostolopoulos Y, Sonmez S. Occupational health disparities among U.S. long-haul truck drivers: the influence of work organization and sleep on cardiovascular and metabolic disease risk. *PloS One* 2018;**13**(11):e0207322.

9. Health and Social Care Information Centre. Health Survey for England 2014.

10. Thiese MS, Moffitt G, Hanowski RJ, Kales SN, Porter RJ, Hegmann KT. Commercial Driver Medical Examinations: Prevalence of Obesity, Comorbidities, and Certification Outcomes. *J Occup Environ Med* 2015;**57**(6):659-65.

11. Martin BC, Church TS, Bonnell R, Ben-Joseph R, Borgstadt T. The impact of overweight and obesity on the direct medical costs of truck drivers. *J Occup Environ Med* 2009;**51**(2):180-4.

12. Bigert C, Gustavsson P, Hallqvist J, Hogstedt C, Lewne M, Plato N, et al. Myocardial infarction among professional drivers. *Epidemiology* 2003;**14**(3):333-9.

13. Garbarino S, Guglielmi O, Campus C, Mascialino B, Pizzorni D, Nobili L, et al. Screening, diagnosis, and management of obstructive sleep apnea in dangerous-goods truck drivers: to be aware or not? *Sleep Med* 2016;**25**:98-104.

14. The Freight Transport Association. Logistics Report 2018.

15. The All Party Parliamentary Group for Freight Transport. Barriers to Youth Employment in the Freight Transport Sector, 2015.

For peer review only - http://bmjopen.bmj.com/site/about/guidelines.xhtml

16. The Freight Transport Association. Logistics Report 2015.

17. Ng M, Yousuf B, Bigelow P, Van Eerd D. Effectiveness of health promotion programmes for truck drivers: A systematic review. *Health Education Journal* 2015;**74**(3):270-86.

18. Thiese MS, Effiong AC, Ott U, Passey DG, Arnold ZC, Ronna BB, et al. A Clinical Trial on Weight Loss among Truck Drivers. *Int J Occup Environ Med* 2015;**6**(2):104-12.

19. Gilson ND, Pavey TG, Vandelanotte C, Duncan MJ, Gomersall SR, Trost SG, et al. Chronic disease risks and use of a smartphone application during a physical activity and dietary intervention in Australian truck drivers. *Aust N Z J Public Health* 2016;**40**(1):91-3.

20. Varela Mato V, Caddick N, King JA, Johnson V, Edwardson C, Yates T, et al. The Impact of a Novel Structured Health Intervention for Truckers (SHIFT) on Physical Activity and Cardiometabolic Risk Factors. *J Occup Environ Med* 2018;**60**(4):368-76.

21. Varela Mato V, Caddick N, King JA, Yates T, Stensel DJ, Nimmo MA, et al. A Structured Health Intervention for Truckers (SHIFT): A Process Evaluation of a Pilot Health Intervention in a Transport Company. *J Occup Environ Med* 2018;**60**(4):377-85.

22. Craig P, Dieppe P, Macintyre S, Michie S, Nazareth I, Petticrew M. Developing and evaluating complex interventions: the new Medical Research Council guidance. *Int J Nurs Stud* 2013;**50**(5):587-92.

23. Chan AW, Tetzlaff JM, Altman DG, Laupacis A, Gotzsche PC, Krleza-Jeric K, et al. SPIRIT 2013 statement: defining standard protocol items for clinical trials. *Ann Intern Med* 2013;**158**(3):200-7.

24. Ewald B, Attia J, McElduff P. How many steps are enough? Dose-response curves for pedometer steps and multiple health markers in a community-based sample of older Australians. *J Phys Act Health* 2014;**11**(3):509-18.

For peer review only

For peer review only - http://bmjopen.bmj.com/site/about/guidelines.xhtml

25. Thomson JL, Landry AS, Zoellner JM, Tudor-Locke C, Webster M, Connell C, et al. Several steps/day indicators predict changes in anthropometric outcomes: HUB City Steps. *BMC Public Health* 2012;**12**:983.

26. Dwyer T, Ponsonby AL, Ukoumunne OC, Pezic A, Venn A, Dunstan D, et al. Association of change in daily step count over five years with insulin sensitivity and adiposity: population based cohort study. *BMJ* 2011;**342**:c7249.

27. Dwyer T, Pezic A, Sun C, Cochrane J, Venn A, Srikanth V, et al. Objectively Measured Daily Steps and Subsequent Long Term All-Cause Mortality: The Tasped Prospective Cohort Study. *PloS One* 2015;**10**(11):e0141274.

28. Yates T, Haffner SM, Schulte PJ, Thomas L, Huffman KM, Bales CW, et al. Association between change in daily ambulatory activity and cardiovascular events in people with impaired glucose tolerance (NAVIGATOR trial): a cohort analysis. *Lancet* 2014;**383**(9922):1059-66.

29. Campbell MK, Fayers PM, Grimshaw JM. Determinants of the intracluster correlation coefficient in cluster randomized trials: the case of implementation research. *Clin Trials* 2005;**2**(2):99-107.

30. Bandura A. Health promotion by social cognitive means. *Health Educ Behav* 2004;**31**(2):143-64.

31. Yates T, Davies M, Gorely T, Bull F, Khunti K. Effectiveness of a pragmatic education program designed to promote walking activity in individuals with impaired glucose tolerance: a randomized controlled trial. *Diabetes Care* 2009;**32**(8):1404-10.

32. Gray LJ, Yates T, Troughton J, Khunti K, Davies MJ, Let's Prevent Diabetes T. Engagement, Retention, and Progression to Type 2 Diabetes: A Retrospective Analysis of the Cluster-Randomised "Let's Prevent Diabetes" Trial. *PLoS Medicine* 2016;**13**(7):e1002078.

For peer review only - http://bmjopen.bmj.com/site/about/guidelines.xhtml

33. Davies MJ, Heller S, Skinner TC, Campbell MJ, Carey ME, Cradock S, et al. Effectiveness of the diabetes education and self management for ongoing and newly diagnosed (DESMOND) programme for people with newly diagnosed type 2 diabetes: cluster randomised controlled trial. *BMJ* 2008;**336**(7642):491-5.

34. Bravata DM, Smith-Spangler C, Sundaram V, Gienger AL, Lin N, Lewis R, et al. Using pedometers to increase physical activity and improve health: a systematic review. *JAMA* 2007;**298**(19):2296-304.

35. Doran GT. There's a S.M.A.R.T. Way to Write Management's Goals and Objectives. *Management Review* 1981;**70**(11):35-36.

36. Chief Medical Officers of England, Scotland, Wales, and Northern Ireland. Start active, stay active: a report on physical activity from the four home countries' Chief Medical Officers. Crown copyright, 2011.

37. Conn VS, Hafdahl AR, Cooper PS, Brown LM, Lusk SL. Meta-analysis of workplace physical activity interventions. *Am J Prev Med* 2009;**37**(4):330-9.

38. Cavill N, Roberts K, Rutter H. Standard Evaluation Framework for Physical Activity Interventions. Oxford: National Obesity Observatory, 2012.

39. Ryan CG, Grant PM, Tigbe WW, Granat MH. The validity and reliability of a novel activity monitor as a measure of walking. *Br J Sports Med* 2006;**40**(9):779-84.

40. Kozey-Keadle S, Libertine A, Lyden K, Staudenmayer J, Freedson PS. Validation of wearable monitors for assessing sedentary behavior. *Med Sci Sports Exerc* 2011;**43**(8):1561-7.

41. Atkin AJ, Gorely T, Clemes SA, Yates T, Edwardson C, Brage S, et al. Methods of Measurement in epidemiology: Sedentary Behaviour. *Int J Epidemiol* 2012;41(5):1460-71.

42. Varela Mato V, Yates T, Stensel D, Biddle S, Clemes SA. Concurrent Validity of Actigraph-Determined Sedentary Time Against the Activpal Under Free-Living Conditions

For peer review only - http://bmjopen.bmj.com/site/about/guidelines.xhtml

in a Sample of Bus Drivers. *Measurement in Physical Education and Exercise Science* 2017;**21**(4):212-22.

43. Esliger DW, Rowlands AV, Hurst TL, Catt M, Murray P, Eston RG. Validation of the GENEA Accelerometer. *Med Sci Sports Exerc* 2011;**43**(6):1085-93.

44. Edwardson C, Winkler E, Bodicoat DH, Yates T, Davies M, Dunstan D, et al. Considerations when using the activPAL monitor in field-based research with adult populations. *J Sport Health Sci* 2017;**6**:162–78.

45. Edwardson CL, Rowlands AV, Bunnewell S, Sanders J, Esliger DW, Gorely T, et al. Accuracy of Posture Allocation Algorithms for Thigh- and Waist-Worn Accelerometers. *Med Sci Sports Exerc* 2016;**48**(6):1085-90.

46. Buman MP, Hu F, Newman E, Smeaton AF, Epstein DR. Behavioral Periodicity Detection from 24 h Wrist Accelerometry and Associations with Cardiometabolic Risk and Health-Related Quality of Life. *Biomed Res Int* 2016:4856506.

47. Akerstedt T, Gillberg M. Subjective and objective sleepiness in the active individual. *Int J Neurosci* 1990;**52**(1-2):29-37.

48. Kaida K, Takahashi M, Akerstedt T, Nakata A, Otsuka Y, Haratani T, et al. Validation of the Karolinska sleepiness scale against performance and EEG variables. *Clin Neurophysiol* 2006;**117**(7):1574-81.

49. Horne JA, Ostberg O. A self-assessment questionnaire to determine morningness-eveningness in human circadian rhythms. *Int J Chronobiol* 1976;**4**(2):97-110.

50. Preis SR, Massaro JM, Hoffmann U, D'Agostino RB, Sr., Levy D, Robins SJ, et al. Neck circumference as a novel measure of cardiometabolic risk: the Framingham Heart study. *J Clin Endocrinol Metab* 2010;**95**(8):3701-10.

51. O'Brien E, Asmar R, Beilin L, Imai Y, Mallion JM, Mancia G, et al. European Society of Hypertension recommendations for conventional, ambulatory and home blood pressure measurement. *J Hypertens* 2003;**21**(5):821-48.

For peer review only - http://bmjopen.bmj.com/site/about/guidelines.xhtml

52. Leong DP, Teo KK, Rangarajan S, Lopez-Jaramillo P, Avezum A, Jr., Orlandini A, et al. Prognostic value of grip strength: findings from the Prospective Urban Rural Epidemiology (PURE) study. *Lancet* 2015;**386**(9990):266-73.

53. MacLeod CM. Half a century of research on the Stroop effect: an integrative review. *Psychol Bull* 1991;**109**(2):163-203.

54. Wawrzyniak AJ, Hamer M, Steptoe A, Endrighi R. Decreased reaction time variability is associated with greater cardiovascular responses to acute stress. *Psychophysiology* 2016;**53**:739-48.

55. Kuorinka I, Jonsson B, Kilbom A, Vinterberg H, Biering-Sorensen F, Andersson G, et al. Standardised Nordic questionnaires for the analysis of musculoskeletal symptoms. *Appl Ergon* 1987;**18**(3):233-7.

56. Schaufeli W, Salanova M, González-Romá V, Bakker A. The measurement of Engagement and burnout: A confirmative analytic approach. *Journal of Happiness Studies* 2002;**3**:71-92.

57. Winwood PC, Lushington K, Winefield AH. Further development and validation of the Occupational Fatigue Exhaustion Recovery (OFER) scale. *J Occup Environ Med* 2006;**48**(4):381-9.

58. Bond FW, Bunce D. Job control mediates change in a work reorganization intervention for stress reduction. *J Occup Health Psychol* 2001;**6**(4):290-302.

59. Nagy M. Using a single-item approach to measure facet job satisfaction. *J Org Occup. Psychol.* 2002;**75**:77–86.

60. Health and Safety Executive. Health and Safety Executive Management Standards Indicator Tool, 2013.

61. Huang Y, Roetting M, McDevitt J, Melton D, Smith G. Feedback by technology: Attitudes and opinions of truck drivers. *Transportation Research Part F: Traffic Psychology and Behavior* 2005;**8**:277–97.

For peer review only

For peer review only - http://bmjopen.bmj.com/site/about/guidelines.xhtml

62. Zigmond AS, Snaith RP. The hospital anxiety and depression scale. *Acta Psychiatr Scand.* 1983;**67**(6):361-70.

63. Cella D, Gershon R, Lai JS, Choi S. The future of outcomes measurement: item banking, tailored short-forms, and computerized adaptive assessment. *Qual Life Res* 2007;**16** Suppl 1:133-41.

64. Johnston KL, Lawrence SM, Dodds NE, Yu L, Daley DC, Pilkonis PA. Evaluating PROMIS(R) instruments and methods for patient-centered outcomes research: Patient and provider voices in a substance use treatment setting. *Qual Life Res* 2016;**25**(3):615-24.

65. EuroQol G. EuroQol--a new facility for the measurement of health-related quality of life. *Health Policy* 1990;**16**(3):199-208.

66. Cleghorn CL, Harrison RA, Ransley JK, Wilkinson S, Thomas J, Cade JE. Can a dietary quality score derived from a short-form FFQ assess dietary quality in UK adult population surveys? *Public Health Nutr* 2016; **19**(16):2915-2923.

67. Glasgow RE, Vogt TM, Boles SM. Evaluating the public health impact of health promotion interventions: the RE-AIM framework. *Am J Public Health* 1999;**89**(9):1322-7.

68. Ritchie J, Spencer L. Qualitative data analysis for applied policy research. In: Bryman and Burgess, editor. *Analysing Qualitative Data*. London: Routledge, 1994:173-94.

69. Gale NK, Heath G, Cameron E, Rashid S, Redwood S. Using the framework method for the analysis of qualitative data in multi-disciplinary health research. *BMC Med Res Methodol* 2013;**13**:117.

70. Department of Health. NHS reference costs 2012-2013. London, 2013.

71. Personal Social Services Research Unit. Unit Costs of Health and Social Care 2015, University of Kent, 2015.

For peer review only

For peer review only - http://bmjopen.bmj.com/site/about/guidelines.xhtml

72. Sport England, University of East Anglia's Medical School Health Economics Consulting Group. Model for estimating the Outcomes and Values in the Economics of Sport (MOVES v.2.0), 2016.

73. Anderson JE, Govada M, Steffen TK, Thorne CP, Varvarigou V, Kales SN, et al. Obesity is associated with the future risk of heavy truck crashes among newly recruited commercial drivers. *Accid Anal Prev* 2012;**49**:378-84.

74. Chaiken S. The heuristic model of persuation. In: Zanna M, Olson J, Herman C, editors. *Social influence: the Ontaio symposium.* Fifth Edition ed. NJ: Erlbaum, 1987:3-39.

75. Leventhal H, Meyer D, Nerenz D. The common-sense representation of illness danger. In: Rachman S, editor. *Contributions to Medical Psychology.* Second Edition. New York: Pergamon, 1980: 7–30.

76. Bandura A. Self-efficacy: toward a unifying theory of behavioral change. *Psychol Rev* 1977;**84**(2):191-215.

List of Figures

Figure 1. Trial design and participant flow through the study

Figure 2. Logic model for the SHIFT intervention

For peer review only - http://bmjopen.bmj.com/site/about/guidelines.xhtml

For peer review only

Table 1. Outline of the educational component of the SHIFT programme

Section name	Theoretical underpinning	Main aims and educator activities	Duration (minutes)
Welcome and Introduction		Participants introduced to the SHIFT programme and made aware of both content and style of the session.	10
Driver Story	Dual Process theory,[74] Common Sense Model[75]	Participants asked about their beliefs about how being a HGV driver can affect health, the causes of these health problems and controllability of these.	30
Risks and Health problems	Dual Process theory,[74] Common Sense Model,[75] Social learning theory[76]	Facilitator uses participant stories to support them to work out why they may be at risk of future health problems, and what to do to reduce/ manage risk	55
Physical Activity	Dual Process theory,[74] Social learning theory[76]	Facilitator supports participants to develop knowledge and skills to support confidence to increase personal activity levels, to set personal goals and self-monitor through the use of Fitbits. Introduction and practical demonstration of the 'cab-workout'.	80
Depression, Sleeping, Smoking	Dual Process theory,[74] Social learning theory[76]	Facilitator supports participants to develop strategies to manage depression, poor sleep and smoking	30
Food Choices	Dual Process theory,[74] Social learning theory[76]	Facilitator supports participants to develop knowledge and skills for food choices to reduce cardiovascular risk factors and improve overall health.	90
Self-management plan	Dual Process theory,[74] Social learning theory[76]	Participants supported in developing personal self-management plans.	15
Questions	Common Sense Model,[75] Social learning theory[76]	Facilitator checks all questions raised by participants throughout the programme have been answered and understood.	5
What happens next	Social learning theory[76]	Follow-up care outlined	5

For peer review only

For peer review only - http://bmjopen.bmj.com/site/about/guidelines.xhtml

Table 2. Process evaluation plan for the SHIFT intervention

Areas to measure	General process questions	Data source and data collection method	Total numbers and sampling strategy/timescales
Recruitment	Number of depots/worksites invited to participate, and number agreeing<br><br>Number of possible participants at each depot, number invited/recommended for participation, number opting in to the intervention<br><br>Number of participants opting-out, dropping out and non-compliance to the primary outcome measure	Project records, including the number of drivers within each depot approached<br><br>Depot logs of staff numbers, project records, attendance records at measurements<br><br>Participant attendance records, short questionnaires to explore reasons for non-participation, dropping out and non-compliance	On-going throughout the project
Acceptability of randomisation and measurement tools	How depots feel about being randomised to intervention / control arms<br><br>Did participants find outcome assessments acceptable<br><br>How did participants and logistics timetabling staff experience recruitment and timetabling of outcome assessments	Focus groups with participants<br><br>Interviews with local depot health and safety advisors/HR/timetabling staff	~8 focus groups, or until data saturation is reached, with participants ~1 month following completion of baseline measures<br><br>~8 interviews, or until data saturation is reached, with local depot health and safety advisors/HR/timetabling staff ~1 month after completion of baseline measures in their depots
Intervention acceptability and fidelity - implementation	Was the intervention implemented as planned<br><br>How did participants and logistics timetabling staff experience scheduling the education sessions	Interviews with personnel within our logistics partners who are trained as educators and implemented the education sessions<br><br>Interviews with local worksite champions and timetabling staff within intervention depots<br><br>Participant questionnaires	Interviews with educators, the number of which will depend on the number of educators trained, and timetabling staff immediately following delivery of the education sessions<br><br>Interviews with local champions 3 months into the intervention, immediately following the intervention (6 months), and at 9 and 12 months<br><br>Questionnaires administered after education sessions to participants
Intervention acceptability and fidelity - participation	What proportion of the target group participated in the intervention, and what components of the intervention were preferred, did this differ between males and females	Focus groups with intervention participants<br><br>Attendance logs at education sessions and measurement visits	~8 focus groups, or until data saturation is reached, with participants immediately following completion of the intervention (6 months)

For peer review only - http://bmjopen.bmj.com/site/about/guidelines.xhtml

Areas to measure	General process questions	Data source and data collection method	Total numbers and sampling strategy/timescales
	What strategies were put in place by intervention participants to facilitate behaviour change	Questionnaires and focus groups	Brief questionnaires administered to all intervention participants at 6 months during health assessments
Intervention sustainability	What proportion of the target group maintained any changes in their health behaviours following the 6 month intervention period<br><br>Were there any differences in sustainability between males and females<br><br>Are the company going to continue with the intervention in some way	Focus groups with intervention participants<br><br>Questionnaires<br><br>Interviews with health and safety personnel	~8 focus groups, or until data saturation is reached, with participants at 10 months follow-up (4 months after completion of the intervention.<br><br>Brief questionnaires administered to all intervention participants at 12 months during health assessments<br><br>Interviews at 12 months
Intervention contamination	Did movement of staff (e.g. participants, health and safety personnel) occur from intervention to control depots<br><br>Did intervention drivers interact with control drivers at customer warehouses/distribution centres etc.	Control depots to keep a log of any staff changes<br><br>Focus groups with intervention and control participants	Logs collected upon completion of the 12 month follow-up assessments<br><br>8 focus groups, or until data saturation is reached, with intervention and control participants immediately following completion of the intervention (6 months) and at 10 months follow-up
Unexpected events arising from the study	Did intervention and control participants modify their behaviours based on information provided at the baseline health assessments?<br><br>Did the health assessments prompt GP visits<br><br>Did increased self-awareness of health status and constraints within the job lead to cognitive dissonance<br><br>Did intervention participants change an existing activity-related behaviour for another as a result of participating in the study	Focus groups, interviews and questionnaires delivered to intervention and control participants	Questionnaires delivered to intervention and control participants 1 month after completion of the baseline health assessments<br><br>8 focus groups, or until data saturation is reached, with intervention and control participants immediately following completion of the intervention (6 months) and at 10 months follow-up<br><br>One-to one interviews based on questionnaire and focus group responses at 1 and 10 months

For peer review only - http://bmjopen.bmj.com/site/about/guidelines.xhtml

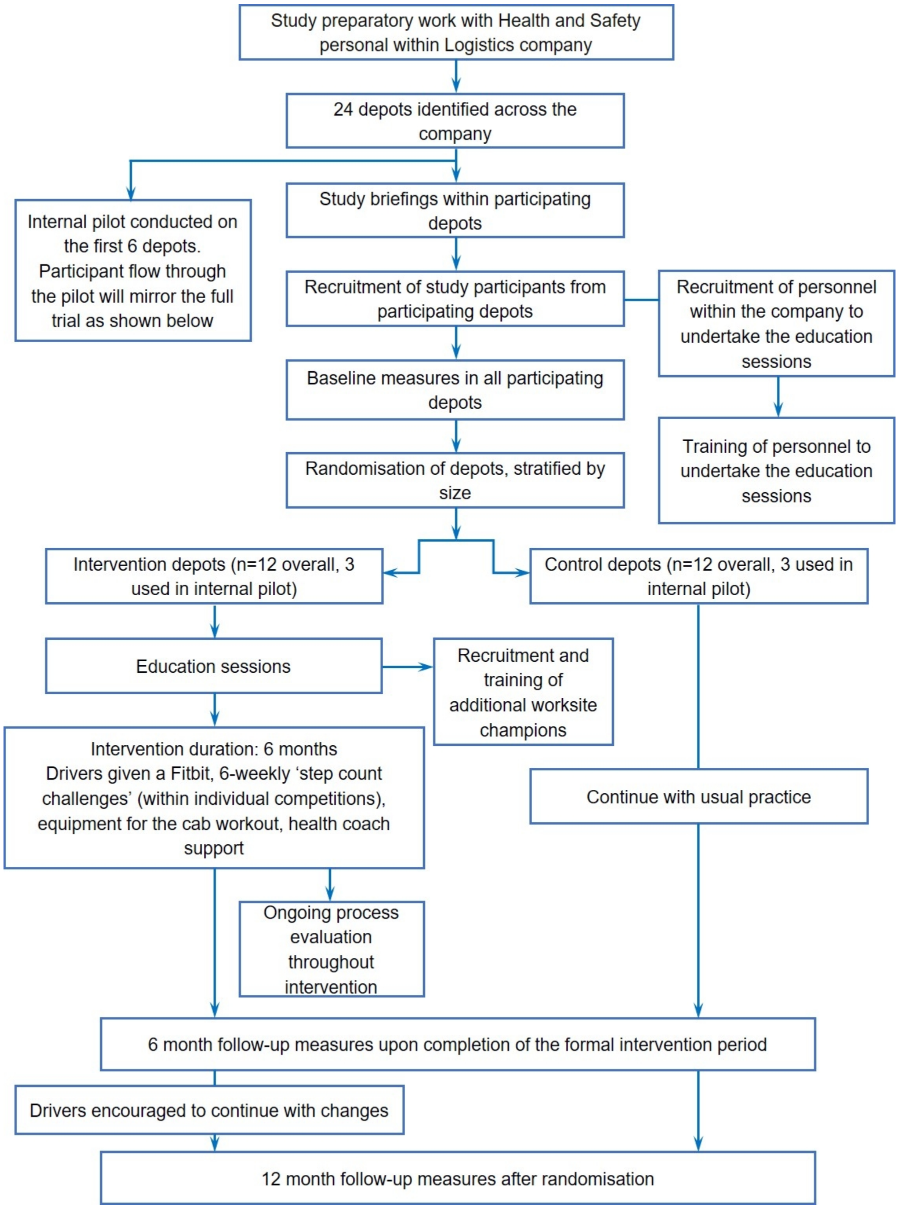

Figure 1. Trial design and participant flow through the study

192x258mm (150 x 150 DPI)

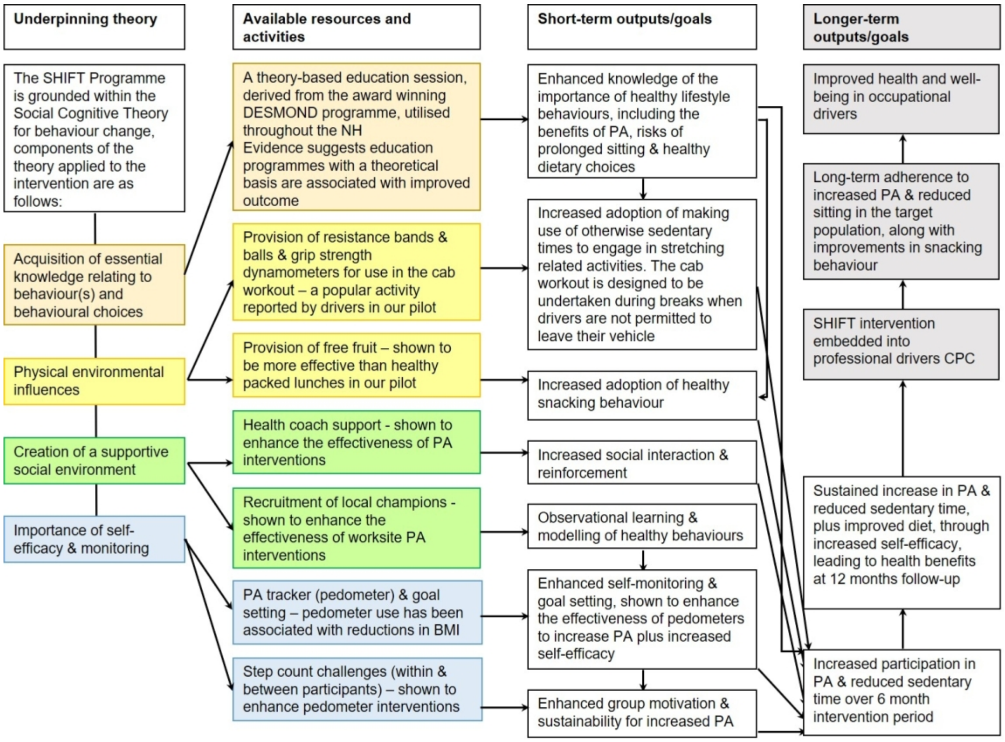

Figure 2. Logic model for the SHIFT intervention

265x195mm (150 x 150 DPI)

STANDARD PROTOCOL ITEMS: RECOMMENDATIONS FOR INTERVENTIONAL TRIALS

SPIRIT 2013 Checklist: Recommended items to address in a clinical trial protocol and related documents*

Section/item	Item No	Description	Addressed on page number
**Administrative information**			
Title	1	Descriptive title identifying the study design, population, interventions, and, if applicable, trial acronym	1
Trial registration	2a	Trial identifier and registry name. If not yet registered, name of intended registry	3
	2b	All items from the World Health Organization Trial Registration Data Set	N/A
Protocol version	3	Date and version identifier	N/A
Funding	4	Sources and types of financial, material, and other support	26 - 27
Roles and responsibilities	5a	Names, affiliations, and roles of protocol contributors	1 & 26
	5b	Name and contact information for the trial sponsor	26
	5c	Role of study sponsor and funders, if any, in study design; collection, management, analysis, and interpretation of data; writing of the report; and the decision to submit the report for publication, including whether they will have ultimate authority over any of these activities	26
	5d	Composition, roles, and responsibilities of the coordinating centre, steering committee, endpoint adjudication committee, data management team, and other individuals or groups overseeing the trial, if applicable (see Item 21a for data monitoring committee)	21

For peer review only

**Introduction**			
Background and rationale	6a	Description of research question and justification for undertaking the trial, including summary of relevant studies (published and unpublished) examining benefits and harms for each intervention	4 - 6
	6b	Explanation for choice of comparators	12
Objectives	7	Specific objectives or hypotheses	6 - 7
Trial design	8	Description of trial design including type of trial (eg, parallel group, crossover, factorial, single group), allocation ratio, and framework (eg, superiority, equivalence, noninferiority, exploratory)	7
**Methods: Participants, interventions, and outcomes**			
Study setting	9	Description of study settings (eg, community clinic, academic hospital) and list of countries where data will be collected. Reference to where list of study sites can be obtained	7
Eligibility criteria	10	Inclusion and exclusion criteria for participants. If applicable, eligibility criteria for study centres and individuals who will perform the interventions (eg, surgeons, psychotherapists)	8
Interventions	11a	Interventions for each group with sufficient detail to allow replication, including how and when they will be administered	10 - 12
	11b	Criteria for discontinuing or modifying allocated interventions for a given trial participant (eg, drug dose change in response to harms, participant request, or improving/worsening disease)	N/A
	11c	Strategies to improve adherence to intervention protocols, and any procedures for monitoring adherence (eg, drug tablet return, laboratory tests)	20
	11d	Relevant concomitant care and interventions that are permitted or prohibited during the trial	N/A
Outcomes	12	Primary, secondary, and other outcomes, including the specific measurement variable (eg, systolic blood pressure), analysis metric (eg, change from baseline, final value, time to event), method of aggregation (eg, median, proportion), and time point for each outcome. Explanation of the clinical relevance of chosen efficacy and harm outcomes is strongly recommended	14 - 19
Participant timeline	13	Time schedule of enrolment, interventions (including any run-ins and washouts), assessments, and visits for participants. A schematic diagram is highly recommended (see Figure)	Figure 1

For peer review only - http://bmjopen.bmj.com/site/about/guidelines.xhtml

Sample size	14	Estimated number of participants needed to achieve study objectives and how it was determined, including clinical and statistical assumptions supporting any sample size calculations	9
Recruitment	15	Strategies for achieving adequate participant enrolment to reach target sample size	9
**Methods: Assignment of interventions (for controlled trials)**			
Allocation:			
Sequence generation	16a	Method of generating the allocation sequence (eg, computer-generated random numbers), and list of any factors for stratification. To reduce predictability of a random sequence, details of any planned restriction (eg, blocking) should be provided in a separate document that is unavailable to those who enrol participants or assign interventions	13
Allocation concealment mechanism	16b	Mechanism of implementing the allocation sequence (eg, central telephone; sequentially numbered, opaque, sealed envelopes), describing any steps to conceal the sequence until interventions are assigned	13
Implementation	16c	Who will generate the allocation sequence, who will enrol participants, and who will assign participants to interventions	13
Blinding (masking)	17a	Who will be blinded after assignment to interventions (eg, trial participants, care providers, outcome assessors, data analysts), and how	3
	17b	If blinded, circumstances under which unblinding is permissible, and procedure for revealing a participant's allocated intervention during the trial	N/A
**Methods: Data collection, management, and analysis**			
Data collection methods	18a	Plans for assessment and collection of outcome, baseline, and other trial data, including any related processes to promote data quality (eg, duplicate measurements, training of assessors) and a description of study instruments (eg, questionnaires, laboratory tests) along with their reliability and validity, if known. Reference to where data collection forms can be found, if not in the protocol	14 - 19
	18b	Plans to promote participant retention and complete follow-up, including list of any outcome data to be collected for participants who discontinue or deviate from intervention protocols	14 – 19, 20

Data management	19	Plans for data entry, coding, security, and storage, including any related processes to promote data quality (eg, double data entry; range checks for data values). Reference to where details of data management procedures can be found, if not in the protocol	N/A
Statistical methods	20a	Statistical methods for analysing primary and secondary outcomes. Reference to where other details of the statistical analysis plan can be found, if not in the protocol	21 - 25
	20b	Methods for any additional analyses (eg, subgroup and adjusted analyses)	N/A
	20c	Definition of analysis population relating to protocol non-adherence (eg, as randomised analysis), and any statistical methods to handle missing data (eg, multiple imputation)	22
**Methods: Monitoring**			
Data monitoring	21a	Composition of data monitoring committee (DMC); summary of its role and reporting structure; statement of whether it is independent from the sponsor and competing interests; and reference to where further details about its charter can be found, if not in the protocol. Alternatively, an explanation of why a DMC is not needed	20 & 21
	21b	Description of any interim analyses and stopping guidelines, including who will have access to these interim results and make the final decision to terminate the trial	19 - 20
Harms	22	Plans for collecting, assessing, reporting, and managing solicited and spontaneously reported adverse events and other unintended effects of trial interventions or trial conduct	N/A
Auditing	23	Frequency and procedures for auditing trial conduct, if any, and whether the process will be independent from investigators and the sponsor	20
**Ethics and dissemination**			
Research ethics approval	24	Plans for seeking research ethics committee/institutional review board (REC/IRB) approval	3
Protocol amendments	25	Plans for communicating important protocol modifications (eg, changes to eligibility criteria, outcomes, analyses) to relevant parties (eg, investigators, REC/IRBs, trial participants, trial registries, journals, regulators)	N/A

For peer review only - http://bmjopen.bmj.com/site/about/guidelines.xhtml

Consent or assent	26a	Who will obtain informed consent or assent from potential trial participants or authorised surrogates, and how (see Item 32)	13
	26b	Additional consent provisions for collection and use of participant data and biological specimens in ancillary studies, if applicable	N/A
Confidentiality	27	How personal information about potential and enrolled participants will be collected, shared, and maintained in order to protect confidentiality before, during, and after the trial	13
Declaration of interests	28	Financial and other competing interests for principal investigators for the overall trial and each study site	26 - 27
Access to data	29	Statement of who will have access to the final trial dataset, and disclosure of contractual agreements that limit such access for investigators	N/A
Ancillary and post-trial care	30	Provisions, if any, for ancillary and post-trial care, and for compensation to those who suffer harm from trial participation	N/A
Dissemination policy	31a	Plans for investigators and sponsor to communicate trial results to participants, healthcare professionals, the public, and other relevant groups (eg, via publication, reporting in results databases, or other data sharing arrangements), including any publication restrictions	26
	31b	Authorship eligibility guidelines and any intended use of professional writers	N/A
	31c	Plans, if any, for granting public access to the full protocol, participant-level dataset, and statistical code	N/A
**Appendices**			
Informed consent materials	32	Model consent form and other related documentation given to participants and authorised surrogates	Supplementary file
Biological specimens	33	Plans for collection, laboratory evaluation, and storage of biological specimens for genetic or molecular analysis in the current trial and for future use in ancillary studies, if applicable	N/A

For peer review only - http://bmjopen.bmj.com/site/about/guidelines.xhtml

BMJ Open

A cluster randomised controlled trial to investigate the effectiveness and cost-effectiveness of a Structured Health Intervention For Truckers (The SHIFT Study): Study Protocol

Journal:	*BMJ Open*
Manuscript ID	bmjopen-2019-030175.R2
Article Type:	Protocol
Date Submitted by the Author:	08-Aug-2019
Complete List of Authors:	Clemes, Stacy; Loughborough University, School of Sport, Exercise and Health Sciences<br>Varela Mato, Verónica; Loughborough University, School of Sport, Exercise and Health Sciences<br>Munir, Fehmidah; Loughborough University, School of Sport, Exercise and Health Sciences<br>Edwardson, Charlotte; University of Leicester, Diabetes Research Centre<br>Chen, Yu-Ling; Loughborough University, School of Sport, Exercise and Health Sciences<br>Hamer, Mark; Loughborough University, School of Sport, Exercise and Health Sciences<br>Gray, Laura; University of Leicester, Department of Health Sciences<br>Bhupendra Jaicim, Nishal; University of Leicester, Leicester Clinical Trials Unit<br>Richardson, Gerry; University of York, Centre for Health Economics<br>Johnson, Vicki; University Hospitals of Leicester NHS Trust, Leicester Diabetes Centre<br>Troughton, Jacqui; University Hospitals of Leicester NHS Trust, Leicester Diabetes Centre<br>Yates, Thomas; University of Leicester, Diabetes Research Centre<br>King, James; Loughborough University, School of Sport, Exercise and Health Sciences
**Primary Subject Heading**:	Sports and exercise medicine
Secondary Subject Heading:	Occupational and environmental medicine, Public health
Keywords:	PUBLIC HEALTH, PREVENTIVE MEDICINE, Physical activity, Sedentary behaviour, Health promotion, Occupational drivers

SCHOLARONE™
Manuscripts

A cluster randomised controlled trial to investigate the effectiveness and cost-effectiveness of a Structured Health Intervention For Truckers (The SHIFT Study): Study Protocol

Stacy A Clemes[1,2], Veronica Varela-Mato[1,2], Fehmidah Munir[1,2], Charlotte L Edwardson[2,3], Yu-Ling Chen[1,2], Mark Hamer[1,2], Laura J Gray[4], Nishal Bhupendra Jaicim[5], Gerry Richardson[6], Vicki Johnson[7], Jacqui Troughton[7], Thomas Yates[2,3], James King[1,2]

[1]National Centre for Sport and Exercise Medicine, School of Sport, Exercise and Health Sciences, Loughborough University, Loughborough, UK

[2]NIHR Leicester Biomedical Research Centre, UK

[3]Diabetes Research Centre, University of Leicester, Leicester, UK

[4]Department of Health Sciences, University of Leicester, Leicester, UK

[5]Leicester Clinical Trials Unit, University of Leicester, Leicester, UK

[6]Centre for Health Economics, University of York, York, UK

[7]Leicester Diabetes Centre, University Hospitals of Leicester NHS Trust, Leicester, UK

Corresponding author: Stacy Clemes

Postal address: National Centre for Sport and Exercise Medicine, School of Sport, Exercise and Health Sciences, Loughborough University, Loughborough, UK

Email: S.A.Clemes@lboro.ac.uk

Telephone number: +44 (0)1509 228170

For peer review only - http://bmjopen.bmj.com/site/about/guidelines.xhtml

ABSTRACT

Introduction: Heavy goods vehicle (HGV) drivers' exhibit higher than nationally representative rates of obesity, and obesity-related co-morbidities, in comparison to other occupational groups. Their working environments are not conducive to a healthy lifestyle, yet there has been limited attention to health promotion efforts. We have developed a Structured Health Intervention For Truckers (the SHIFT programme), a multicomponent, theory-driven, health-behaviour intervention targeting physical activity, diet, and sitting in HGV drivers. This paper describes the protocol of a cluster randomised controlled trial designed to evaluate the effectiveness and cost-effectiveness of the SHIFT programme.

Methods and analysis: HGV drivers will be recruited from a logistics company in the UK. Following baseline measurements, depots (clusters) will be randomised to either the SHIFT intervention or usual-care control arm (12 clusters in each, average cluster size 14 drivers). The 6-month SHIFT intervention includes a group-based interactive 6-hour education session, worksite champion support, and equipment provision (including a Fitbit® and resistance bands/balls to facilitate a 'cab workout'). Objectively measured total daily physical activity (steps/day) will be the primary outcome. Secondary outcomes include: objectively measured light-intensity physical activity and moderate-vigorous physical activity, sitting time, sleep quality, markers of adiposity, blood pressure, and capillary blood markers (HbA1c, LDL-HDL-cholesterol). Self-report questionnaires will examine fruit and vegetable intake, psychosocial and work outcomes, and mental health. Quality of life and resources used (e.g. GP visits) will also be assessed. Measures will be collected at baseline, 6 and 12-months and analysed according to a modified intention-to-treat principle. A full process evaluation and cost-effectiveness analysis will be conducted.

For peer review only - http://bmjopen.bmj.com/site/about/guidelines.xhtml

Ethics and dissemination: Ethical approval was obtained from the Loughborough University Ethics Approvals Sub-Committee (Reference: R17-P063). Study findings will be disseminated through publications in research and professional journals, through conference presentations, and to relevant regional and national stakeholders via online media and at dissemination events.

Trial Registration: ISRCTN10483894 (date registered: 1st March, 2017)

Strengths and limitations of this study

- To our knowledge, this is the first RCT to examine the impact of a multicomponent intervention targeting individual and environmental barriers faced by HGV drivers to lead a healthy lifestyle.
- The trial will involve a full process evaluation and an economic evaluation.
- The primary outcome, physical activity, will be objectively measured.
- Sustainability of the intervention will be examined at 6-months follow-up, following completion of the intervention.
- Due to the nature of the intervention, participants will not be blinded to their treatment arm, and there is a risk that the secondary outcome self-report measures may be susceptible to reporting bias.

For peer review only

INTRODUCTION

Long-distance heavy goods vehicle (HGV) drivers are exposed to a multitude of health-related risk factors associated with their occupation; as a result, lorry driving has been identified as one of the most hazardous working professions.[1 2] Drivers' working environment provides limited opportunities for a healthy lifestyle and unhealthy lifestyle behaviours, such as a lack of physical activity, prolonged periods of sedentary behaviour (sitting), poor diet, a high prevalence of smoking, high volumes of alcohol consumption, and irregular sleeping patterns are widespread among this occupational group.[2-5] Furthermore, long and variable working hours, including shift work, and tight delivery schedules within the Logistics and Transport industry contribute to psychological stress and sleep deprivation,[6] which can lead to metabolic disturbances and further promote the uptake of unhealthy behavioural choices.[2 5-8]

Long-distance drivers exhibit higher than nationally representative rates of obesity, with observational data from a sample of HGV drivers from the UK demonstrating that 84% were overweight or obese, compared to 75% of males aged 45-54 years reported to be overweight/obese nationally.[9] Similar data have been reported from US HGV drivers.[2 10] The high rates of overweight and obesity in HGV drivers elevates their risk of numerous chronic diseases and conditions, including cardiovascular disease, type 2 diabetes, obstructive sleep apnoea and musculoskeletal disorders.[1 2 10-13]

To compound the high-risk health profile observed in long-distance drivers nationally and internationally,[1 2 10-12] the driver population in the UK (n = ~300,000) has been identified as an ageing workforce (mean age: 48 years).[14] A recent Parliamentary report has highlighted the 'demographic time bomb' the UK logistics industry is currently facing and the health impact of an ageing, at-risk, workforce 'driving a vehicle often referred to as 'a 40-tonne

For peer review only - http://bmjopen.bmj.com/site/about/guidelines.xhtml

missile''.[15] The UK logistics sector is also experiencing a short-fall in HGV drivers, with barriers to recruitment including the lack of roadside facilities, medical concerns and long hours of work.[16] Recommendations on how to address this shortfall and attract younger employees to the sector include increasing awareness within the industry of the need to address driver health risks and health behaviours.[15]

A systematic review[17] of health promotion interventions in truck drivers, including only 8 studies, observed that the interventions generally led to improvements in health and health behaviours. However, it was concluded that the strength of the evidence was limited due to poor study designs, with no control groups, small samples and no or limited follow-up periods.[17] Since the publication of the systematic review, recent studies have examined the impacts of a weight loss intervention in US HGV drivers[18] and a smartphone application on physical activity and diet in Australian HGV drivers.[19] Whilst positive findings were observed, the studies were limited by relatively small samples and no comparison groups.

We have developed a Structured Health Intervention For Truckers (the SHIFT programme), a multicomponent, theory driven, health behaviour intervention designed to promote positive lifestyle changes in relation to physical activity, diet, and sitting in HGV drivers. This intervention has been informed by extensive stakeholder engagement, including a qualitative study exploring the perceived barriers to healthy lifestyle behaviours in HGV drivers,[7] an observational study exploring lifestyle health-related behaviours in HGV drivers and markers of health,[3] and a pre-post pilot intervention[20] with full process evaluation.[21] Initial pre-post testing of the intervention revealed the SHIFT programme lead to favourable changes in physical activity and some markers of health.[20] This protocol paper describes a study which will build on our earlier work and generate new knowledge

For peer review only - http://bmjopen.bmj.com/site/about/guidelines.xhtml

For peer review only

on the effectiveness and cost-effectiveness of the SHIFT programme, evaluated using a cluster randomised controlled trial (RCT) design with immediate and extended follow-up. Specifically, we will examine the impact of the SHIFT programme on physical activity, sedentary behaviour, fruit and vegetable intake, adiposity, sleep duration and quality, risk factors for cardio-metabolic disease, psychosocial outcomes and mental health in a sample of HGV drivers.

STUDY AIM AND OBJECTIVES

The aim of this study is to evaluate the effectiveness and cost-effectiveness of the SHIFT programme using a cluster RCT.

Primary objective

To investigate the impact of the SHIFT programme, compared to usual care, on objectively measured physical activity (expressed as steps/day) at 12-months follow-up.

Secondary objectives

To investigate the impact of the SHIFT programme, compared to usual care, at 12-months follow-up on;

1. time spent in light and moderate-to-vigorous physical activity (MVPA)
2. sitting time
3. measures of adiposity (BMI, percent body fat, waist-hip ratio, neck circumference)
4. blood pressure
5. cardiometabolic risk markers (e.g. HBA1c, total cholesterol, HDL-C and LDL-C)
6. fruit and vegetable intake
7. sleep
8. cognitive function and psychophysiological reactivity

For peer review only - http://bmjopen.bmj.com/site/about/guidelines.xhtml

9. psychosocial variables and mental health (e.g. anxiety and depression, work engagement, job performance and satisfaction, presenteeism, sickness absence, health-related quality of life, and driving related safety behaviour)

We will also conduct a full process evaluation (secondary objective 10) and a full economic evaluation (secondary objective 11).

METHODS AND ANALYSIS

Design

The design of this study is based on guidance from the UK Medical Research Council for developing and evaluating complex interventions,[22] and this protocol paper has been prepared following the recommendations within the SPIRIT (Standard Protocol Items for Randomized Trials) statement.[23] This is a workplace two-armed 12-month cluster RCT, which will incorporate an internal pilot, and include both economic and process evaluations. Clusters (different worksites/depots within the same company) will be randomised, following the completion of baseline measurements, to receive either the 'SHIFT programme' or usual care condition. The impact of the intervention will be assessed at 6 and 12-months after randomisation. Figure 1 shows the overall trial design.

Insert Figure 1 about here

Setting

This research will take place within the worksite setting of a major international Logistics and Transport company. The Logistics and Posts Sector is worth approximately £55 billion to the UK economy and currently employs approximately 1.7 million people. Driving is a fundamental occupation within this industry, and drivers and warehouse workers make up the majority of the workforce within the industry.[15]

For peer review only - http://bmjopen.bmj.com/site/about/guidelines.xhtml

Depot recruitment and exclusion criteria

Depots will be included in the study if they contain at least 20 long-distance HGV drivers (see sample size). Depots containing HGV drivers who make many delivery stops, for example, drivers who deliver consumer goods to domestic customers throughout the day will be excluded. For logistical reasons, depots located within the Midlands region of the UK will be recruited. Our partner company has approximately 40 sites, containing approximately 1700 HGV drivers within this region. These sites are a similar size, and have a similar variation in size, to the company's national-level data. During recruitment, depots will be informed that they will have a 50% chance of being randomised to a current practice control condition.

Participant recruitment and exclusion criteria

All HGV drivers within participating depots will be eligible to participate, unless they meet the following exclusion criteria: suffering from clinically diagnosed cardiovascular disease, or mobility limitations that prevent them from increasing their daily activity levels, haemophilia, or have any blood-borne viruses. Posters advertising the study will be placed in participating depots for up to four weeks prior to the scheduling of baseline measurements. In addition, all drivers within participating depots will receive a letter and participant information sheet informing them of the study. Following the distribution of the study marketing material, researchers will visit participating depots for one to two days to enable interested drivers to ask any questions about the study before signing up. Upon completion of these visits the researchers will provide a list of drivers' names who have agreed to participate to their Transport Managers who will then schedule time for participating drivers to attend the baseline (and follow-up) measurements.

For peer review only - http://bmjopen.bmj.com/site/about/guidelines.xhtml

Within the UK logistics industry, 1% of HGV drivers are women,[15] and the proportion of female HGV drivers employed by our partner company reflects this national average. Whilst females will be included in the study, due to the small proportion of the workforce they represent, the included sample of females may not enable statistically meaningful comparisons to examine any influences of sex on the intervention. However, the sample recruited, will likely reflect the gender disparities seen in the Logistics and Transport industry nationally and internationally.

Sample size

Our earlier exploratory pre-post study revealed that on average HGV drivers achieve 8786 steps/day across both workdays and non-workdays with a standard deviation of 2919 steps.[20] We have powered this study to look for a difference in step counts (the primary outcome) of 1500 steps/day (equivalent to approximately 15 minutes of moderately paced walking) between the intervention group and control group. Evidence demonstrates a linear association between step counts and a range of morbidity and mortality outcomes, as well as with markers of health status including inflammation and adiposity, insulin sensitivity and HDL cholesterol in adults.[24-26] The linear association between step counts and health outcomes indicate that regardless of an individual's baseline value, even modest increases in daily step counts can yield clinically meaningful health benefits. For example, a difference in daily steps of 1500 steps/day has been associated with around a 5-10% lower risk of all-cause mortality and cardiovascular morbidity and mortality in the general population and in those with a high risk of type 2 diabetes respectively.[27 28] The proposed level of change has been chosen based on findings from our exploratory pre-post intervention,[20] whilst also being clinically meaningful.

For peer review only - http://bmjopen.bmj.com/site/about/guidelines.xhtml

Based on a cluster size of 10, a conservative ICC of 0.05 (as there is no previous data to inform this, we have been informed by recommendations of Campbell et al.[29]), an alpha of 0.05, power of 80% and a coefficient of variation to allow for variation in cluster size of 0.51 (based on partner company data) we will require 110 participants from 11 clusters per arm. From experience in conducting such studies, it is estimated that retention and compliance rates will be approximately 70% at 12-months follow-up; therefore, the sample size will be inflated by 30% to ensure we have adequate power in our final analysis. We will also inflate the number of clusters by 2 to allow for whole cluster drop out. We will therefore recruit 24 clusters with an average of 14 participants per cluster.

Intervention – The SHIFT Programme

The SHIFT programme is a multicomponent lifestyle-behaviour intervention designed to target behaviour changes in physical activity, diet and sitting in HGV drivers. This 6-month intervention, grounded within the Social Cognitive Theory for behaviour change[30] consists of a group-based (4-6 participants) 6-hour structured education session tailored for HGV drivers, delivered by two trained educators. It includes information about physical activity, diet and sitting and risk factors for type 2 diabetes and cardiovascular disease. The educational component is founded on the approach used in the award winning suite of DESMOND programmes, including the PREPARE[31] and Let's Prevent Diabetes programmes,[32] created by researchers at the Leicester Diabetes Centre and used throughout the NHS,[33] whilst being tailored to meet the needs of HGV drivers.[7] Within the education session participants will not be 'taught' in a formal way, but supported to work out knowledge through group discussions and to develop individual goals and plans, based on detailed individual feedback received during their health assessments (see Measurements) to achieve over the 6-month intervention period. The education session is supported by specially developed resources for HGV drivers and participant support

For peer review only - http://bmjopen.bmj.com/site/about/guidelines.xhtml

materials. The session will include the discussion of feasible strategies for participants to increase their physical activity, improve their diet and reduce their sitting time (when not driving) during working and non-working hours. The content of the educational session is summarised in Table 1.

For peer review only

For peer review only - http://bmjopen.bmj.com/site/about/guidelines.xhtml

Table 1. Outline of the educational component of the SHIFT programme

Section name	Theoretical underpinning	Main aims and educator activities	Duration (minutes)
Welcome and Introduction		Participants introduced to the SHIFT programme and made aware of both content and style of the session.	10
Driver Story	Dual Process theory,[34] Common Sense Model[35]	Participants asked about their beliefs about how being a HGV driver can affect health, the causes of these health problems and controllability of these.	30
Risks and Health problems	Dual Process theory,[34] Common Sense Model,[35] Social learning theory[36]	Facilitator uses participant stories to support them to work out why they may be at risk of future health problems, and what to do to reduce/ manage risk	55
Physical Activity	Dual Process theory,[34] Social learning theory[36]	Facilitator supports participants to develop knowledge and skills to support confidence to increase personal activity levels, to set personal goals and self-monitor through the use of Fitbits. Introduction and practical demonstration of the 'cab-workout'.	80
Depression, Sleeping, Smoking	Dual Process theory,[34] Social learning theory[36]	Facilitator supports participants to develop strategies to manage depression, poor sleep and smoking	30
Food Choices	Dual Process theory,[34] Social learning theory[36]	Facilitator supports participants to develop knowledge and skills for food choices to reduce cardiovascular risk factors and improve overall health.	90
Self-management plan	Dual Process theory,[34] Social learning theory[36]	Participants supported in developing personal self-management plans.	15
Questions	Common Sense Model,[35] Social learning theory[36]	Facilitator checks all questions raised by participants throughout the programme have been answered and understood.	5
What happens next	Social learning theory[36]	Follow-up care outlined	5

During the education session, participants will be provided with a Fitbit® Charge 2 activity tracker and encouraged to use this to set goals (agreed at the session) to gradually increase their physical activity predominately through walking-based activity. The Fitbit® activity tracker will provide participants with information on their daily step counts and will be used as a tool for self-monitoring and self-regulation. Physical activity tracking using step counters (traditionally pedometers) has been associated with significant reductions in BMI and blood pressure, with interventions incorporating goal setting being the most effective.[37]

For peer review only

The education session will adopt the promotion of the "small changes" philosophy using the Specific, Measurable, Attainable, Relevant, and Timely (SMART) principle[38] to encourage participants to gradually build-up their daily activity levels, within the confines of their occupation, to meet the current UK Physical Activity guidelines.[39] For example, participants will be encouraged to establish their own personalised action plan, which may also include making dietary improvements in addition to increases in physical activity, with SMART goals throughout the 6-month intervention. 'Step count challenges' (1-week competitions within intervention depots) will run every 6-weeks throughout the 6-month intervention which will be facilitated by local worksite champions. A "cab workout" will be introduced and practised at the education session and participants will be provided with resistance bands and balls, and grip strength dynamometers to take away. Participants will be encouraged to undertake the cab workout during breaks when not permitted to leave their vehicle. Participants will be able to keep the intervention tools beyond the 6-month intervention period, however the company will choose whether to sustain the worksite champion support and step count challenges beyond the 6-month intervention period. A Logic Model detailing the underlying theory behind the intervention components is shown in Figure 2.

Insert Figure 2 about here

The structured education session will be delivered by trained personnel from our partner company and by trained members of the research team. These individuals will be trained and mentored by trainers from the Leicester Diabetes Centre. The education sessions will take place within appropriate training rooms within the intervention depots. Personnel delivering the education sessions within each intervention depot will also be trained to act as a local champion, shown to enhance the effectiveness of worksite physical activity

For peer review only - http://bmjopen.bmj.com/site/about/guidelines.xhtml

interventions.[40] They will provide ongoing health coach support to intervention participants (during the 6-month intervention period) and be responsible for facilitating the step count challenges.

Control arm

Depots assigned to the usual practice control arm will be asked to continue with their usual care conditions. Participants in the control depots will receive an educational leaflet at the outset detailing the importance of healthy lifestyle behaviours (i.e., undertaking regular physical activity, breaking up periods of prolonged sitting, and consuming a healthy diet) for the promotion of health and well-being. Control participants will be requested to complete the same study measurements as those in the intervention worksites, at the same time points. Upon completion of the study, control depots will be provided with all of the educational material provided to the intervention participants as part of the SHIFT programme. As the intervention will be delivered by trained personnel within our partner company, the company may choose to provide the full intervention (including the education session and health coach support) to control depots upon completion of the formal trial.

Allocation to treatment groups

Clusters (depots within the same company) will be randomised at the worksite level into the two study arms (intervention and control, using an allocation ratio of 1:1). Randomisation into the study arms will take place in two batches; initially the first 6 clusters (depots) involved in the internal pilot (see below) will be randomised, and in the second batch all of the remaining clusters will be randomised stratified by depot size. Depots will be classified as either small or large based on the median cluster size of all participating depots. In both batches randomisation will take place upon completion of

For peer review only

baseline measurements and will be done by an independent statistician at the Leicester Clinical Trials Unit (CTU).

Measurements

The outcome measurements will be assessed at 3 time points. Baseline measures will occur prior to randomisation of the depots into the 2 study arms. A second set of identical measurements will take place 6-months post randomisation (i.e. just after the completion of the 6-month intervention), and a final set will be taken at 12-months post randomisation to assess the sustainability of the intervention (i.e. 6 months after completion of the formal intervention period, as recommended by the National Obesity Observatory[41]). At the baseline assessment, the study will be explained to the participant and written informed consent will be obtained. The measurements will be undertaken in suitable rooms within participating depots by trained researchers and will last between 1.5 and 2 hours per participant. Participants will complete a range of self-report questionnaires and have a series of physiological health assessments taken. All participants will receive detailed feedback on their physiological health assessment measures during each measurement session. In the event that a potential health issue is evident during the health assessments, such as undiagnosed hypertension or high cholesterol levels, participants will be advised to visit their GP for further checks. We will provide participants with a letter to give to their GP which summarises the findings from our point-of-care (blood markers) and automated (blood pressure) measures. Participants will be requested to inform the researchers about the use of any prescribed medications that they commence throughout the study duration which may impact the proposed outcome measures. Participants will be issued with objective monitoring devices to assess their free-living physical activity, sedentary behaviour and sleep, which they will be instructed to wear for eight days following each measurement visit. After eight days, participants will be requested to return

For peer review only - http://bmjopen.bmj.com/site/about/guidelines.xhtml

these monitors to their depot where they will be collected by a member of the research team.

Primary outcome

The primary outcome will be physical activity, expressed as steps/day, at 12 months post randomisation. Physical activity will be objectively measured using the activPAL micro accelerometer, worn continuously on the anterior aspect of the thigh, for 24 hours/day over eight days during each assessment period. The activPAL provides a valid measure of walking and posture (i.e. sitting and standing) in adults,[42-44] and provides a more accurate measure of physical activity and sitting in occupational drivers in comparison to waist-worn accelerometers.[45] As the physical activity component of the intervention predominantly includes the promotion of walking based-activity, and as participants will be provided with a Fitbit providing information on daily step counts to set goals to increase their physical activity, steps/day was chosen as the primary physical activity related outcome.

Secondary outcomes

A number of secondary outcomes will be assessed at all measurement time points. The secondary outcomes are described below:

Physical activity and sedentary behaviour

Light and moderate-to-vigorous physical activity (MVPA) will be assessed using the activPAL and the wrist-worn GENEActiv accelerometer, both worn continuously for eight days. The GENEActiv is a lightweight waterproof device, resembling a sports watch, which has been found to be a valid and reliable objective measure of physical activity.[46] Outcomes calculated from the GENEActiv include minutes spent in MVPA, proportion of

For peer review only

participants meeting the MVPA guidelines of 150 minutes per/week, total volume of physical activity regardless of intensity, and sleep duration. The accelerometer provides time stamped data so activity at specific times of the day (e.g., during work, after work) will also be extracted.

Sedentary behaviour will also be measured for 8 consecutive days during each assessment period using the activPAL3 micro. The activPAL is regarded as the most accurate method of assessing sitting behaviour in free-living settings,[44 47 48] and is recommended for use in interventions when sitting is an outcome measure.[43] From the data provided, we will extract total daily sitting time, work-time and leisure-time sitting, sitting bout durations, and number of transitions between sitting and standing.

Sleep duration, subjective sleepiness and chronotype

Sleep duration and efficiency will be measured objectively using the GENEActiv which has been shown to be an accurate measure of sleep, in addition to physical activity.[49] Subjective sleepiness will be assessed using the Karolinska Sleepiness Scale, shown to be a valid measure of sleepiness when validated against electroencephalography (EEG) and performance outcomes.[50 51] Participants' chronotype will be determined using the short version of the Morningness-Eveningness questionnaire.[52]

Anthropometry, adiposity and blood pressure

Stature (measured at baseline only) and body mass (both assessed without shoes), along with waist and hip circumferences, will be measured using standardised anthropometric

For peer review only - http://bmjopen.bmj.com/site/about/guidelines.xhtml

techniques by trained research staff. BMI will be calculated as weight (kg)/height (m^2). Body composition (percentage body fat and fat mass) will be assessed via bio-impedance analysis, using Tanita DC-360S body composition scales. We will also measure neck circumference which is a novel marker which links strongly to obstructive sleep apnoea, insulin resistance and cardiovascular disease risk.[53] Blood pressure will be measured from the left arm after a twenty minute period of quiet sitting using an automated recorder (Omron HEM-907), in accordance with current recommendations.[54]

Biochemical assessments

Finger-prick blood samples will be collected from participants, with participants being requested to fast for ≥4 hours prior to attending each health assessment. The 'A1CNow®+ point-of-care analyser will be used to measure glycated haemoglobin which is a marker of long-term glucose regulation used in clinical care. Additionally, we will use the Cardiochek® point-of-care analyser to measure circulating cholesterol (total, HDL, LDL). Both of these systems are manufactured by PTS Diagnostics and possess analyte validation certificates from the International Federation of Clinical Chemistry and Laboratory Medicine.

Functional fitness

Grip strength will be assessed from both hands using the Takei Hand-Grip dynamometer (Takei Scientific Instruments Co., Ltd; Japan). Reduced muscular strength, as measured by grip strength, is associated with an increased risk of cardiovascular disease, and all-cause and cardiovascular mortality.[55]

For peer review only - http://bmjopen.bmj.com/site/about/guidelines.xhtml

Cognitive function and psychophysiological reactivity

The Stroop test will be administered over a five minute period using a validated software package to provide a measure of reaction time, sensitivity to interference and the ability to suppress an automated response - reading colour names in favour of naming the font colour.[56] To examine psychophysiological reactivity, acute stress will be induced using a five-minute mirror-tracing task (Campden Instruments Ltd.), during which measures of blood pressure and heart rate will be taken.[57]

Work-related psychosocial variables and mental health

A series of self-report measures will be employed to characterise work-related health and mental health: musculoskeletal symptoms will be assessed using the Standardised Nordic Questionnaire;[58] work engagement (characterized by vigour, dedication, and absorption) will be measured using the Utrecht Work Engagement Scale (UWES);[59] occupational fatigue will be measured using the Occupational Fatigue Exhaustion Recovery (OFER 15) scale;[60] job performance[61] and job satisfaction[62] will be measured using single-item 7-point Likert scales; sickness presenteeism will be assessed using a single-item questionnaire; participant's perceptions of work demand and support will be assessed using four subscales from the Health and Safety Executive Management Standards Indicator Tool (HSE MSIT),[63] and driving-related safety behaviour will be assessed using a 6-item measure.[64] Anxiety and depression will be measured using the Hospital Anxiety and Depression Scale (HADS),[65] and Social Isolation will be assessed using the 8-item Social Isolation short form from the Patient-Reported Outcomes Measurement Information System.[66 67] Data on sickness absence will be collected via self-report and employer

For peer review only - http://bmjopen.bmj.com/site/about/guidelines.xhtml

records and will include frequency and duration of self-certified and certified sickness. Data on sickness absence will be collected from organisational records for 12 months prior to the intervention and for the 6-month intervention and follow-up periods.

Health-related quality of life and health-related resource use

The self-reported EQ5D[68] will be completed by participants during each assessment period to inform the within-trial cost-effectiveness analysis (see cost-effectiveness). Participants will also complete a questionnaire, developed for this study, assessing health-related resource use at the same time points.

Demographics and additional lifestyle health-related behaviour measures

At baseline we will collect basic demographic information for each participant including their date of birth, sex, ethnicity, highest level of education, marital status, postcode (to determine Index of Multiple Deprivation as an indicator of neighbourhood socio-economic status), working hours, years worked as a HGV driver, and years worked at our partner company. At each follow-up assessment, participants will be asked if there have been any changes in these variables. During each assessment, information on smoking status and typical alcohol intake will be gathered by self-report measures. Dietary quality, including fruit and vegetable intake, will be assessed using a short-form food frequency questionnaire.[69]

Internal pilot

We intend to conduct an internal pilot study using the first six clusters (depots). The internal pilot will examine issues surrounding worksite and participant recruitment,

For peer review only - http://bmjopen.bmj.com/site/about/guidelines.xhtml

For peer review only

randomisation, compliance to the primary outcome, and retention rates at 6-months following randomisation. After this period we will continue to the full trial if the following progression criteria are met:

• All 24 depots required for the full sample size agree to take part in the study. Six depots will be selected to take part in the internal pilot (three will be randomised to the intervention arm and three to the control arm). This will demonstrate that depot recruitment and intervention delivery is on-track.

• According to our criteria, 84 drivers will need to agree to participate in the internal pilot, based on an average of 14 participants per cluster.

• An average of 75% of drivers opting into the study, randomised into the intervention arm, attend the education session across the 3 intervention depots. This figure is based on the intervention uptake rate seen in our exploratory pre-post intervention study (87%),[20] whilst also recognising that take-up rates tend to be lower when moving from an efficacy to a larger multi-centre effectiveness trial.

• No more than 20% of participants fail to provide valid data for the primary outcome measure (activPAL-determined step counts) at baseline and at 6 months post randomisation or withdraw/are lost to follow-up during the six-month intervention phase. This threshold is necessary as study power requires total withdrawal or loss to follow-up of no higher than 30% during the six-month intervention and six-month follow-up (12 months post randomisation).

If the final two progression criteria are not fully met, strategies to improve these metrics for the full trial will be discussed with the Trial Steering Committee and the study will progress based upon recommendations from this committee.

For peer review only - http://bmjopen.bmj.com/site/about/guidelines.xhtml

Process evaluation

The process evaluation will be used to help explain any discrepancies between expected and observed outcomes, to understand the influence of intervention components and context on the observed outcomes, and to provide insight for any further intervention development and implementation.[22] Throughout the intervention, we will monitor the reach, efficacy, adoption, implementation, and maintenance of the intervention using the RE-AIM framework.[70] We will employ a variety of techniques (e.g., logbooks, questionnaires, interviews and focus groups) to inform our process evaluation. For example, Transport Managers (or their nominated facilitators) and educators/worksite champions from each site will report on a monthly basis if there were any organisational changes (e.g. job changes) or events which may affect participation. Self-report questionnaires provided to study participants will evaluate the various intervention components (e.g. education session, physical activity monitoring tool, cab workout). Interviews and focus groups with study participants will further examine engagement in the various components of the intervention, along with any perceived barriers or facilitators to participating in these components. Interviews and focus groups with worksite champions, HR staff, health and safety personnel and logistics timetabling and planning staff will further examine the intervention implementation. We will also document any environmental factors (e.g. movement of personnel between worksites/depots, potential contamination of the intervention through drivers in different groups meeting at service stations/customer distribution centres) that may have an influence on intervention effectiveness. Details of the process evaluation components are included in Table 2.

For peer review only - http://bmjopen.bmj.com/site/about/guidelines.xhtml

Table 2. Process evaluation plan for the SHIFT intervention

Areas to measure	General process questions	Data source and data collection method	Total numbers and sampling strategy/timescales
Recruitment	Number of depots/worksites invited to participate, and number agreeing<br><br>Number of possible participants at each depot, number invited/recommended for participation, number opting in to the intervention<br><br>Number of participants opting-out, dropping out and non-compliance to the primary outcome measure	Project records, including the number of drivers within each depot approached<br><br>Depot logs of staff numbers, project records, attendance records at measurements<br><br>Participant attendance records, short questionnaires to explore reasons for non-participation, dropping out and non-compliance	On-going throughout the project
Acceptability of randomisation and measurement tools	How depots feel about being randomised to intervention / control arms<br><br>Did participants find outcome assessments acceptable<br><br>How did participants and logistics timetabling staff experience recruitment and timetabling of outcome assessments	Focus groups with participants<br><br>Interviews with local depot health and safety advisors/HR/timetabling staff	~8 focus groups, or until data saturation is reached, with participants ~1 month following completion of baseline measures<br><br>~8 interviews, or until data saturation is reached, with local depot health and safety advisors/HR/timetabling staff ~1 month after completion of baseline measures in their depots
Intervention acceptability and fidelity - implementation	Was the intervention implemented as planned<br><br>How did participants and logistics timetabling staff experience scheduling the education sessions	Interviews with personnel within our logistics partners who are trained as educators and implemented the education sessions<br><br>Interviews with local worksite champions and timetabling staff within intervention depots<br><br>Participant questionnaires	Interviews with educators, the number of which will depend on the number of educators trained, and timetabling staff immediately following delivery of the education sessions<br><br>Interviews with local champions 3 months into the intervention, immediately following the intervention (6 months), and at 9 and 12 months<br><br>Questionnaires administered after education sessions to participants

For peer review only - http://bmjopen.bmj.com/site/about/guidelines.xhtml

Areas to measure	General process questions	Data source and data collection method	Total numbers and sampling strategy/timescales
Intervention acceptability and fidelity - participation	What proportion of the target group participated in the intervention, and what components of the intervention were preferred, did this differ between males and females<br><br>What strategies were put in place by intervention participants to facilitate behaviour change	Focus groups with intervention participants<br><br>Attendance logs at education sessions and measurement visits<br><br>Questionnaires and focus groups	~8 focus groups, or until data saturation is reached, with participants immediately following completion of the intervention (6 months)<br><br>Brief questionnaires administered to all intervention participants at 6 months during health assessments
Intervention sustainability	What proportion of the target group maintained any changes in their health behaviours following the 6 month intervention period<br><br>Were there any differences in sustainability between males and females<br><br>Are the company going to continue with the intervention in some way	Focus groups with intervention participants<br><br>Questionnaires<br><br>Interviews with health and safety personnel	~8 focus groups, or until data saturation is reached, with participants at 10 months follow-up (4 months after completion of the intervention.<br><br>Brief questionnaires administered to all intervention participants at 12 months during health assessments<br><br>Interviews at 12 months
Intervention contamination	Did movement of staff (e.g. participants, health and safety personnel) occur from intervention to control depots<br><br>Did intervention drivers interact with control drivers at customer warehouses/distribution centres etc.	Control depots to keep a log of any staff changes<br><br>Focus groups with intervention and control participants	Logs collected upon completion of the 12 month follow-up assessments<br><br>8 focus groups, or until data saturation is reached, with intervention and control participants immediately following completion of the intervention (6 months) and at 10 months follow-up
Unexpected events arising from the study	Did intervention and control participants modify their behaviours based on information provided at the baseline health assessments?<br><br>Did the health assessments prompt GP visits<br><br>Did increased self-awareness of health status and constraints within the job lead to cognitive dissonance<br><br>Did intervention participants change an existing activity-related behaviour for another as a result of participating in the study	Focus groups, interviews and questionnaires delivered to intervention and control participants	Questionnaires delivered to intervention and control participants 1 month after completion of the baseline health assessments<br><br>8 focus groups, or until data saturation is reached, with intervention and control participants immediately following completion of the intervention (6 months) and at 10 months follow-up<br><br>One-to one interviews based on questionnaire and focus group responses at 1 and 10 months

For peer review only - http://bmjopen.bmj.com/site/about/guidelines.xhtml

For peer review only

Patient and Public Involvement (PPI)

This trial is the result of an earlier 3-year partnership between the research team and a large transport and logistics company (different to our partner in the present study) in the East Midlands, UK. The preparatory work which informed this study[3 7 16 17] was instigated by the company who requested help in improving the lifestyle behaviours and health of their long-distance drivers who were proving difficult to engage. The SHIFT programme was developed in collaboration with long-distance HGV drivers and health and safety personnel working within the logistics sector. Following pilot testing, the intervention and outcome measures described within this protocol, have been refined following further input from drivers and associated stakeholders. A driver and manager working within the logistics sector will sit on our independent Trial Steering Committee and will provide invaluable insight into the design, set-up, conduct and dissemination of this research. Throughout the trial we will conduct regular PPI events with relevant stakeholders to gain feedback on the trial's progress. The research team will also continue to work with the Chartered Institute of Logistics and Transport to facilitate research dissemination (articles, conferences, workshops) across the logistics and transport sector nationally and internationally.

Data Analysis

Statistical analysis - Internal pilot

The average recruitment rate across depots, proportion of participants providing valid data, and attendance rate at the education sessions will be reported with 95% CI. The point estimates and 95% CIs will be compared to the progression criteria outlined above.

For peer review only - http://bmjopen.bmj.com/site/about/guidelines.xhtml

Statistical analysis - Main trial

Average daily steps at 12-months will be compared by group using generalised estimating equation models adjusted for baseline values and waking wear time with an exchangeable correlation structure, which adjusts for clustering. For the primary analysis missing data will not be replaced (complete case analysis) but participants will be included in the intervention group in which their depots were randomised irrespective of the intervention actually received (modified intention-to-treat analysis). We have inflated our sample size by 30% to account for potential loss to follow-up and non-compliance with the primary outcome measure. We will compare the baseline characteristics of those who have complete primary outcome data and those who do not. A sensitivity analysis using multiple imputation will be performed to assess the impact of missing outcome data on the results found and to account for uncertainty associated with imputing data (full intention to treat analysis). The imputation will be carried out using the command MI in Stata. MI replaces missing values with multiple sets of simulated values to complete the data, performs standard analysis on each completed dataset, and adjusts the obtained parameter estimates for missing-data uncertainty using Rubin's rules to combine estimates. The effect size will also be assessed by attendance excluding those who did not attend the full intervention (per-protocol analysis). Secondary outcomes and 6-month data will be analysed using similar methodology.

Qualitative analyses

Audio-recordings of interviews and focus groups with drivers, worksite champions, HR staff, health and safety personnel and logistics timetabling and planning staff will be transcribed verbatim and analysed using framework analysis,[71 72] using the RE-AIM framework[70] as the overarching framework.

For peer review only - http://bmjopen.bmj.com/site/about/guidelines.xhtml

Cost-effectiveness

The economic analysis will consist of a cost-consequence analysis based on the observed results within the trial period and a cost-effectiveness analysis where differences between groups in the trial will be extrapolated to the longer term. For both analyses, costs in both arms will be estimated from a NHS and Personal Social Services (PSS) perspective (consistent with that used by NICE) as well as a wider public sector perspective. In each analysis, the cost of the SHIFT arm will include an estimate of the cost of the intervention (including the cost of training the educators), generated through a staff questionnaire completed at the end of each education session.

Within-trial analysis

Within the trial, resource use estimates will be collected from participant questionnaires and will include health related resource use as well as absence from employment. The health-related resource use will be based on a variant of the Client Service Receipt Inventory and will include services that this population are likely to utilise such as GPs and Practise nurse appointments, occupational health visitors and counsellors. Costs of resources will be calculated by applying published national unit cost estimates (e.g. NHS reference costs or PSSRU Unit costs of health and social care[73 74]), where available, to estimates of relevant resource use.

A range of outcomes will be assessed in the trial including health related quality of life, measured using the EQ5D.[68] The within trial analysis will present incremental results for the primary and secondary outcomes (including EQ5D) in both intervention and control arms and will be compared with the incremental costs measured above. We will also present the results in terms of the differences between the groups in time absent from work. Two analyses will be conducted, one including these productivity losses, the other

For peer review only - http://bmjopen.bmj.com/site/about/guidelines.xhtml

excluding them. This will allow decision makers to assess the importance of inclusion of these costs in the adoption decision.

Longer-term analysis

It is acknowledged that although there may be short term health benefits from the intervention, the longer-term effects of, for example, increased physical activity on diabetic status and number of cardiovascular events may be more important. We will therefore conduct a brief literature review to identify existing models that link short term endpoints (including physical activity) measured in the trial and longer-term quality of life. We have identified and utilised existing models[75] linking physical activity to Quality Adjusted Life Years (QALYs) previously. These models will be utilised to extrapolate costs and effects of the intervention beyond the trial period to a more appropriate time horizon. If appropriate an Incremental Cost-effectiveness Ratio for the extrapolated period will be reported using the QALY. As with the within-trial analysis, we will conduct analyses where productivity losses are included/excluded to assess the impact on decision making. Costs and effects will be discounted at the prevailing recommended rate (currently 1.5% per annum on both costs and effects), but will be the subject of sensitivity analysis to reflect the ongoing uncertainty around appropriate discount rates for public health interventions. To reflect the levels of uncertainty in parameter inputs we will conduct probabilistic sensitivity analyses; this will allow a characterisation of the uncertainty around the adoption decision which we will depict using cost-effectiveness acceptability curves. Sensitivity analyses will be performed to determine the robustness of the results to altering certain assumptions such as the discount rate or inclusion/exclusion of productivity losses.

For peer review only - http://bmjopen.bmj.com/site/about/guidelines.xhtml

DISCUSSION

HGV drivers' working environments are not conducive to a healthy lifestyle, despite this they are currently an underserved occupational group in terms of health promotion efforts and exhibit higher than nationally representative rates of obesity and related co-morbidities.[10 17] The health and wellbeing of professional drivers is of public concern given their health impacts the safety of all road users.[5 17] Of concern, obese HGV drivers are approximately 50% more likely to have an accident than normal weight drivers,[76] with accident rates increasing further in HGV drivers suffering from obstructive sleep apnoea, a prevalent condition in this occupational group.[8] The present study will target health-related behaviours of this at-risk and underserved occupational group, with the goal of making a positive long-term impact on long-distance HGV drivers' health. Given the current absence of resources available to tackle health inequalities within the transport sector, it is anticipated that if effective, the SHIFT programme could be scalable as a Continued Professional Competence (CPC) resource for HGV drivers nationally and internationally. This resource will likely be modifiable for use across other driving-related occupations. This could have a long-term impact on professional drivers' health, lead to cost savings within the logistics and transport sector, and ultimately impact road safety for all road users.

To our knowledge, this is the first RCT to examine the impact of a multicomponent intervention targeting individual and environmental barriers faced by HGV drivers to lead a healthy lifestyle. Strengths of the study include the robust RCT design, with randomisation at the depot/cluster level to reduce contamination. The large fully powered sample, objectively derived outcome measures, and six-month follow-up assessment after completion of the SHIFT programme are further strengths, along with the extensive process and economic evaluations.

For peer review only - http://bmjopen.bmj.com/site/about/guidelines.xhtml

ETHICS AND DISSEMINATION

Ethical approval was obtained from the Loughborough University Ethics Approvals (Human Participants) Sub-Committee (Reference: R17-P063). Study findings will be disseminated through publications in research and professional journals, through conference presentations at scientific meetings, and to relevant regional and national stakeholders via online media and at dissemination events. Loughborough University are the trial sponsor.

AUTHORS' CONTRIBUTIONS

All authors have made substantial contributions to the concept and design of the study. The study was conceived by SC, VVM, JK, TY, CE, MH, FM, LG, JT, VJ, and GR. VVM, VJ and YLC are the qualified Research Associates on the project. LG and NBJ are the trial statisticians and developed the statistical analysis plan and GR developed the cost-effectiveness plan. The first draft of this manuscript was written by SC with input from all other authors. All authors have edited and critically reviewed the paper for intellectual content and approved the final version of the paper.

FUNDING STATEMENT

This study is funded by the NIHR Public Health Research Programme (reference: NIHR PHR 15/190/42) and supported by the NIHR Leicester Biomedical Research Centre – Lifestyle theme. The views expressed are those of the authors and not necessarily those of the NHS, the NIHR or the Department of Health and Social Care. Funding to cover intervention costs (Fitbits, cab workout equipment) has been received from the Higher Education Innovation Fund, via the Loughborough University Enterprise Projects Group.

For peer review only

COMPETING INTERESTS STATEMENT

None declared.

WORD COUNT: 5893

REFERENCES

1. Apostolopoulos Y, Sonmez S, Shattell MM, Belzer M. Worksite-induced morbidities among truck drivers in the United States. *AAOHN J* 2010;**58**(7):285-96.

2. Sieber WK, Robinson CF, Birdsey J, Chen GX, Hitchcock EM, Lincoln JE, et al. Obesity and other risk factors: the national survey of U.S. long-haul truck driver health and injury. *Am J Ind Med.* 2014;**57**(6):615-26.

3. Varela-Mato V, O'Shea O, King JA, Yates T, Stensel DJ, Biddle SJ, et al. Cross-sectional surveillance study to phenotype lorry drivers' sedentary behaviours, physical activity and cardio-metabolic health. *BMJ Open* 2017;**7**(6):e013162.

4. Apostolopoulos Y, Sonmez S, Shattell M, Haldeman L, Strack R, Jones V. Barriers to Truck Drivers' Healthy Eating: Environmental Influences and Health Promotion Strategies. *Journal of Workplace Behavioral Health* 2011;**26**:122-43.

5. Garbarino S, Guglielmi O, Sannita WG, Magnavita N, Lanteri P. Sleep and Mental Health in Truck Drivers: Descriptive Review of the Current Evidence and Proposal of Strategies for Primary Prevention. *Int J Environ Res Public Health*. 2018;**15**(9).

6. Hege A, Lemke MK, Apostolopoulos Y, Sonmez S. Occupational health disparities among U.S. long-haul truck drivers: the influence of work organization and sleep on cardiovascular and metabolic disease risk. *PloS One* 2018;**13**(11):e0207322.

7. Caddick N, Varela-Mato V, Nimmo MA, Clemes S, Yates T, King JA. Understanding the health of lorry drivers in context: A critical discourse analysis. *Health* 2017;**21**(1):38-56.

For peer review only - http://bmjopen.bmj.com/site/about/guidelines.xhtml

8. Garbarino S, Durando P, Guglielmi O, Dini G, Bersi F, Fornarino S, et al. Sleep Apnea, Sleep Debt and Daytime Sleepiness Are Independently Associated with Road Accidents. A Cross-Sectional Study on Truck Drivers. *PloS One* 2016;**11**(11):e0166262.

9. Health and Social Care Information Centre. Health Survey for England 2014.

10. Thiese MS, Moffitt G, Hanowski RJ, Kales SN, Porter RJ, Hegmann KT. Commercial Driver Medical Examinations: Prevalence of Obesity, Comorbidities, and Certification Outcomes. *J Occup Environ Med* 2015;**57**(6):659-65.

11. Martin BC, Church TS, Bonnell R, Ben-Joseph R, Borgstadt T. The impact of overweight and obesity on the direct medical costs of truck drivers. *J Occup Environ Med* 2009;**51**(2):180-4.

12. Bigert C, Gustavsson P, Hallqvist J, Hogstedt C, Lewne M, Plato N, et al. Myocardial infarction among professional drivers. *Epidemiology* 2003;**14**(3):333-9.

13. Garbarino S, Guglielmi O, Campus C, Mascialino B, Pizzorni D, Nobili L, et al. Screening, diagnosis, and management of obstructive sleep apnea in dangerous-goods truck drivers: to be aware or not? *Sleep Med* 2016;**25**:98-104.

14. The Freight Transport Association. Logistics Report 2018.

15. The All Party Parliamentary Group for Freight Transport. Barriers to Youth Employment in the Freight Transport Sector, 2015.

16. The Freight Transport Association. Logistics Report 2015.

17. Ng M, Yousuf B, Bigelow P, Van Eerd D. Effectiveness of health promotion programmes for truck drivers: A systematic review. *Health Education Journal* 2015;**74**(3):270-86.

18. Thiese MS, Effiong AC, Ott U, Passey DG, Arnold ZC, Ronna BB, et al. A Clinical Trial on Weight Loss among Truck Drivers. *Int J Occup Environ Med* 2015;**6**(2):104-12.

For peer review only

19. Gilson ND, Pavey TG, Vandelanotte C, Duncan MJ, Gomersall SR, Trost SG, et al. Chronic disease risks and use of a smartphone application during a physical activity and dietary intervention in Australian truck drivers. *Aust N Z J Public Health* 2016;**40**(1):91-3.

20. Varela Mato V, Caddick N, King JA, Johnson V, Edwardson C, Yates T, et al. The Impact of a Novel Structured Health Intervention for Truckers (SHIFT) on Physical Activity and Cardiometabolic Risk Factors. *J Occup Environ Med* 2018;**60**(4):368-76.

21. Varela Mato V, Caddick N, King JA, Yates T, Stensel DJ, Nimmo MA, et al. A Structured Health Intervention for Truckers (SHIFT): A Process Evaluation of a Pilot Health Intervention in a Transport Company. *J Occup Environ Med* 2018;**60**(4):377-85.

22. Craig P, Dieppe P, Macintyre S, Michie S, Nazareth I, Petticrew M. Developing and evaluating complex interventions: the new Medical Research Council guidance. *Int J Nurs Stud* 2013;**50**(5):587-92.

23. Chan AW, Tetzlaff JM, Altman DG, Laupacis A, Gotzsche PC, Krleza-Jeric K, et al. SPIRIT 2013 statement: defining standard protocol items for clinical trials. *Ann Intern Med* 2013;**158**(3):200-7.

24. Ewald B, Attia J, McElduff P. How many steps are enough? Dose-response curves for pedometer steps and multiple health markers in a community-based sample of older Australians. *J Phys Act Health* 2014;**11**(3):509-18.

25. Thomson JL, Landry AS, Zoellner JM, Tudor-Locke C, Webster M, Connell C, et al. Several steps/day indicators predict changes in anthropometric outcomes: HUB City Steps. *BMC Public Health* 2012;**12**:983.

26. Dwyer T, Ponsonby AL, Ukoumunne OC, Pezic A, Venn A, Dunstan D, et al. Association of change in daily step count over five years with insulin sensitivity and adiposity: population based cohort study. *BMJ* 2011;**342**:c7249.

For peer review only - http://bmjopen.bmj.com/site/about/guidelines.xhtml

27. Dwyer T, Pezic A, Sun C, Cochrane J, Venn A, Srikanth V, et al. Objectively Measured Daily Steps and Subsequent Long Term All-Cause Mortality: The Tasped Prospective Cohort Study. *PloS One* 2015;**10**(11):e0141274.

28. Yates T, Haffner SM, Schulte PJ, Thomas L, Huffman KM, Bales CW, et al. Association between change in daily ambulatory activity and cardiovascular events in people with impaired glucose tolerance (NAVIGATOR trial): a cohort analysis. *Lancet* 2014;**383**(9922):1059-66.

29. Campbell MK, Fayers PM, Grimshaw JM. Determinants of the intracluster correlation coefficient in cluster randomized trials: the case of implementation research. *Clin Trials* 2005;**2**(2):99-107.

30. Bandura A. Health promotion by social cognitive means. *Health Educ Behav* 2004;**31**(2):143-64.

31. Yates T, Davies M, Gorely T, Bull F, Khunti K. Effectiveness of a pragmatic education program designed to promote walking activity in individuals with impaired glucose tolerance: a randomized controlled trial. *Diabetes Care* 2009;**32**(8):1404-10.

32. Gray LJ, Yates T, Troughton J, Khunti K, Davies MJ, Let's Prevent Diabetes T. Engagement, Retention, and Progression to Type 2 Diabetes: A Retrospective Analysis of the Cluster-Randomised "Let's Prevent Diabetes" Trial. *PLoS Medicine* 2016;**13**(7):e1002078.

33. Davies MJ, Heller S, Skinner TC, Campbell MJ, Carey ME, Cradock S, et al. Effectiveness of the diabetes education and self management for ongoing and newly diagnosed (DESMOND) programme for people with newly diagnosed type 2 diabetes: cluster randomised controlled trial. *BMJ* 2008;**336**(7642):491-5.

34. Chaiken S. The heuristic model of persuation. In: Zanna M, Olson J, Herman C, editors. *Social influence: the Ontaio symposium.* Fifth Edition ed. NJ: Erlbaum, 1987:3-39.

For peer review only - http://bmjopen.bmj.com/site/about/guidelines.xhtml

35. Leventhal H, Meyer D, Nerenz D. The common-sense representation of illness danger. In: Rachman S, editor. *Contributions to Medical Psychology.* Second Edition. New York: Pergamon, 1980: 7–30.

36. Bandura A. Self-efficacy: toward a unifying theory of behavioral change. *Psychol Rev* 1977;**84**(2):191-215.

37. Bravata DM, Smith-Spangler C, Sundaram V, Gienger AL, Lin N, Lewis R, et al. Using pedometers to increase physical activity and improve health: a systematic review. *JAMA* 2007;**298**(19):2296-304.

38. Doran GT. There's a S.M.A.R.T. Way to Write Management's Goals and Objectives. *Management Review* 1981;**70**(11):35-36.

39. Chief Medical Officers of England, Scotland, Wales, and Northern Ireland. Start active, stay active: a report on physical activity from the four home countries' Chief Medical Officers. Crown copyright, 2011.

40. Conn VS, Hafdahl AR, Cooper PS, Brown LM, Lusk SL. Meta-analysis of workplace physical activity interventions. *Am J Prev Med* 2009;**37**(4):330-9.

41. Cavill N, Roberts K, Rutter H. Standard Evaluation Framework for Physical Activity Interventions. Oxford: National Obesity Observatory, 2012.

42. Ryan CG, Grant PM, Tigbe WW, Granat MH. The validity and reliability of a novel activity monitor as a measure of walking. *Br J Sports Med* 2006;**40**(9):779-84.

43. Kozey-Keadle S, Libertine A, Lyden K, Staudenmayer J, Freedson PS. Validation of wearable monitors for assessing sedentary behavior. *Med Sci Sports Exerc* 2011;**43**(8):1561-7.

44. Atkin AJ, Gorely T, Clemes SA, Yates T, Edwardson C, Brage S, et al. Methods of Measurement in epidemiology: Sedentary Behaviour. *Int J Epidemiol* 2012;41(5):1460-71.

45. Varela Mato V, Yates T, Stensel D, Biddle S, Clemes SA. Concurrent Validity of Actigraph-Determined Sedentary Time Against the Activpal Under Free-Living Conditions

For peer review only - http://bmjopen.bmj.com/site/about/guidelines.xhtml

in a Sample of Bus Drivers. *Measurement in Physical Education and Exercise Science* 2017;**21**(4):212-22.

46. Esliger DW, Rowlands AV, Hurst TL, Catt M, Murray P, Eston RG. Validation of the GENEA Accelerometer. *Med Sci Sports Exerc* 2011;**43**(6):1085-93.

47. Edwardson C, Winkler E, Bodicoat DH, Yates T, Davies M, Dunstan D, et al. Considerations when using the activPAL monitor in field-based research with adult populations. *J Sport Health Sci* 2017;**6**:162–78.

48. Edwardson CL, Rowlands AV, Bunnewell S, Sanders J, Esliger DW, Gorely T, et al. Accuracy of Posture Allocation Algorithms for Thigh- and Waist-Worn Accelerometers. *Med Sci Sports Exerc* 2016;**48**(6):1085-90.

49. Buman MP, Hu F, Newman E, Smeaton AF, Epstein DR. Behavioral Periodicity Detection from 24 h Wrist Accelerometry and Associations with Cardiometabolic Risk and Health-Related Quality of Life. *Biomed Res Int* 2016:4856506.

50. Akerstedt T, Gillberg M. Subjective and objective sleepiness in the active individual. *Int J Neurosci* 1990;**52**(1-2):29-37.

51. Kaida K, Takahashi M, Akerstedt T, Nakata A, Otsuka Y, Haratani T, et al. Validation of the Karolinska sleepiness scale against performance and EEG variables. *Clin Neurophysiol* 2006;**117**(7):1574-81.

52. Horne JA, Ostberg O. A self-assessment questionnaire to determine morningness-eveningness in human circadian rhythms. *Int J Chronobiol* 1976;**4**(2):97-110.

53. Preis SR, Massaro JM, Hoffmann U, D'Agostino RB, Sr., Levy D, Robins SJ, et al. Neck circumference as a novel measure of cardiometabolic risk: the Framingham Heart study. *J Clin Endocrinol Metab* 2010;**95**(8):3701-10.

55. O'Brien E, Asmar R, Beilin L, Imai Y, Mallion JM, Mancia G, et al. European Society of Hypertension recommendations for conventional, ambulatory and home blood pressure measurement. *J Hypertens* 2003;**21**(5):821-48.

For peer review only - http://bmjopen.bmj.com/site/about/guidelines.xhtml

55. Leong DP, Teo KK, Rangarajan S, Lopez-Jaramillo P, Avezum A, Jr., Orlandini A, et al. Prognostic value of grip strength: findings from the Prospective Urban Rural Epidemiology (PURE) study. *Lancet* 2015;**386**(9990):266-73.

56. MacLeod CM. Half a century of research on the Stroop effect: an integrative review. *Psychol Bull* 1991;**109**(2):163-203.

57. Wawrzyniak AJ, Hamer M, Steptoe A, Endrighi R. Decreased reaction time variability is associated with greater cardiovascular responses to acute stress. *Psychophysiology* 2016;**53**:739-48.

58. Kuorinka I, Jonsson B, Kilbom A, Vinterberg H, Biering-Sorensen F, Andersson G, et al. Standardised Nordic questionnaires for the analysis of musculoskeletal symptoms. *Appl Ergon* 1987;**18**(3):233-7.

59. Schaufeli W, Salanova M, González-Romá V, Bakker A. The measurement of Engagement and burnout: A confirmative analytic approach. *Journal of Happiness Studies* 2002;**3**:71-92.

60. Winwood PC, Lushington K, Winefield AH. Further development and validation of the Occupational Fatigue Exhaustion Recovery (OFER) scale. *J Occup Environ Med* 2006;**48**(4):381-9.

61. Bond FW, Bunce D. Job control mediates change in a work reorganization intervention for stress reduction. *J Occup Health Psychol* 2001;**6**(4):290-302.

62. Nagy M. Using a single-item approach to measure facet job satisfaction. *J Org Occup. Psychol.* 2002;**75**:77–86.

63. Health and Safety Executive. Health and Safety Executive Management Standards Indicator Tool, 2013.

64. Huang Y, Roetting M, McDevitt J, Melton D, Smith G. Feedback by technology: Attitudes and opinions of truck drivers. *Transportation Research Part F: Traffic Psychology and Behavior* 2005;**8**:277–97.

For peer review only

For peer review only - http://bmjopen.bmj.com/site/about/guidelines.xhtml

65. Zigmond AS, Snaith RP. The hospital anxiety and depression scale. *Acta Psychiatr Scand.* 1983;**67**(6):361-70.

66. Cella D, Gershon R, Lai JS, Choi S. The future of outcomes measurement: item banking, tailored short-forms, and computerized adaptive assessment. *Qual Life Res* 2007;**16** Suppl 1:133-41.

67. Johnston KL, Lawrence SM, Dodds NE, Yu L, Daley DC, Pilkonis PA. Evaluating PROMIS(R) instruments and methods for patient-centered outcomes research: Patient and provider voices in a substance use treatment setting. *Qual Life Res* 2016;**25**(3):615-24.

68. EuroQol G. EuroQol--a new facility for the measurement of health-related quality of life. *Health Policy* 1990;**16**(3):199-208.

69. Cleghorn CL, Harrison RA, Ransley JK, Wilkinson S, Thomas J, Cade JE. Can a dietary quality score derived from a short-form FFQ assess dietary quality in UK adult population surveys? *Public Health Nutr* 2016; **19**(16):2915-2923.

70. Glasgow RE, Vogt TM, Boles SM. Evaluating the public health impact of health promotion interventions: the RE-AIM framework. *Am J Public Health* 1999;**89**(9):1322-7.

71. Ritchie J, Spencer L. Qualitative data analysis for applied policy research. In: Bryman and Burgess, editor. *Analysing Qualitative Data*. London: Routledge, 1994:173-94.

72. Gale NK, Heath G, Cameron E, Rashid S, Redwood S. Using the framework method for the analysis of qualitative data in multi-disciplinary health research. *BMC Med Res Methodol* 2013;**13**:117.

73. Department of Health. NHS reference costs 2012-2013. London, 2013.

74. Personal Social Services Research Unit. Unit Costs of Health and Social Care 2015, University of Kent, 2015.

For peer review only

75. Sport England, University of East Anglia's Medical School Health Economics Consulting Group. Model for estimating the Outcomes and Values in the Economics of Sport (MOVES v.2.0), 2016.

76. Anderson JE, Govada M, Steffen TK, Thorne CP, Varvarigou V, Kales SN, et al. Obesity is associated with the future risk of heavy truck crashes among newly recruited commercial drivers. *Accid Anal Prev* 2012;**49**:378-84.

List of Figures

Figure 1. Trial design and participant flow through the study

Figure 2. Logic model for the SHIFT intervention

For peer review only - http://bmjopen.bmj.com/site/about/guidelines.xhtml

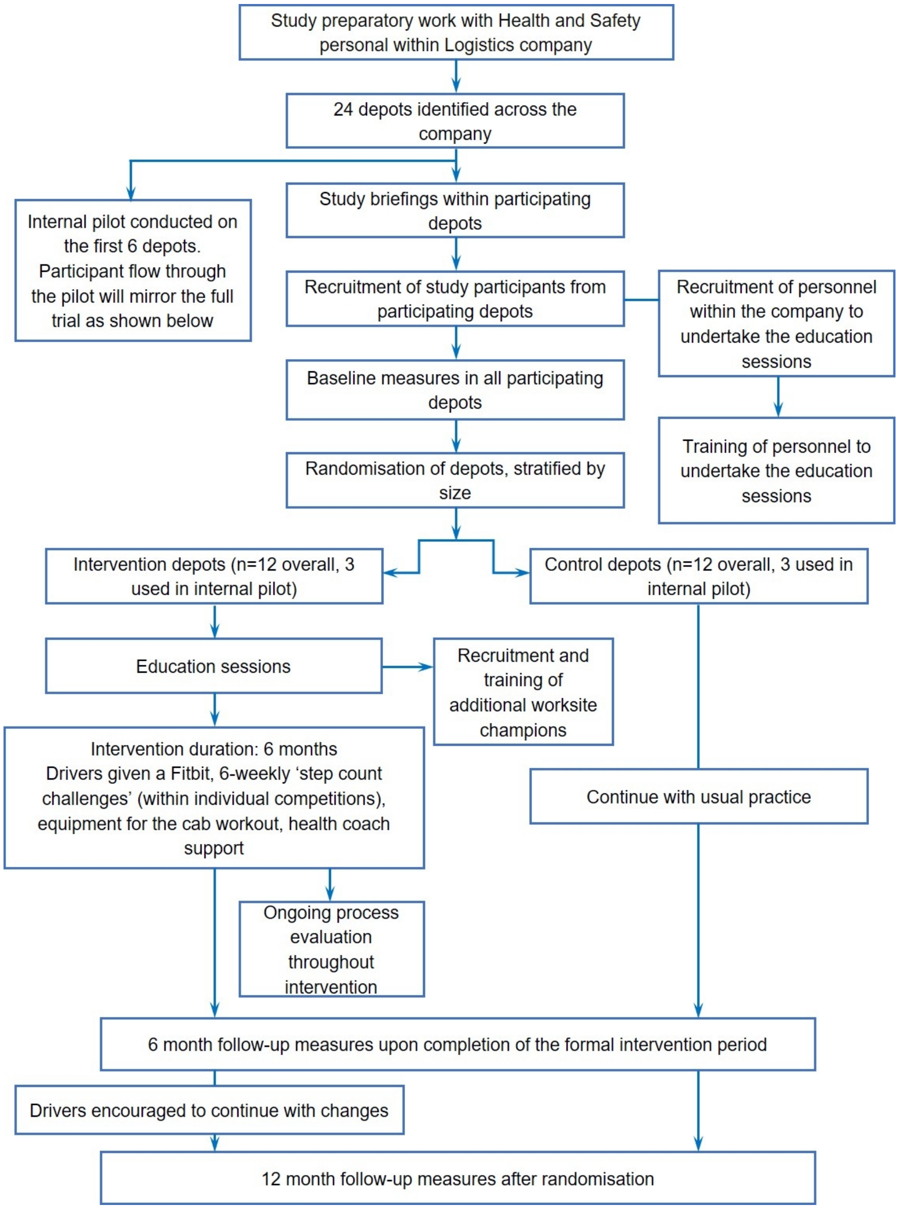

Figure 1. Trial design and participant flow through the study

192x258mm (150 x 150 DPI)

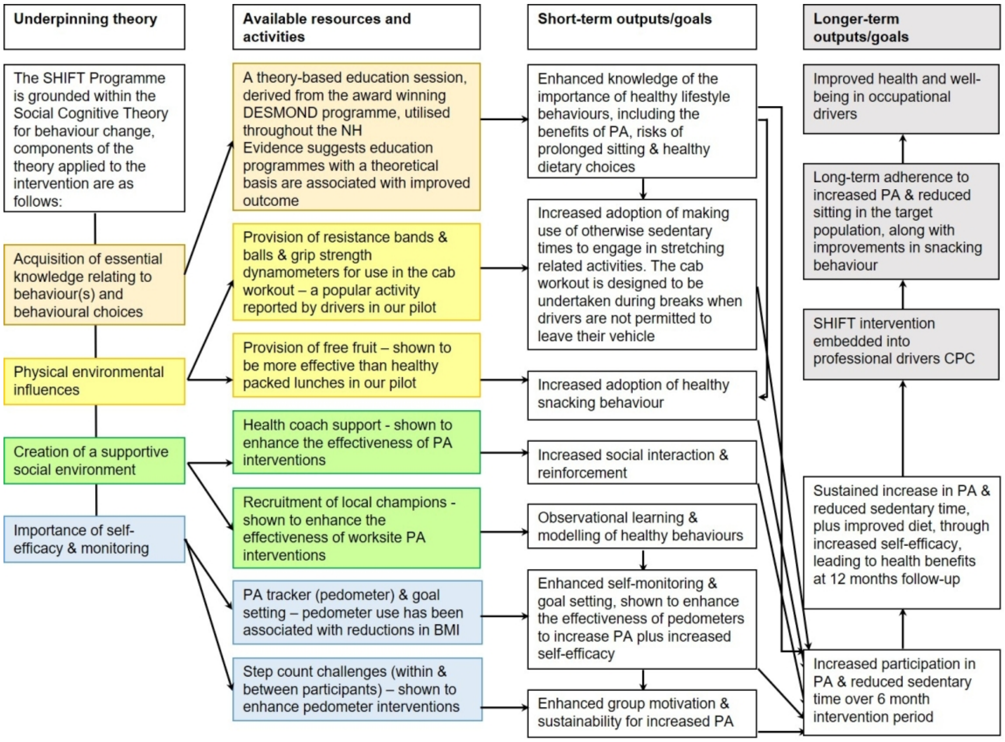

Figure 2. Logic model for the SHIFT intervention

265x195mm (150 x 150 DPI)

SPIRIT
STANDARD PROTOCOL ITEMS: RECOMMENDATIONS FOR INTERVENTIONAL TRIALS

SPIRIT 2013 Checklist: Recommended items to address in a clinical trial protocol and related documents*

Section/item	Item No	Description	Addressed on page number
**Administrative information**			
Title	1	Descriptive title identifying the study design, population, interventions, and, if applicable, trial acronym	1
Trial registration	2a	Trial identifier and registry name. If not yet registered, name of intended registry	3
	2b	All items from the World Health Organization Trial Registration Data Set	N/A
Protocol version	3	Date and version identifier	N/A
Funding	4	Sources and types of financial, material, and other support	26 - 27
Roles and responsibilities	5a	Names, affiliations, and roles of protocol contributors	1 & 26
	5b	Name and contact information for the trial sponsor	26
	5c	Role of study sponsor and funders, if any, in study design; collection, management, analysis, and interpretation of data; writing of the report; and the decision to submit the report for publication, including whether they will have ultimate authority over any of these activities	26
	5d	Composition, roles, and responsibilities of the coordinating centre, steering committee, endpoint adjudication committee, data management team, and other individuals or groups overseeing the trial, if applicable (see Item 21a for data monitoring committee)	21

For peer review only

**Introduction**			
Background and rationale	6a	Description of research question and justification for undertaking the trial, including summary of relevant studies (published and unpublished) examining benefits and harms for each intervention	4 - 6
	6b	Explanation for choice of comparators	12
Objectives	7	Specific objectives or hypotheses	6 - 7
Trial design	8	Description of trial design including type of trial (eg, parallel group, crossover, factorial, single group), allocation ratio, and framework (eg, superiority, equivalence, noninferiority, exploratory)	7
**Methods: Participants, interventions, and outcomes**			
Study setting	9	Description of study settings (eg, community clinic, academic hospital) and list of countries where data will be collected. Reference to where list of study sites can be obtained	7
Eligibility criteria	10	Inclusion and exclusion criteria for participants. If applicable, eligibility criteria for study centres and individuals who will perform the interventions (eg, surgeons, psychotherapists)	8
Interventions	11a	Interventions for each group with sufficient detail to allow replication, including how and when they will be administered	10 - 12
	11b	Criteria for discontinuing or modifying allocated interventions for a given trial participant (eg, drug dose change in response to harms, participant request, or improving/worsening disease)	N/A
	11c	Strategies to improve adherence to intervention protocols, and any procedures for monitoring adherence (eg, drug tablet return, laboratory tests)	20
	11d	Relevant concomitant care and interventions that are permitted or prohibited during the trial	N/A
Outcomes	12	Primary, secondary, and other outcomes, including the specific measurement variable (eg, systolic blood pressure), analysis metric (eg, change from baseline, final value, time to event), method of aggregation (eg, median, proportion), and time point for each outcome. Explanation of the clinical relevance of chosen efficacy and harm outcomes is strongly recommended	14 - 19
Participant timeline	13	Time schedule of enrolment, interventions (including any run-ins and washouts), assessments, and visits for participants. A schematic diagram is highly recommended (see Figure)	Figure 1

For peer review only - http://bmjopen.bmj.com/site/about/guidelines.xhtml

Sample size	14	Estimated number of participants needed to achieve study objectives and how it was determined, including clinical and statistical assumptions supporting any sample size calculations	9
Recruitment	15	Strategies for achieving adequate participant enrolment to reach target sample size	9

Methods: Assignment of interventions (for controlled trials)

Allocation:

Sequence generation	16a	Method of generating the allocation sequence (eg, computer-generated random numbers), and list of any factors for stratification. To reduce predictability of a random sequence, details of any planned restriction (eg, blocking) should be provided in a separate document that is unavailable to those who enrol participants or assign interventions	13
Allocation concealment mechanism	16b	Mechanism of implementing the allocation sequence (eg, central telephone; sequentially numbered, opaque, sealed envelopes), describing any steps to conceal the sequence until interventions are assigned	13
Implementation	16c	Who will generate the allocation sequence, who will enrol participants, and who will assign participants to interventions	13
Blinding (masking)	17a	Who will be blinded after assignment to interventions (eg, trial participants, care providers, outcome assessors, data analysts), and how	3
	17b	If blinded, circumstances under which unblinding is permissible, and procedure for revealing a participant's allocated intervention during the trial	N/A

Methods: Data collection, management, and analysis

Data collection methods	18a	Plans for assessment and collection of outcome, baseline, and other trial data, including any related processes to promote data quality (eg, duplicate measurements, training of assessors) and a description of study instruments (eg, questionnaires, laboratory tests) along with their reliability and validity, if known. Reference to where data collection forms can be found, if not in the protocol	14 - 19
	18b	Plans to promote participant retention and complete follow-up, including list of any outcome data to be collected for participants who discontinue or deviate from intervention protocols	14 – 19, 20

Data management	19	Plans for data entry, coding, security, and storage, including any related processes to promote data quality (eg, double data entry; range checks for data values). Reference to where details of data management procedures can be found, if not in the protocol	N/A
Statistical methods	20a	Statistical methods for analysing primary and secondary outcomes. Reference to where other details of the statistical analysis plan can be found, if not in the protocol	21 - 25
	20b	Methods for any additional analyses (eg, subgroup and adjusted analyses)	N/A
	20c	Definition of analysis population relating to protocol non-adherence (eg, as randomised analysis), and any statistical methods to handle missing data (eg, multiple imputation)	22
**Methods: Monitoring**			
Data monitoring	21a	Composition of data monitoring committee (DMC); summary of its role and reporting structure; statement of whether it is independent from the sponsor and competing interests; and reference to where further details about its charter can be found, if not in the protocol. Alternatively, an explanation of why a DMC is not needed	20 & 21
	21b	Description of any interim analyses and stopping guidelines, including who will have access to these interim results and make the final decision to terminate the trial	19 - 20
Harms	22	Plans for collecting, assessing, reporting, and managing solicited and spontaneously reported adverse events and other unintended effects of trial interventions or trial conduct	N/A
Auditing	23	Frequency and procedures for auditing trial conduct, if any, and whether the process will be independent from investigators and the sponsor	20
**Ethics and dissemination**			
Research ethics approval	24	Plans for seeking research ethics committee/institutional review board (REC/IRB) approval	3
Protocol amendments	25	Plans for communicating important protocol modifications (eg, changes to eligibility criteria, outcomes, analyses) to relevant parties (eg, investigators, REC/IRBs, trial participants, trial registries, journals, regulators)	N/A

Consent or assent	26a	Who will obtain informed consent or assent from potential trial participants or authorised surrogates, and how (see Item 32)	13
	26b	Additional consent provisions for collection and use of participant data and biological specimens in ancillary studies, if applicable	N/A
Confidentiality	27	How personal information about potential and enrolled participants will be collected, shared, and maintained in order to protect confidentiality before, during, and after the trial	13
Declaration of interests	28	Financial and other competing interests for principal investigators for the overall trial and each study site	26 - 27
Access to data	29	Statement of who will have access to the final trial dataset, and disclosure of contractual agreements that limit such access for investigators	N/A
Ancillary and post-trial care	30	Provisions, if any, for ancillary and post-trial care, and for compensation to those who suffer harm from trial participation	N/A
Dissemination policy	31a	Plans for investigators and sponsor to communicate trial results to participants, healthcare professionals, the public, and other relevant groups (eg, via publication, reporting in results databases, or other data sharing arrangements), including any publication restrictions	26
	31b	Authorship eligibility guidelines and any intended use of professional writers	N/A
	31c	Plans, if any, for granting public access to the full protocol, participant-level dataset, and statistical code	N/A
**Appendices**			
Informed consent materials	32	Model consent form and other related documentation given to participants and authorised surrogates	Supplementary file
Biological specimens	33	Plans for collection, laboratory evaluation, and storage of biological specimens for genetic or molecular analysis in the current trial and for future use in ancillary studies, if applicable	N/A

For peer review only
